# Supplementary material for: A pig BodyMap transcriptome reveals diverse tissue physiologies and evolutionary dynamics of transcription
Source: Nat Commun. 2021 Jun 17;12:3715. doi: 10.1038/s41467-021-23560-8 (PMC8211698; doi:10.1038/s41467-021-23560-8)
Supplement: Supplementary file 1 — Supplementary Information [file 41467_2021_23560_MOESM1_ESM.pdf]

Supplementary Information

Supplementary Information Table of Contents

Supplementary Figs. 1-41

Supplementary Fig. 1. Comparison of size, chromosome number, and transcribed features of reference genomes of pig, human, and other common animal models.
Supplementary Fig. 2. Workflow for reconstruction of pig transcriptome.
Supplementary Fig. 3. Mapping of rRNA-depleted RNA-seq data and small RNA-seq data.
Supplementary Fig. 4. Quality assessment of the long transcript assemblies.
Supplementary Fig. 5. Classification of lncRNA biotypes based on their location with respect to PCGs.
Supplementary Fig. 6. Comparative analysis between lncRNAs in this study and other catalogs.
Supplementary Fig. 7. Features of three long transcript types (*i.e.,* PCGs, TUCPs, and lncRNAs).
Supplementary Fig. 8. Features of circRNAs.
Supplementary Fig. 9. Expression distribution of five types of transcripts.
Supplementary Fig. 10. Characterization of the top 0.5% of highly abundant transcripts.
Supplementary Fig. 11. Transcriptional profiling of distinct transcript types across tissues.
Supplementary Fig. 12. Expression profiling of tissues derived from three germ layers.
Supplementary Fig. 13. 3D genome landscape of chromatin organization.
Supplementary Fig. 14. Carcass lean meat percentage for different pig breeds.
Supplementary Fig. 15. Transcriptional heterogeneity among SMTs.
Supplementary Fig. 16. Hierarchical clustering of SMTs in different anatomical regions based on *HOX* genes and homeobox family genes.
Supplementary Fig. 17. Transcriptional patterns of myokines across SMTs.
Supplementary Fig. 18. Transcriptional patterns of NMJ-related PCGs across SMTs from different anatomical regions.
Supplementary Fig. 19. Transcriptional patterns of *MYH*s across SMTs.
Supplementary Fig. 20. Function of putative fiber-specific genes.
Supplementary Fig. 21. Dimensionality reduction and clustering of spots from psoas major (PM) muscle to classify type I and II myofibers.
Supplementary Fig. 22. Dimensionality reduction and clustering of spots to classify the type IIA and IIB myofibers.
Supplementary Fig. 23. Assessment of homogeneity of myofibers/spots based on local diversity of myofiber types.

Supplementary Fig. 24. Marker gene expression in representative spots for three types of myofiber clusters.

Supplementary Fig. 25. Verification of the estimated myofiber proportions using ATPase staining of SMTs.

Supplementary Fig. 26. Transcriptional divergence of distinct transcript types across adipose tissues (ATs).

Supplementary Fig. 27. Estimates of the proportions of different cell types in AT.

Supplementary Fig. 28. Transcriptional divergence of inflammation-related characteristics across ATs.

Supplementary Fig. 29. Transcriptional patterns of *HOX* genes and homeobox family genes suggesting the developmental origins of ATs.

Supplementary Fig. 30. Functional enrichment of tissue-specific mt-localized PCGs.

Supplementary Fig. 31. Heatmap showing the transcriptional patterns of OXPHOS-related mt-localized nu-PCGs across all tissues.

Supplementary Fig. 32. Transcriptional pattern of OXPHOS-related PCGs across SMTs from different anatomical regions.

Supplementary Fig. 33. Heatmap of transcriptional patterns of FAO-related mt-localized nu-PCGs across all tissues.

Supplementary Fig. 34. Comparison of variation between species (nine mammals and chicken) and tissues revealed by (a) gene transcription and (b) alternative splicing patterns.

Supplementary Fig. 35. Global patterns of gene transcription and alternative splicing.

Supplementary Fig. 36. Correlation-based transcription levels potentially reflecting divergence within mammals.

Supplementary Fig. 37. Gene transcription phylogenies for 7 tissues across 9 mammals.

Supplementary Fig. 38. Characteristics of promoter–enhancer interactions (PEIs).

Supplementary Fig. 39. Landscape of enhancer correlations across species.

Supplementary Fig. 40. Transcribed genes with evolutionarily stable and variable transcription levels were identified based on their coefficient of variation across species.

Supplementary Fig. 41. Genes with distinct transcriptional changes in each species and tissue.

Supplementary Tables 1-6

Supplementary Table 1. Sample and data information for pig adipose Hi-C.

Supplementary Table 2. Overview of ST in this study, as well as information for data quantification.

Supplementary Table 3. Sample information for Hi-C and RNA-seq of adipose tissue in the other 6 species.

Supplementary Table 4. Hi-C data for adipose tissue in 6 other species.

Supplementary Table 5. RNA-seq data for adipose tissue in 6 other species.

Supplementary Table 6. Number of PEIs in each species.

Supplementary Methods

1. Pig transcriptome reconstruction

1.1 Animals

1.2 Sample collection

1.3 Construction of rRNA-depleted RNA-seq libraries and data processing

1.4 Pig transcriptome *de novo* assembly

1.5 Coding potential assessment and lncRNA and TUCP identification

1.6 circRNA identification

1.7 Small RNA library sequencing and miRNA annotation

1.8 Gene expression quantification

1.9 Transcriptome complexity analysis

2. 3D genome structure and chromatin information annotation for each transcript

2.1 Samples

2.2 *In situ* Hi-C protocol

2.3 Hi-C data processing and analysis

2.4 Features of the pig genome

2.5 Compartment A/B identification

2.6 Identification of topologically associated domains

2.7 Chromatin 3D modeling

2.8 Gene co-expression associated with TADs

3. Gene transcriptional profiling across tissues

3.1 Tissue clustering

3.2 Differential gene expression analysis across tissues

3.3 Tissue-specific expression analysis

3.4 Functional enrichment analysis

4 Spatiotemporal transcriptomics of SMTs

4.1 Spatial transcriptomic (ST) experiments

4.2 ST sequencing data processing and analysis

4.3 Estimation of the myofiber proportions in bulk RNA-seq data

4.4 Verification of myofiber proportions using ATPase staining

5 Comparative transcriptomic analysis

5.1 Single-copy orthologous PCG identification

5.2 Analyses of alternative splicing

5.3 Characterizing evolutionary gene transcription patterns

6. Gene transcription divergence and PEIs across species

6.1 Samples

6.2 Identification of promoter-enhancer interactions (PEIs)

6.3 Evolutionary divergence in gene transcription driven by PEIs

Supplementary Figures

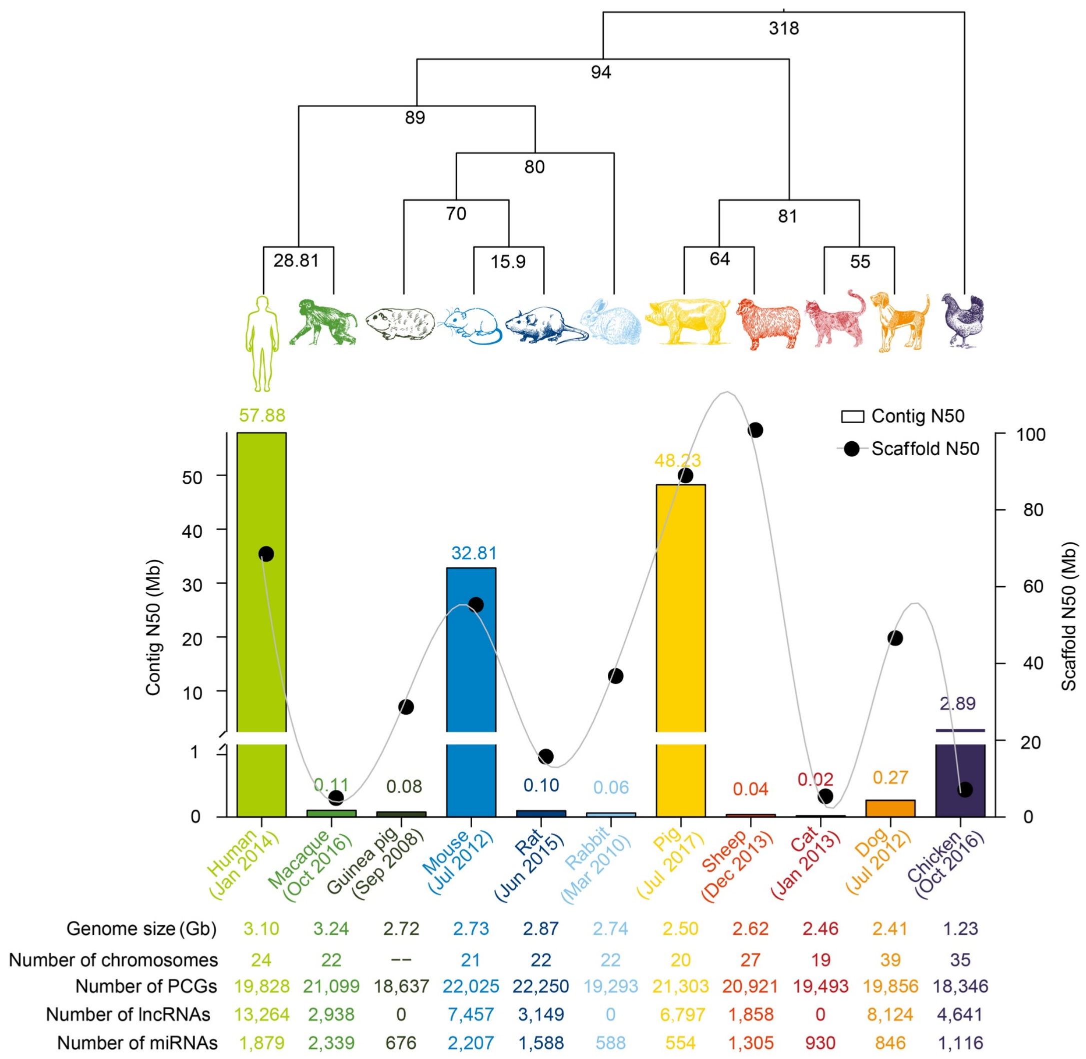

Supplementary Fig. 1. Comparison of size, chromosome number, and transcribed features of reference genomes of pig, human, and other common animal models. Top panel: The divergence times (numbers on nodes; million years ago [MYA]) and the phylogenetic topology of 11 animals were retrieved from the TimeTree database (http://www.timetree.org/). Middle panel: N50 values of each genome assembly were calculated using fragments longer than 500 bp. The publication dates of reference genome assemblies are shown in parentheses, *i.e.*, human (GRCh38.p10), pig (Sscrofa11.1), macaque (Mmul_8.0.1), rabbit (OryCun2.0), mouse (GRCm38.p5), rat (Rnor_6.0), guinea pig (Cavpor3.0), sheep (Oar_v3.1), dog (CanFam3.1), cat (Felis_catus_6.2) and chicken (Gallus_gallus-5.0). All annotation information was obtained from Ensembl reference genomes.

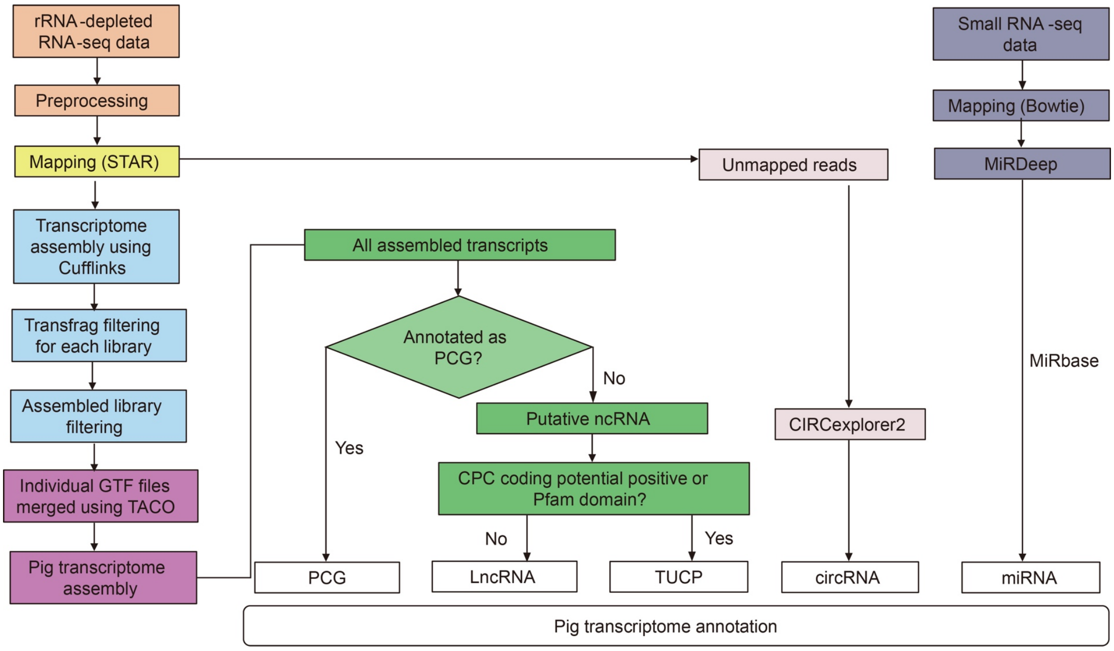

Supplementary Fig. 2. Workflow for reconstruction of the pig transcriptome. Sequence reads were aligned to the pig reference genome (Sscrofa 11.1, GCA_000003025.6) by the STAR alignment tool (version 2.5.3a). The aligned reads of these samples were assembled using Cufflinks (version 2.1.1). We then filtered out library-specific background noise and predicted the most likely isoforms from the assemblies of transcript fragments (transfrags) (**see more details in Supplementary Methods**). After filtering, the remaining high-quality transcript assemblies were then subjected to TACO (version 0.7.3), which led to construction of transcriptome maps. We then assessed the coding potential of putative non-coding transcripts that were not annotated as PCGs in the pig reference genome by integrating two sources of evidence: coding potential calculator and detected Pfam A domain matches. Transcripts without coding potential (CPC score <0 and without Pfam domain hits) were defined as lncRNAs, otherwise TUCPs.

Unmapped reads from the STAR mapping were retrieved and used for circRNA prediction using the CIRCExplorer2(version 2.3.2).

The small RNA-seq data were mapped to the reference genome using Bowtie. Mappable reads were submitted to miRDeep (version 2.0.0.7) to detect miRNAs, while annotated mature miRNA sequences of pig and all other mammalian and avian species in miRbase (release 22) were selected as references.

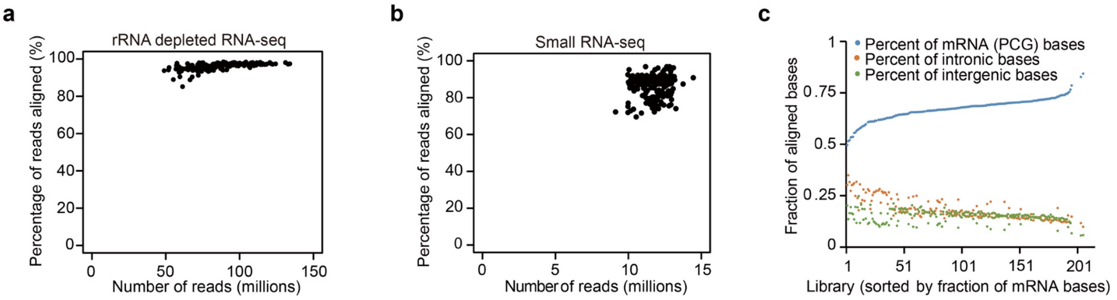

Supplementary Fig. 3. Mapping of rRNA-depleted RNA-seq data and small RNA-seq data. a, b, All rRNA-depleted RNA-seq data **(a)** and small RNA-seq data **(b)** showed a high mapping ratio (~96.13% for RNA-seq data and ~85.92% for small RNA-seq data), which is not limited to the sequenced number of reads. Each dot represents a library. **c,** Analysis of the fraction of aligned bases corresponding to different genomic locations in rRNA-depleted RNA-seq data. Dot plot shows the fraction of aligned bases (y-axis) corresponding to RefSeq mRNAs (blue points), intronic regions (orange points), or intergenic regions (green points) for each library (x-axis). A total of 193 of 194 libraries had greater than 50% of aligned bases correspond to RefSeq mRNAs, indicating their high quality. The libraries were ranked from low to high according to the fraction of aligned bases corresponding to RefSeq mRNAs (blue points).

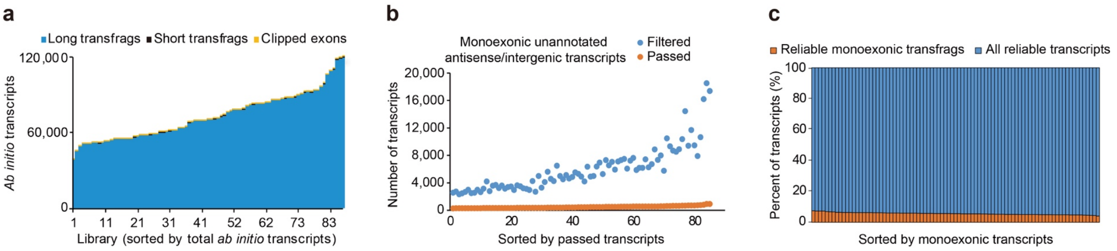

Supplementary Fig. 4. Quality assessment of the long transcript assemblies. a, The numbers of short transfrags (black), short clipped exons (yellow), and long transfrags (blue) for each library. Transcripts with very short first or last exons (<15 bp) were clipped. Short transfrags with lengths ≤250 bp were discarded as poorly assembled transcripts. Long transfrags were retained as high-quality transfrags, which accounted for most (~98.78%) of the *ab initio* transcripts. See **Methods** for details. **b,** Stringent filtering step of unannotated monoexonic transfrags. Approximately 5,983 unannotated monoexonic transfrags (ranging from 2,332 to 18,501) in individual libraries were subjected to bivariate kernel density classifier, ~5,533 transfrags (ranging from 2,059 to 17,600) were filtered from each library, and ~450 transfrags (ranging from 263 to 914) were retained. **c,** All retained, reliable monoexonic transfrags in each library. Among the final set of reliable transcripts in each library (~30,112 transcripts, ranging from 25,492 to 34,933), the monoexonic transcripts accounted for ~6% (ranging from 4.23% to 8.09%), which was comparable to the previously reported percentage for the human transcriptome (~5%)[1].

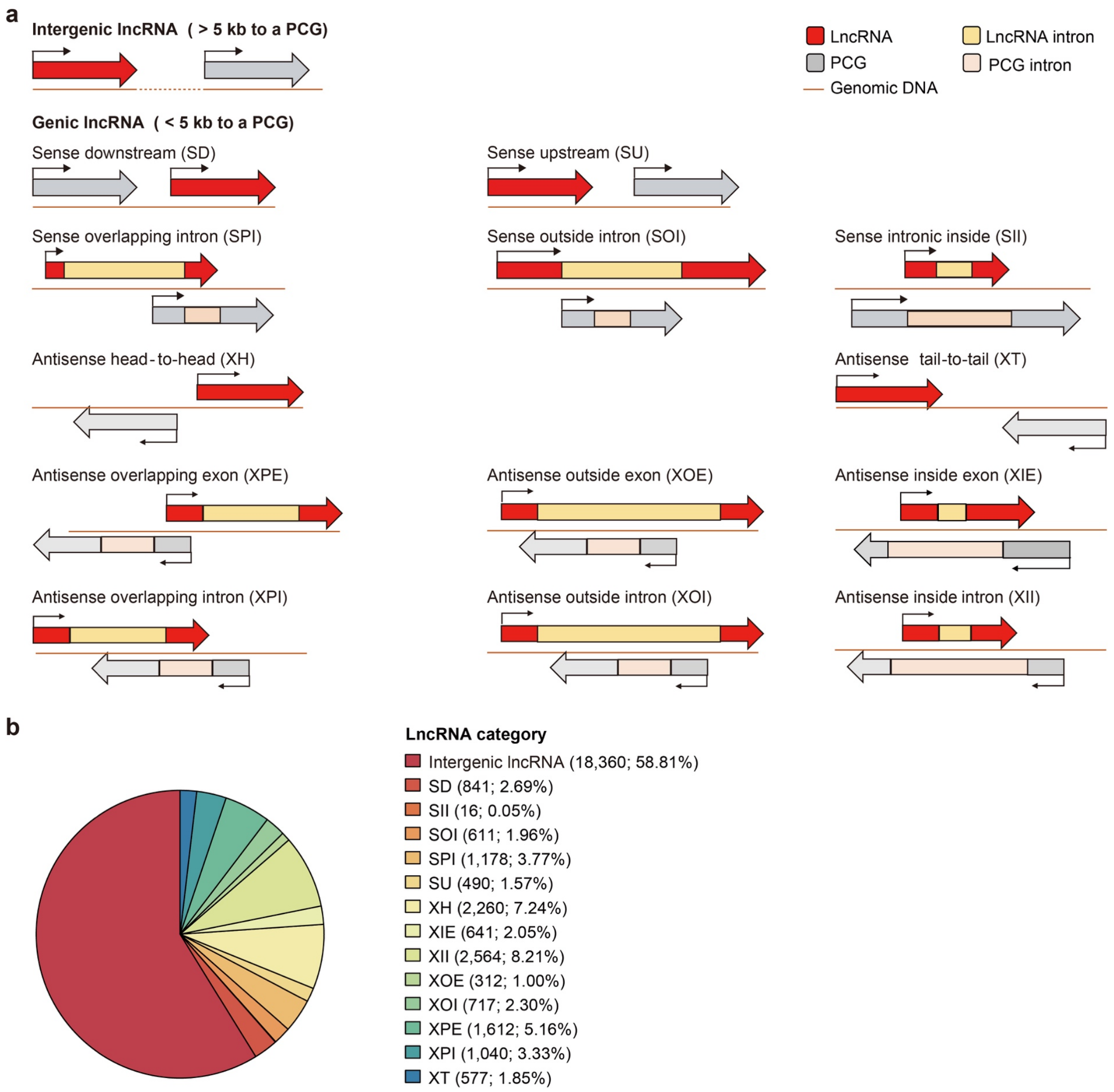

Supplementary Fig. 5. Classification of lncRNA biotypes based on their location with respect to PCGs. a, Illustration of the positional relationships of lncRNAs and PCGs for each lncRNA category. LncRNAs were divided into 14 different locus biotypes. **b,** Number and percentage (indicated in parentheses) of lncRNAs in each category. Notably, the intergenic lncRNAs constituted more than half (58.81%) of the lncRNAs.

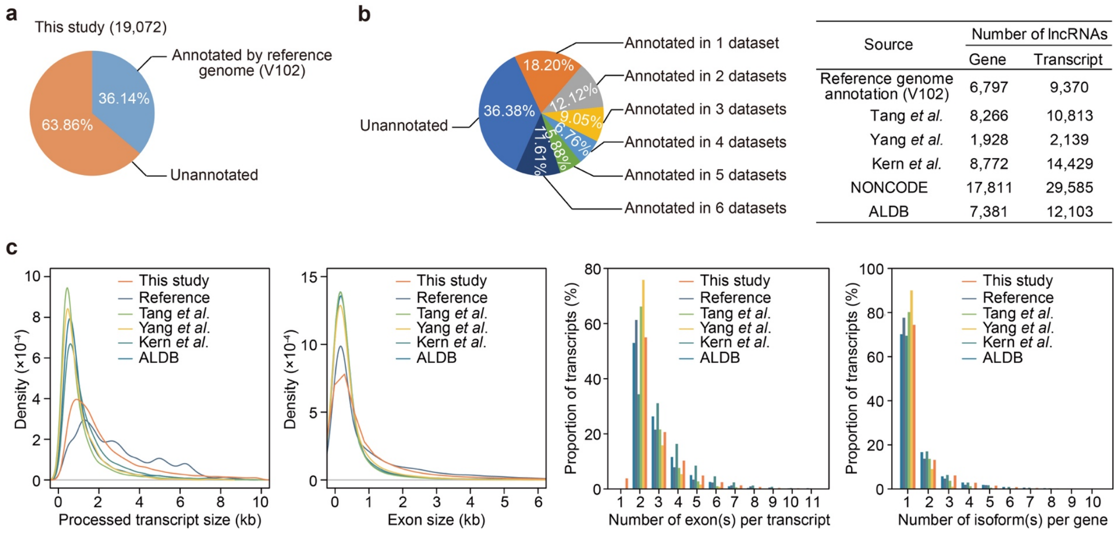

Source	Number of lncRNAs	
	Gene	Transcript
Reference genome annotation (V102)	6,797	9,370
Tang *et al.*	8,266	10,813
Yang *et al.*	1,928	2,139
Kern *et al.*	8,772	14,429
NONCODE	17,811	29,585
ALDB	7,381	12,103

Supplementary Fig. 6. Comparative analysis between lncRNAs in this study and other catalogs. a, Pie charts of the number of lncRNA genes that are unannotated or annotated in pig reference genome (release v102). **b,** Pie chart (left) showing the proportion of lncRNAs in this study that were previously discovered by only one or multiple (from 2 to 6) datasets listed in the table (right). About 36.38% were not discovered by any of the catalogs. Basic Local Alignment Search Tool (BLASTN) was used to discriminate the previously discovered lncRNAs in pig reference genome (v102)[2], Tang *et al.* (2017)[3], Yang *et al.* (2017)[4], Kern *et al.* (2018)[5] and ALDB[6], NONCODE database[7] using the following criteria: e-value < 1×10^{-10}, min-identity of 80%, and min-coverage of 80%. **c,** Features (transcript length, lengths of exons, number of exons per transcript, and number of isoforms per gene) reported in different lncRNA catalogs.

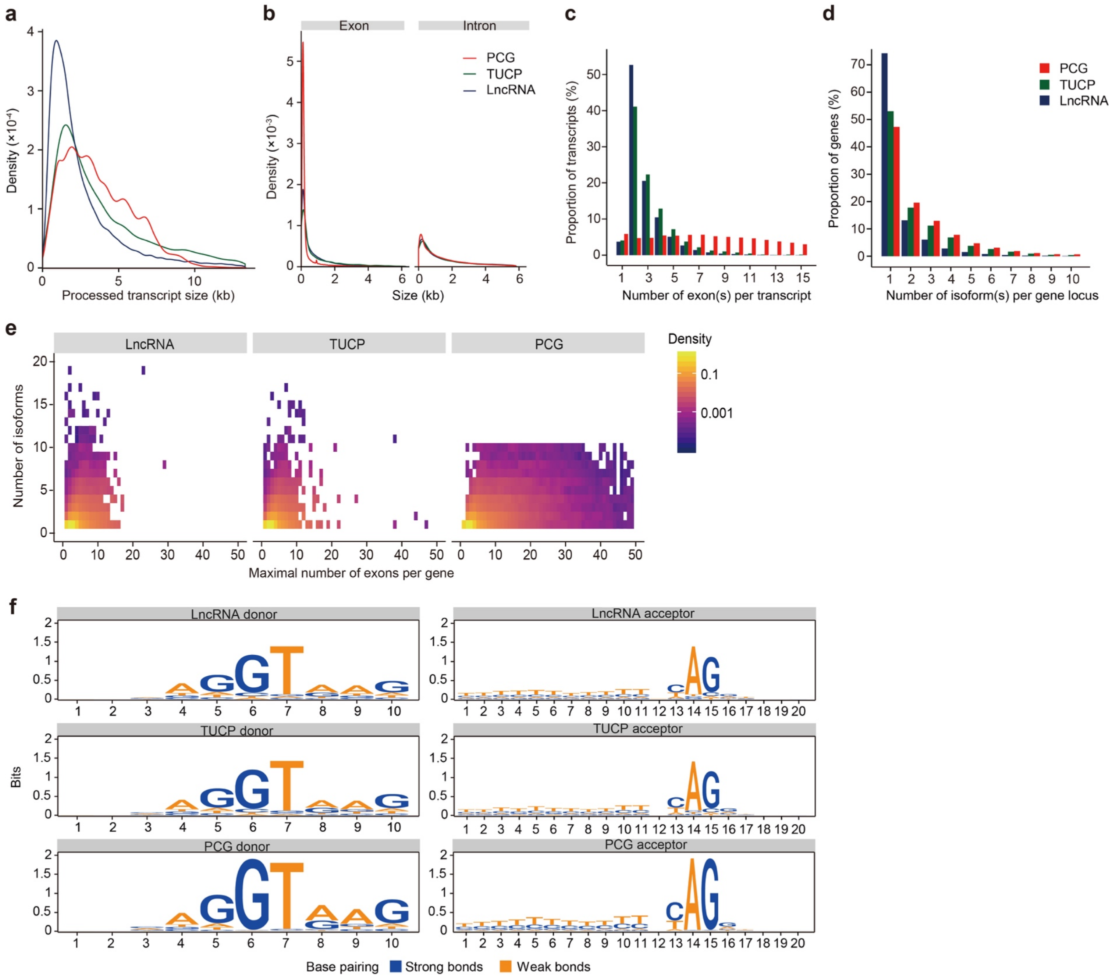

Supplementary Fig. 7. Features of three long transcript types (*i.e.,* PCGs, TUCPs, and lncRNAs). Transcript length (**a**), lengths of exons and introns (**b**), number of exons per transcript (**c**), number of isoforms per transcript (**d**), the number of isoforms and maximum number of exons per transcript (**e**), and splicing junction sequences at donor and acceptor sites (**f**). Notably, lncRNAs have fewer exons and isoforms but analogous canonical splicing junction sequences ('GT' donor and 'AG' acceptor) compared to those of PCGs, as shown in (**c**), (**d**) and (**f**). Color bar in (**e**) denotes the log_{10}-transformed two-dimensional kernel density.

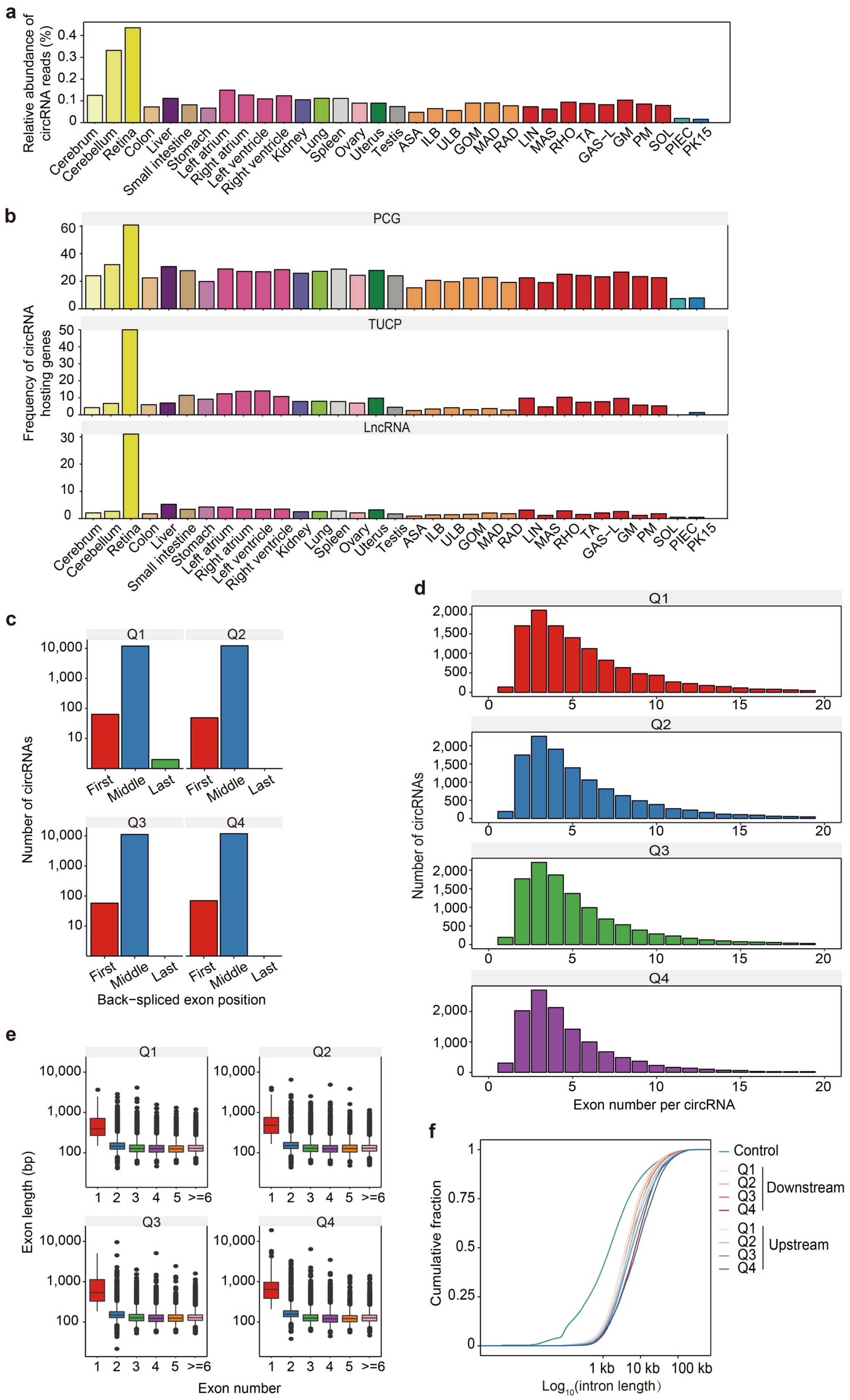
a
Relative abundance of circRNA reads (%)
b
PCG
TUCP
LncRNA
Frequency of circRNA hosting genes
Cerebrum
Cerebellum
Retina
Colon
Liver
Small intestine
Stomach
Left atrium
Right atrium
Left ventricle
Right ventricle
Kidney
Lung
Spleen
Ovary
Uterus
Testis
ASA
ILB
ULB
GOM
MAD
RAD
LIN
MAS
RHO
TA
GAS-L
GM
PM
SOL
PIEC
PK15
c
Number of circRNAs
First
Middle
Last
Back−spliced exon position
d
Number of circRNAs
Exon number per circRNA
e
Exon length (bp)
Exon number
f
Cumulative fraction
Control
Downstream
Upstream
Log10(intron length)

Supplementary Fig. 8. Features of circRNAs. a, Relative abundance of circRNA reads. The percentage of circular junction reads from all reads mapped to the reference genome is shown for different tissues. The neural tissues (especially retina and cerebellum) have prominently more circRNA reads than other tissues, while the two cell lines have the fewest circRNA reads. **b,** The proportion of genes that harbor circRNAs. The retina has the most circRNA-harboring genes, which is consistent with the retina having the highest number of circRNA reads, indicating a more extensive role of circRNAs in the retina than in other tissues. **c,** Distribution of circRNA back-spliced exon positions along the linear PCGs that harbor them, with more circRNAs produced from the middle exons of transcripts. This result is presented in four quartiles of expression levels from Q1 to Q4 (each containing an equal number of circRNAs) which indicate TPM values from low to high. Q1: 0.05-0.06 TPM, Q2: 0.06-0.083 TPM, Q3: 0.083-0.15 TPM, and Q4: >0.15 TPM. **d,** Distribution of the number of exons per circRNA in four expression intervals. **e,** Distribution of exon lengths in circRNAs with different exon numbers. The length of back-spliced exons was greater in single-exon circRNAs (Q1 n = 139; Q2 n = 195; Q3 n = 196; Q4 n = 304) than in multiple-exon circRNAs (2 exons: Q1 n = 1,708; Q2 n = 1,748; Q3 n = 1,767; Q4 n = 2,025. 3 exons: Q1 n = 2,108; Q2 n = 2,262; Q3 n = 2,208; Q4 n = 2,703. 4 exons: Q1 n = 1,707; Q2 n = 1,907; Q3 n = 1,870; Q4 n = 2,129. 5 exons: Q1 n = 1,402; Q2 n = 1,396; Q3 n = 1,372; Q4 n = 1,420. ≥ 6 exons: Q1 n = 4,948; Q2 n = 4,770; Q3 n = 3,915; Q4 n = 3,452). In the boxplot, the internal line indicates the median, the box limits indicate the upper and lower quartiles and the whiskers extend to 1.5 IQR from the quartiles. **f,** Length distribution of flanking introns (up- and downstream) of circularized exons. The flanking introns showed significantly greater lengths (~5,759 bp and ~4,992 bp for upstream and downstream introns, respectively) than control introns (~1,592 bp). Highly abundant circRNAs often have longer flanking introns than less abundant circRNAs.

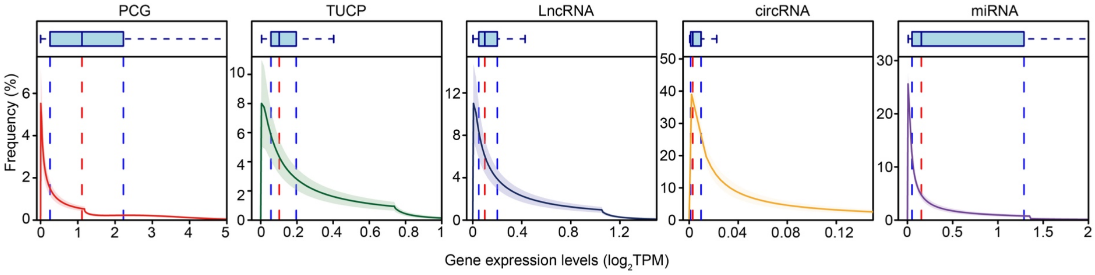

Supplementary Fig. 9. Expression distribution of five types of transcripts. The x-axis indicates the transcripts sorted from lowest to highest abundance. Colored lines represent mean values across tissues, and lighter-colored shading around the mean represents dispersion, calculated as the standard deviation. Data are presented as mean values ± SD (n = 33). The red vertical dashed lines indicate median expression level. The left and right blue vertical dashed lines indicated 25th and 75th quantiles, respectively. The upper box plot also shows the expression distribution (PCG n = 20,504; TUCP n = 2,440; LncRNA n = 19,072; circRNA n = 48,232; miRNA n = 1,245). In the boxplot, the internal line indicates the median, the box limits indicate the upper and lower quartiles and the whiskers extend to 1.5 IQR from the quartiles.

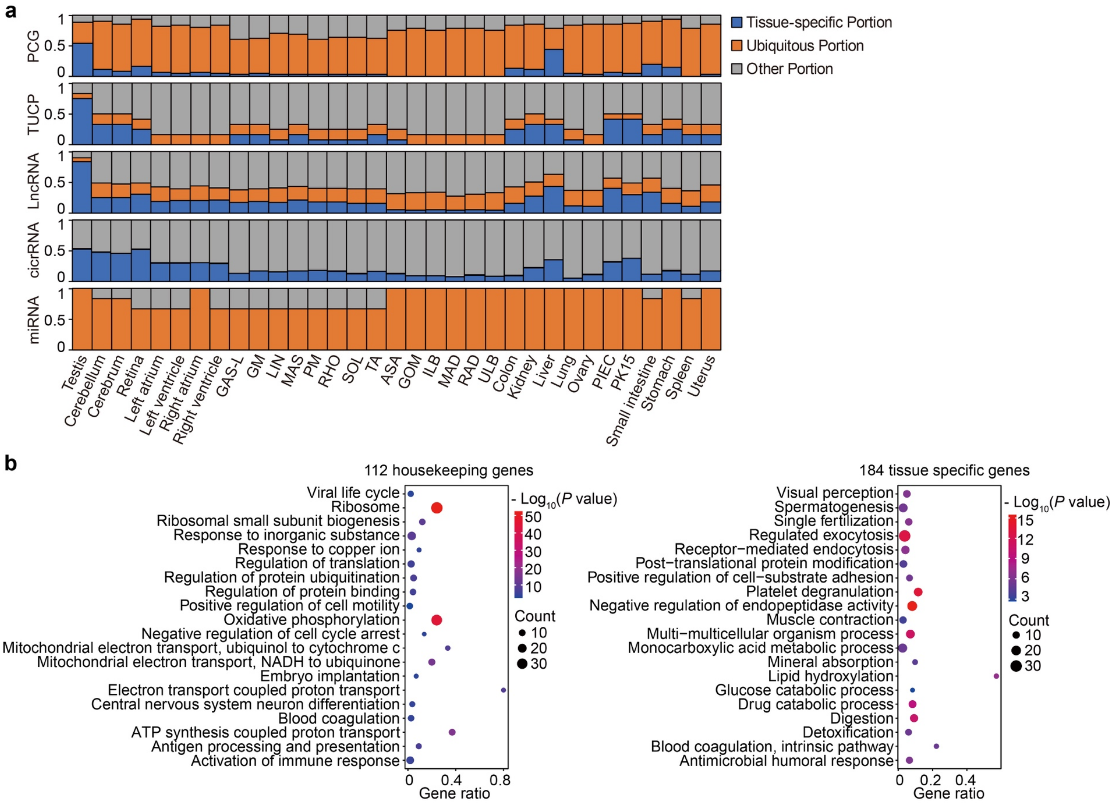

Supplementary Fig. 10. Characterization of the top 0.5% of highly abundant transcripts. a, Tissue-specific ($\tau \geq 0.75$) (blue) and ubiquitous ($\tau \leq 0.3$) (orange) transcripts among the top 0.5% highest abundance transcripts across tissues. Many of the highly transcribed PCGs (~69.96%) showed ubiquitous/housekeeping transcription patterns

across tissues. Many (~85.86%) of the highly transcribed miRNAs were housekeeping miRNAs. For TUCPs, lncRNAs, and circRNAs, the top 0.5% most highly transcribed genes included only a small proportion of housekeeping genes (15.66%, 22.74%, and 0.41%, respectively) but had greater numbers of tissue-specific genes (16.16%, 20.29%, and 21.82%, respectively) compared to PCGs (~8.11% were tissue-specific). **b,** Functional enrichment of tissue-specific and ubiquitously transcribed PCGs within the top 0.5% most abundant PCGs. The sizes of dots represent numbers of enriched genes, and dot color represents the -log$_{10}$(*P*-value) (unadjusted). *P* values are calculated based on a one-sided accumulative hypergeometric test.

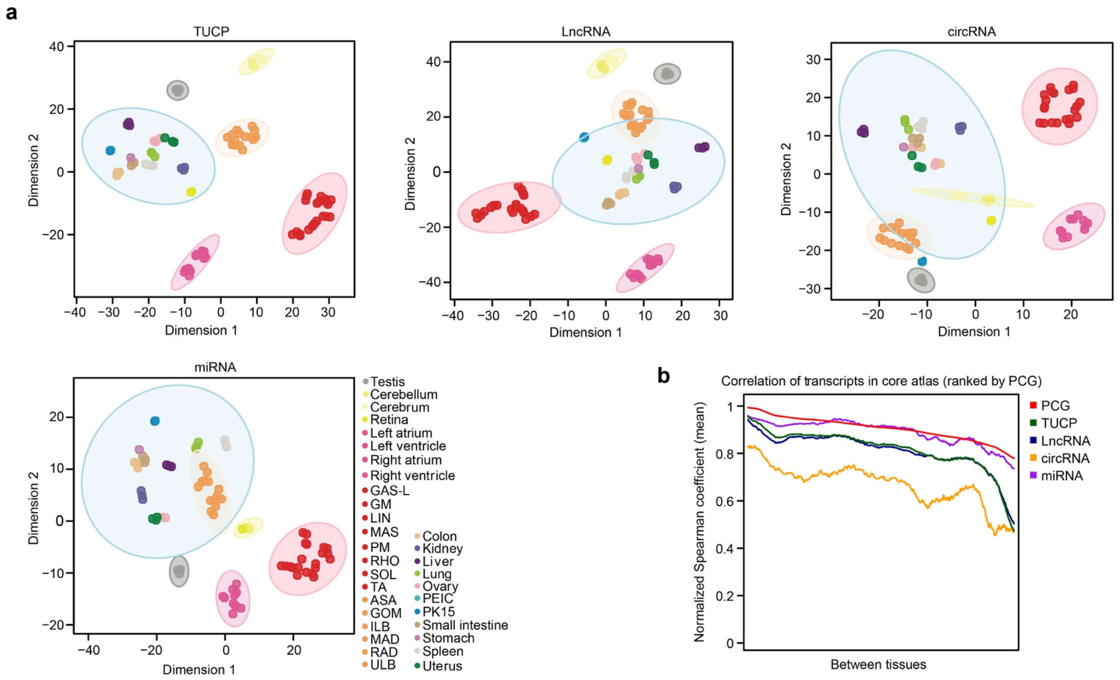

Supplementary Fig. 11. Transcriptional profiling of distinct transcript types across tissues. a, Sample and tissue similarity of TUCP, lncRNA, circRNA, and miRNA transcriptional profiles. Comparison of transcription profile similarity using multidimensional scaling based on distances through t-distributed stochastic neighbor embedding (t-SNE) revealed a characteristic transcriptional pattern across tissues. Ellipses indicate the tissue groups that have similar functions, constructed at a probability of 0.95. **b,** Pairwise Spearman correlations between tissues based on transcriptional profiling. The coefficient was normalized across pairwise comparisons within transcript type. The pairwise comparisons indicated on the x-axis were sorted by the coefficient from low to high. The curve plot was smoothed using moving average smoothing.

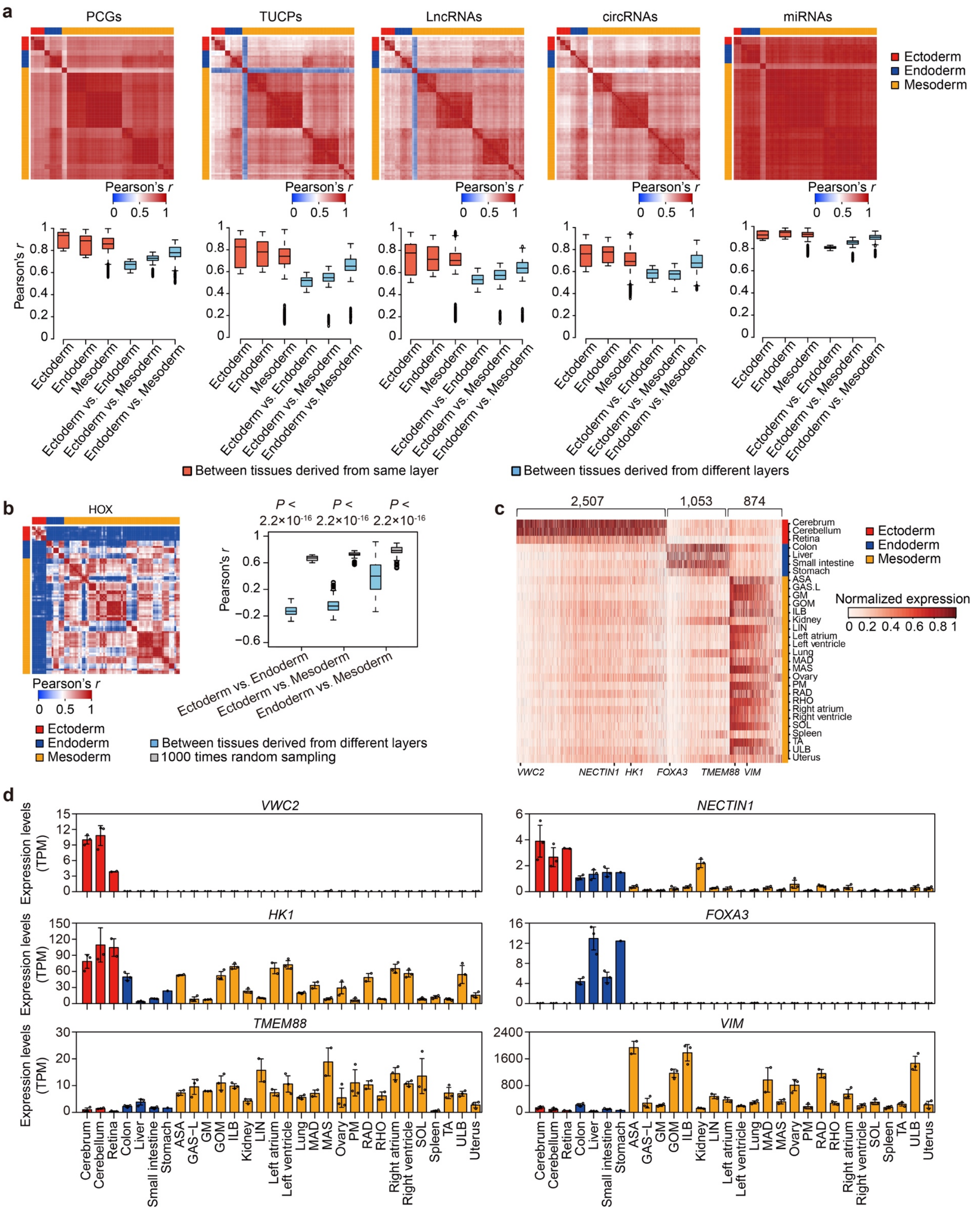

Supplementary Fig. 12. Expression profiling of tissues derived from three germ layers. a, Heatmap (top) and boxplot (bottom) comparisons of transcriptional profiles for each of the five types of transcripts in tissues derived from distinct germ layers using Pearson's correlation. In the boxplot, the internal line indicates the median, the box limits indicate the upper and lower quartiles and the whiskers extend to 1.5 IQR from the quartiles. All 31 tissues were partitioned into 3 distinct germ layer-related groups: 3 ectoderm-derived tissues (cerebrum, cerebellum, and retina), 4 endoderm-derived tissues (colon, intestine, liver, and stomach), and 24 mostly mesoderm-derived tissues (*e.g.,* heart, lung, and uterus *etc.*), listed in **Supplementary Data 1**, as described in Hon *et al*[8]. **b,** Transcriptional

patterns of *HOX* genes suggesting the developmental origins of these tissues by germ layer. A randomly selected equal number of PCGs were used as the control (grey box) and showed higher coefficients than *HOX* genes (two-sided Wilcoxon rank-sum test) (ectoderm *vs.* endoderm n = 80, $P < 2.2 \times 10^{-16}$; ectoderm *vs.* mesoderm, n = 520, $P < 2.2 \times 10^{-16}$; endoderm *vs.* mesoderm, n = 650, $P < 2.2 \times 10^{-16}$). In the boxplot, the internal line indicates the median, the box limits indicate the upper and lower quartiles and the whiskers extend to 1.5 IQR from the quartiles. **c,** Heatmap with the expression of identified maker genes for the definitive germ layers (2,507 for ectoderm, 1,053 for endoderm, and 874 for mesoderm). Each gene's normalized log expression levels are standardized so that they range between 0 and 1. **d,** Bar plot showing the expression patterns of 6 marker genes from **(c)**. Data are presented as mean values ± SD.

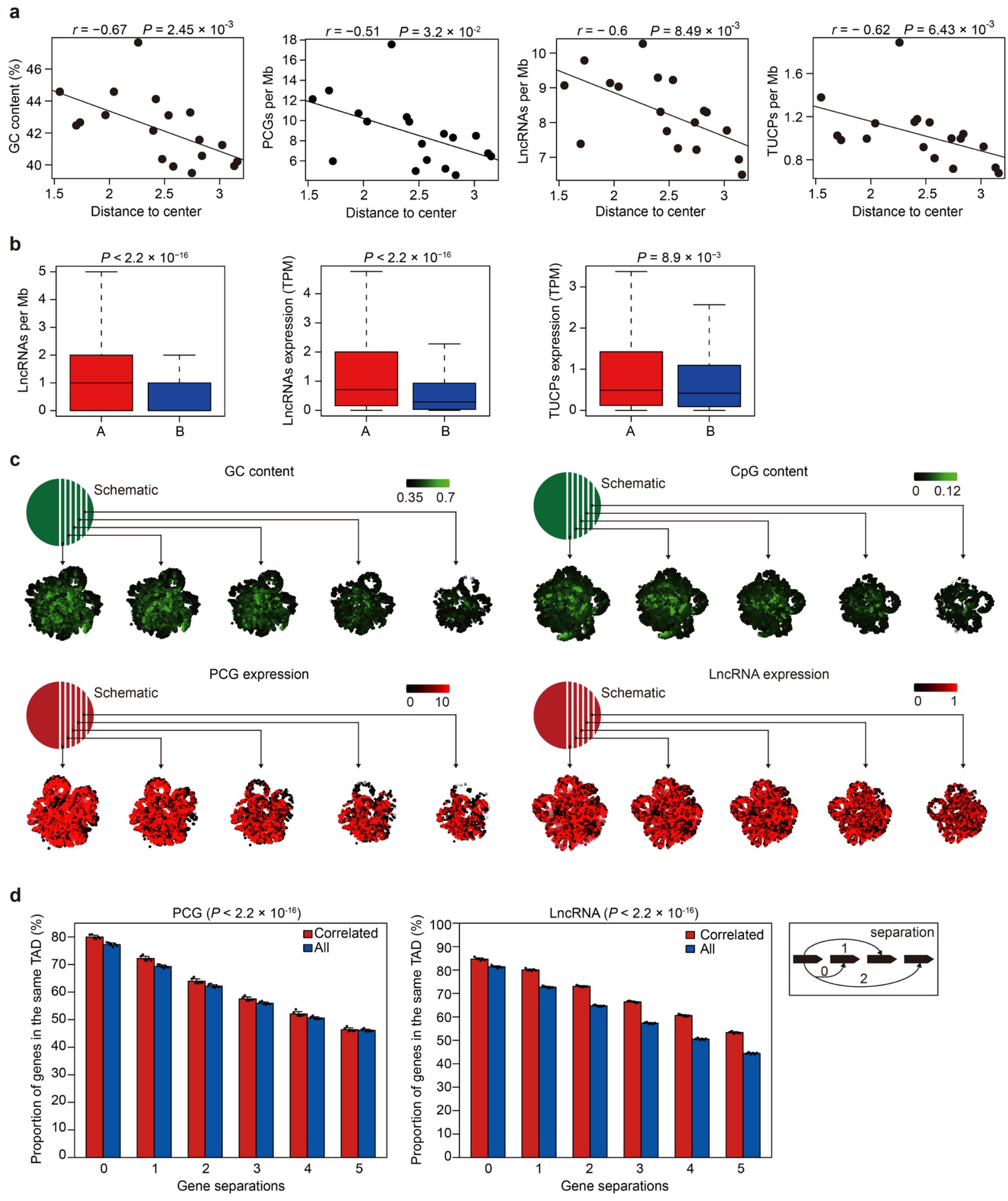

Supplementary Fig. 13. 3D genome landscape of chromatin organization. a, Preferential localization of chromosomes within the nuclear space. Scatter plot indicating Spearman's correlations between average chromosome distances to the nuclear center and their sequence features, including GC content, PCG density, lncRNA density, and TUCP density. The statistical significance of the two-sided *P* value was calculated using hypothesis testing. **b,** Comparison of transcription density and expression level for lncRNAs and TUCPs between compartment A and compartment B. Compartment A (1,105 Mb in length) is GC-rich [43.71%], transcript-rich (9.85 lncRNAs per Mb and 1.35 TUCPs per Mb) and actively transcribed (0.71 TPM for lncRNAs and 0.49 TPM for TUCPs). Compartment B (1,161 Mb in length) is GC-poor [38.26%], transcript-sparse (6.20 lncRNAs

per Mb and 0.62 TUCPs per Mb) and not actively transcribed (0.29 TPM for lncRNAs and 0.42 TPM for TUCPs). (Two-sided *P*-value from Wilcoxon test, $P < 2.2 \times 10^{-16}$, $P < 2.2 \times 10^{-16}$, $P < 8.9 \times 10^{-3}$). **c,** 3D models of the pig genome in subcutaneous AT (ULB). The plot is presented in quintuplicate, with five intersecting sections plotted from the interior regions of the nucleus (left) to the periphery (right) based on distance (schematically depicted in the left inset). The properties of GC content, CpG content, PCG expression, and lncRNA expression were aggregated in 3D space. The color bar (right) indicates values for each property. TUCP expression is not shown, as transcription was relatively rare. **d,** Co-expression patterns of transcripts within the same TAD. Representative bar plots of the percentage of correlated gene pairs ($r > 0.5$) (red bars) based on expression within the same TADs (at a 20 kb resolution) compared to all gene pairs (blue bars). Tested gene pairs are stratified based on the number of separating genes between them, as schematically depicted (upper-right inset). The distance is indicated underneath the bar plot. Fisher's method was used to combine the *P* values of the one-sided binomial tests that were performed for each gene-pair distance (PCGs: $P < 2.2 \times 10^{-16}$, lncRNAs: $P < 2.2 \times 10^{-16}$). The pattern for TUCPs is not shown, as their gene density was low (~only 1 per Mb, not applicable for this analysis). Data are presented as mean values ± SD ($n = 6$).

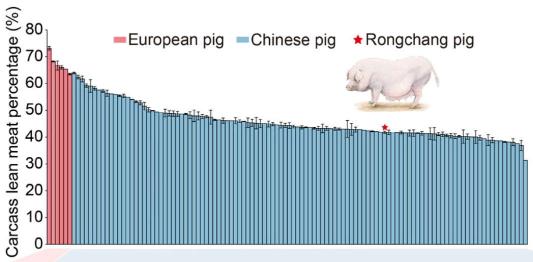

	European pig (6)	Chinese pig (110)
Pig breed	Pietrain, Duroc, Large White, Landrace, Hampshire, Berkshire	Junmu no.1 white pig, Hubei white pig, Luyan white pig, Sanjiang white pig, Zhejiang middle white pig, Nanchang white pig, Beijing black pig, Songliao black pig, Sutai pig, Yunan black pig, Wuzhishan pig, Hanzhong white pig, Dahe black pig, Shanghai white pig, Yuedong black pig, Lulai black pig, Dianlu pig, Tunchang pig, Bama xiang pig, Nanyang black pig, Shanxi black pig, Minbei spotted pig, Yangxin pig, Congjiang xiang pig, Mingguang small-ear pig, Dingan pig, Mashen pig, Laiwu pig, Xianxi black pig, Zhaotong pig, Ganxi two-end-black pig, Jinhua pig, Min pig, Wanbei pig, Baixi pig, Wannan black pig, Jianbai xiang pig, Huainan pig, Dingyuan pig, Queshan black pig, Huai pig, Liangshan pig, Gaoli gongshan pig, Longlin pig, Lantang pig, Leping pig, Hetao big-ear pig, Huaibei pig, Bamei pig, Lanxi spotted pig, Wuyi black pig, Dapulian pig, Enshi black pig, Daweizi pig, Pudong white pig, Baoshan pig, Diqing tibetan pig, Qiandong spotted pig, Meishan pig, Saba pig, Yacha pig, Ya'nan pig, Zao pig, Shawutou pig, Chenghua pig, Binhu black pig, Guangdong small-ear spotted pig, Hechuan black pig, Large black-white pig, Erhualian pig, Dongchuan pig, Shan pig, Dahe pig, Jiaxing black pig, Ganzhongnan spotted pig, Rongchang pig, Putian pig, Xinhuai pig, Qianbei black pig, Sichuan tibetan pig, Chalu black pig, Shengxian spotted pig, Jiangkou luobo pig, Kele pig, Luchuan pig, Jianli pig, Jiangquhai pig, Dongshan pig, Neijiang pig, Shaziling pig, Tongcheng pig, Qianshao spotted pig, Guanzhuang spotted pig, Diannan small-ear pig, Luopanshan pig, Quxi pig, Guanling pig, Qingping pig, Fengjing pig, Wei pig, Ningxiang pig, Penzhou mountain pig, Yujiang pig, Duntou pig, Hanjiang black pig, Guizhong spotted pig, Yili white pig, Hang pig, Lingao pig, Anqing six-end-white pig

Supplementary Fig. 14. Carcass lean meat percentage for different pig breeds. The percentage of carcass lean meat is an important carcass quality parameter for the pig industry. This parameter is calculated as the percentage of lean muscle versus carcass weight (*i.e.,* the body weight after removing the head, tail, trotter, and visceral tissues except for the kidney), which indirectly reflects the composition of skeletal muscle mass in the whole body. The carcass lean meat percentage ranged from 31.3% to 73.14% across pig breeds (of 116 pig breeds), with 41.8% for Rongchang pig in this study. The data were presented as mean values ± SD based on an averaged of ~13 biological replicates for each pig breed. The table below provides breed names in the order presented in the histogram. All data were collected from *Animal genetic resources in China: pigs*. (ed. China National Commission of Animal Genetic Resources), pp. 18-470. China Agricultural Press, Beijing.

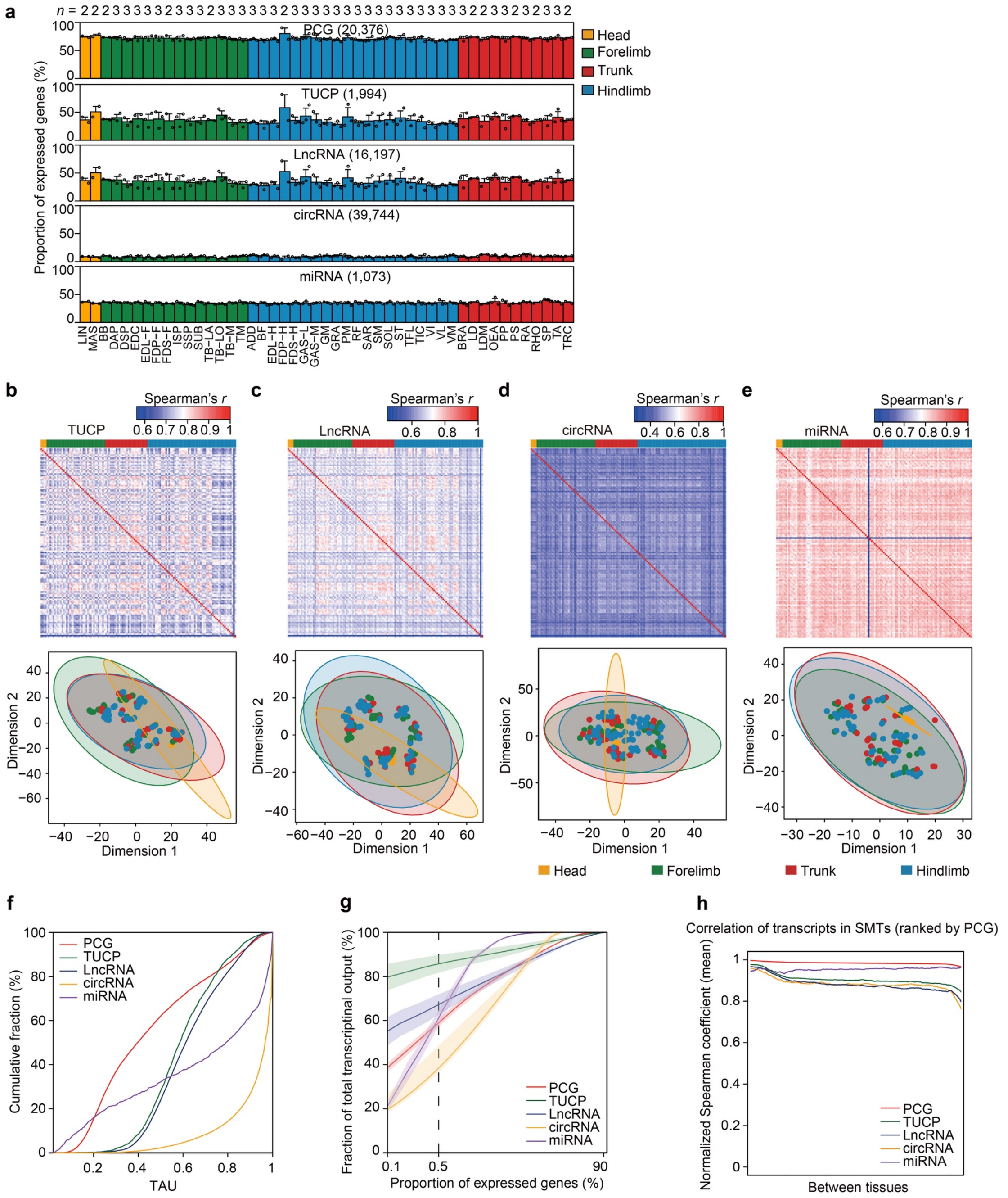

Supplementary Fig. 15. Transcriptional heterogeneity among SMTs. a, Proportions of different transcript types expressed across SMTs from different anatomical regions. The numbers in parentheses indicate total number of transcripts in at least one sample. All transcripts annotated in this study met the expression criteria: 0.1 TPM in at least one sample for PCGs, TUCPs, and lncRNAs and 0.05 and 1 TPM for circRNAs and miRNAs, respectively. Data are presented as mean values ± SD (*n* number is listed above each bar). **b-e,** Similarity of TUCP **(b)**, lncRNA **(c)**, circRNA **(d)** and miRNA **(e)** transcriptional profiles in SMTs from different anatomical regions, as shown for PCGs in **Fig. 3b**. The expression profile similarity based on Spearman correlation and multidimensional scaling analysis of distances through t-SNE revealed a characteristically heterogeneous transcriptional

pattern across SMTs. **f,** Tissue specificity reflected by tau (τ) score for different transcripts across SMTs. **g,** Abundance distribution of distinct transcripts. Cumulative distribution of the average fraction of total transcripts contributed by genes when sorted from most to least abundant in each SMT. The x-axis indicates the proportion of detected genes sorted from most to least transcribed in each SMT, the vertical dashed line indicates the top 0.5% of genes with highest transcription, and the y-axis indicates the accumulated fraction of transcribed genes relative to the total number of genes. Colored lines represent mean values across SMTs, and lighter-colored surfaces around the mean represent dispersion calculated using the standard deviation divided by the cumulative sum of all means. **h,** Pairwise Spearman correlations between SMTs based on transcription level. The coefficient was normalized across pairwise comparisons within transcripts. The curve was smoothed using moving average smoothing. The pairwise comparisons indicated on the x-axis were sorted by the coefficients of PCGs from low to high. Consistent with our core atlas results showing that circRNAs are preferentially expressed in specific tissues, circRNAs are also clearly differentially expressed in SMTs from different anatomical regions, comparable to the patterns observed for lncRNAs.

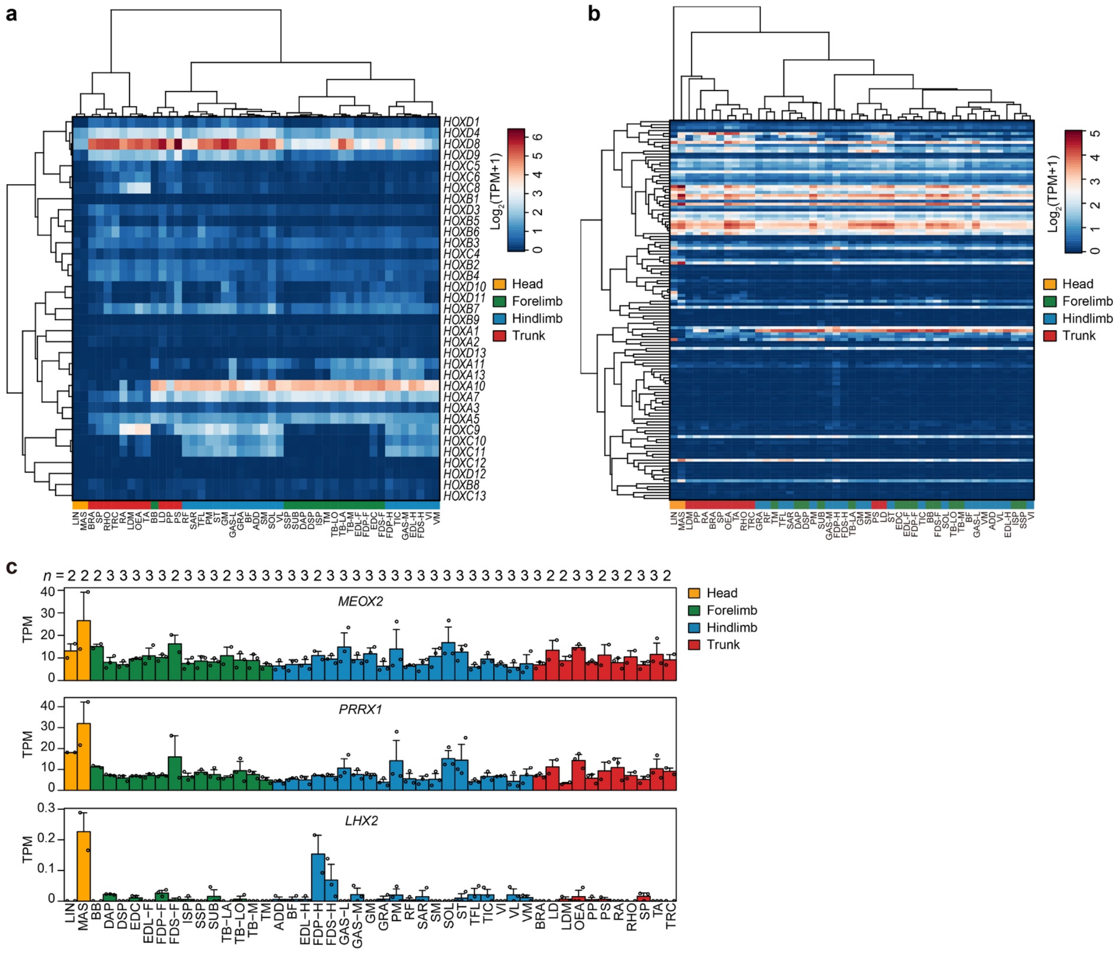

Supplementary Fig. 16. Hierarchical clustering of SMTs in different anatomical regions based on **(a)** *HOX* genes and **(b)** homeobox family genes. Clustering was performed based on $\log_2$-transformed transcription levels. The bottom colored bar indicates the four anatomically distinct regions: head (orange), forelimb (green), trunk (red), and hindlimb (blue). **(c)** Histogram showing the transcription levels across SMTs for representative homeobox family genes, *i.e., MEOX2*, *PRRX1,* and *LHX2*, are highly transcribed in head MAS. Data are presented as mean values ± SD (*n* number is listed above each bar).

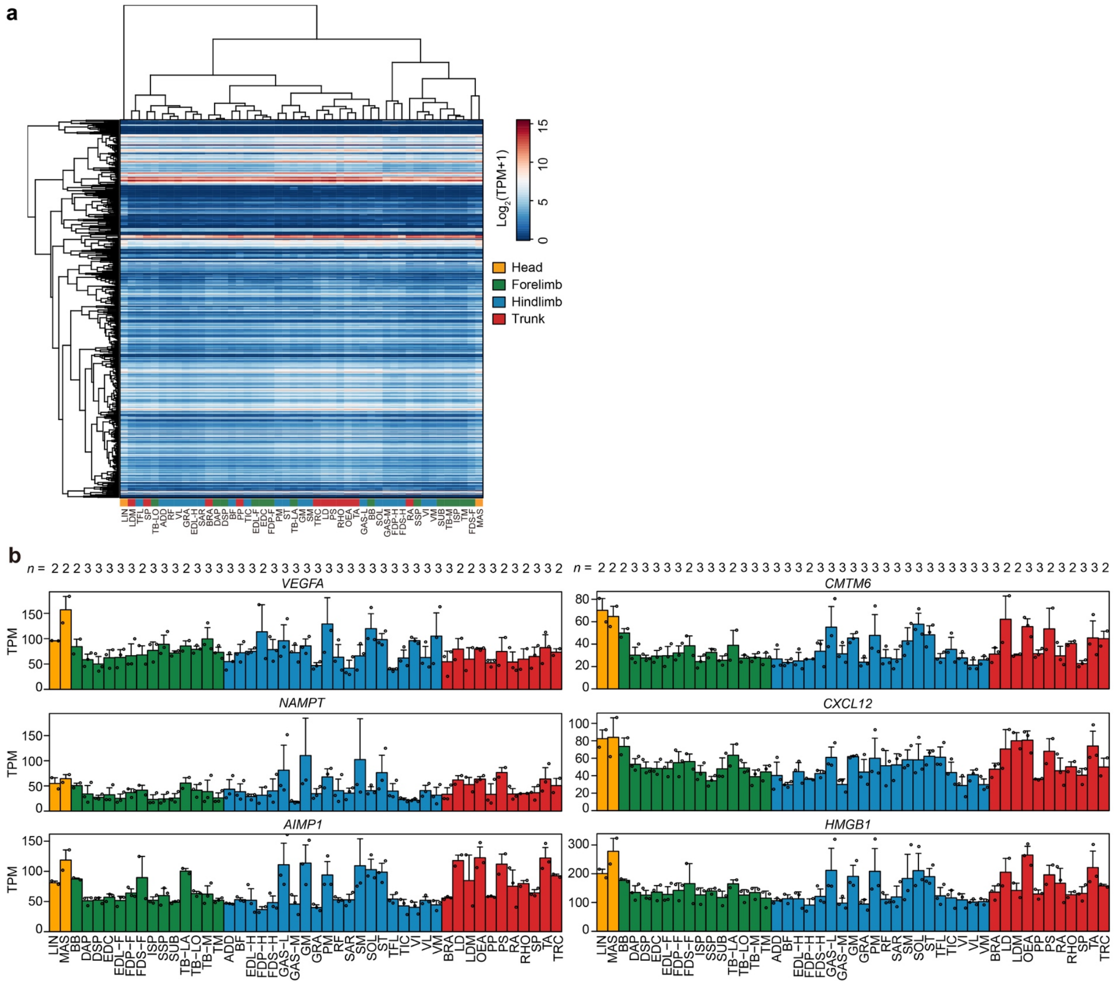

Supplementary Fig. 17. Transcriptional patterns of myokines across SMTs. a, Hierarchical clustering of myokine expression in SMTs from different anatomical regions was performed based on log_2-transformed transcription levels. The bottom colored bar indicates the four anatomically distinct regions: head (orange), forelimb (green), trunk (red) and hindlimb (blue). **b,** Histogram showing the transcription levels of representative highly transcribed myokines. Data are presented as mean values ± SD (*n* number is listed above each bar).

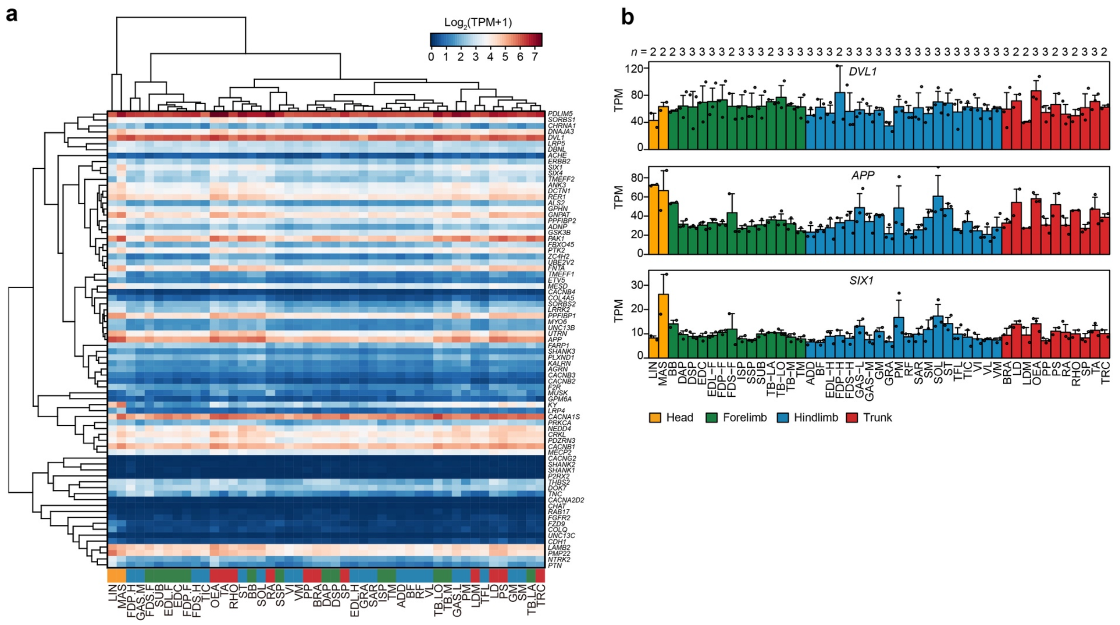

Supplementary Fig. 18. Transcriptional patterns of NMJ-related PCGs across SMTs from different anatomical regions. a, Hierarchical clustering was performed based on $\log_2$-transformed transcription levels. The bottom colored bar indicates the four anatomically distinct regions: head (orange), forelimb (green), trunk (red) and hindlimb (blue). **b,** Histograms showing the transcription levels of representative widely and highly transcribed PCGs (*i.e.*, *DVL1 and APP*) or PCGs specifically transcribed in SMTs from one anatomical group (*i.e.*, *SIX1*). Data are presented as mean values ± SD (*n* number is listed above each bar).

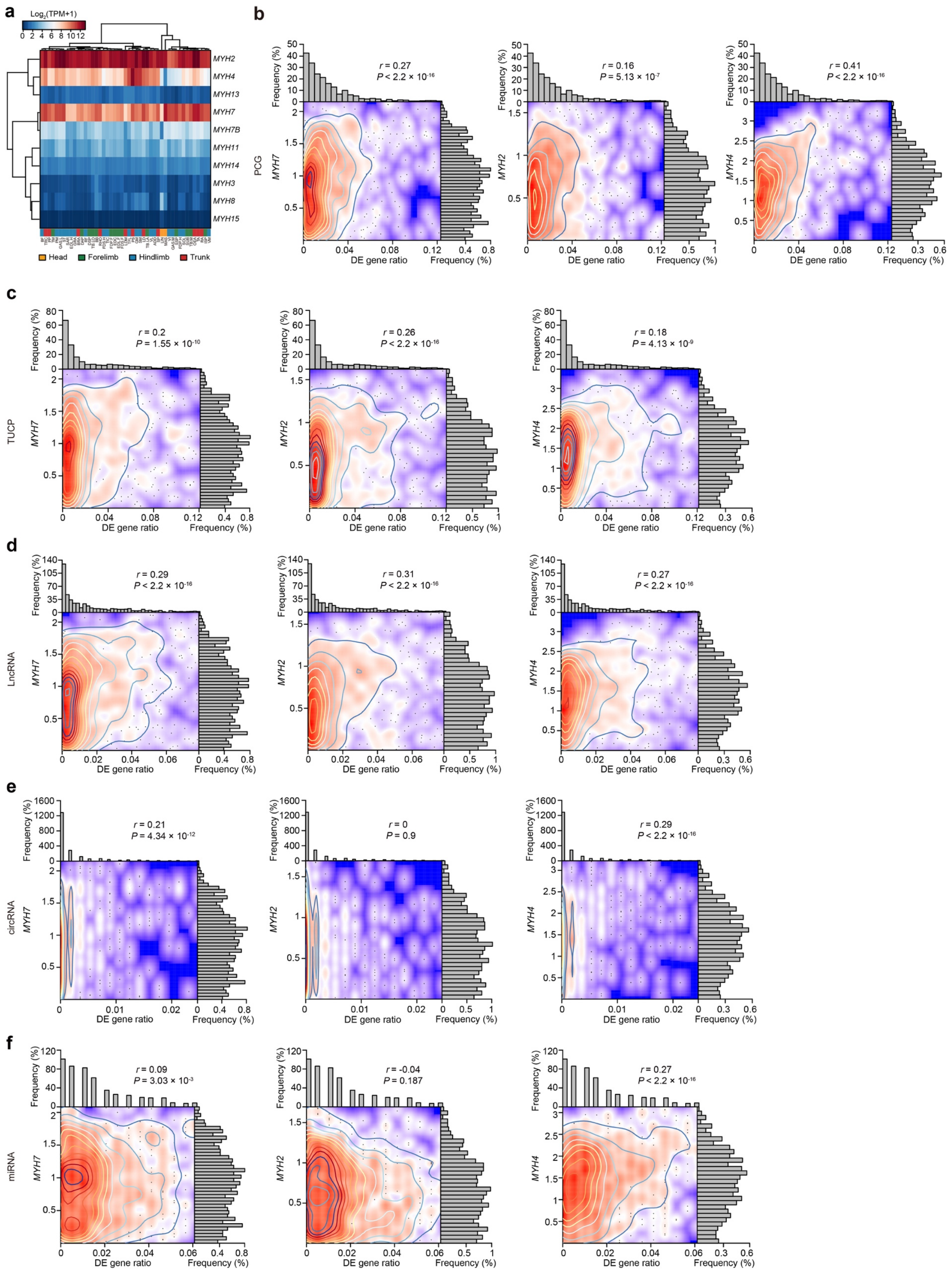

Supplementary Fig. 19. Transcriptional patterns of *MYH*s across SMTs. a, Transcriptional patterns of ten *MYH* isoforms across SMTs. Hierarchical clustering was performed based on log2-transformed transcription levels. The heatmap also indicates the predominant abundance of *MYH7* (TPM=1422.9), *MYH4* (TPM=813.8), and *MYH2* (TPM=4414.5) compared to other *MYH*s (average ~ 6.1 TPM). **b-f,** Density plots showing the correlations between differences in dominant myofiber composition and the transcriptional differences reflected by the differentially expressed (DE) gene ratio,

including **(b)** PCGs, **(c)** TUCPs, **(d)** lncRNAs, **(e)** circRNAs and **(f)** miRNAs. The x- and y-axes indicate the DE gene ratio and the log_2-transformed *MYH* transcription difference of all pairwise SMT comparisons, respectively. Histograms located to the upper and right of the density plots show the distributions of DE gene ratios and *MYH* transcription differences, respectively. The Pearson correlation coefficients and two-sided *P* values are listed in the upper right of each plot. Colors in the density plots indicate high (red) and low (blue) density of dots (pairwise SMT comparisons).

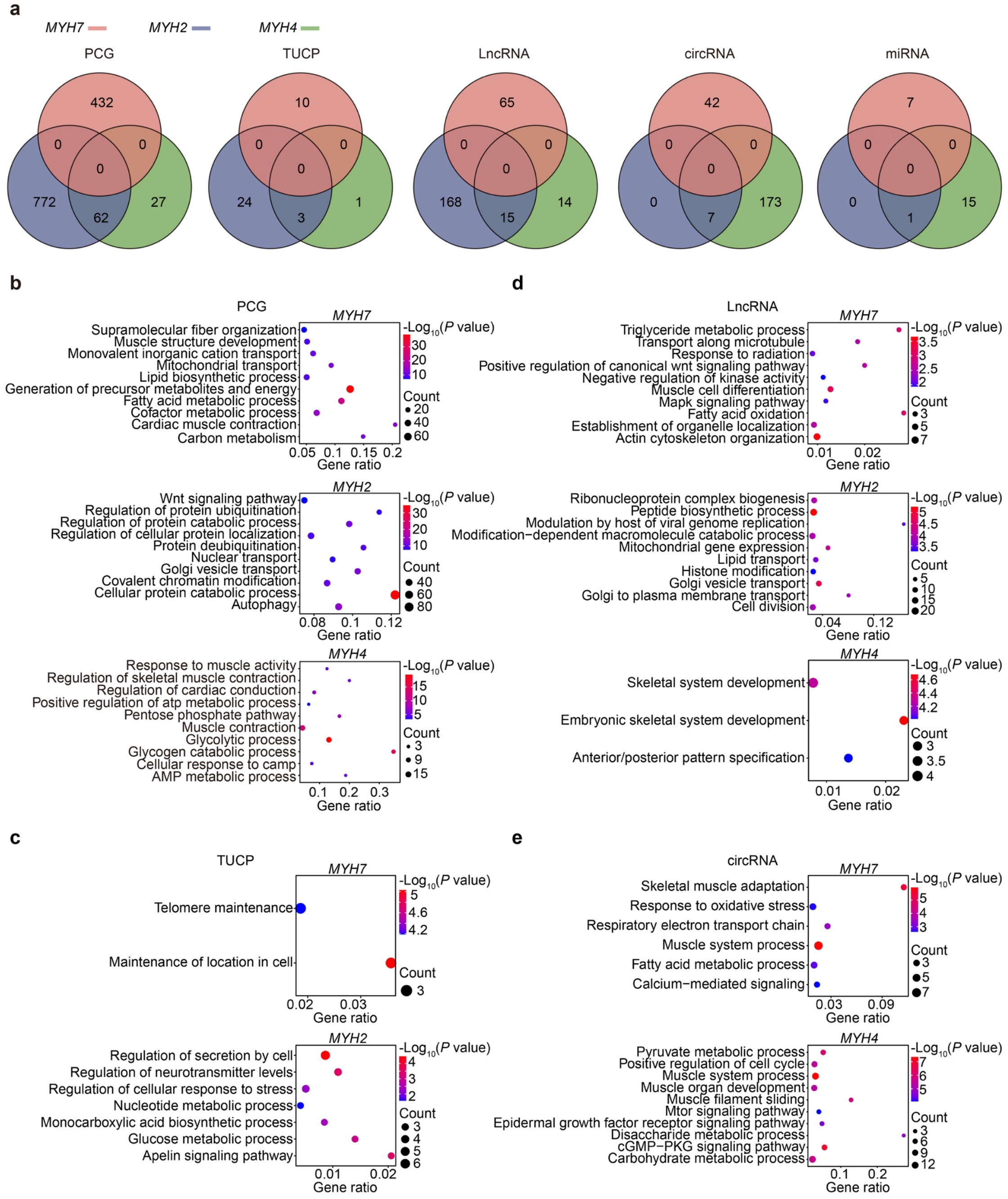

Supplementary Fig. 20. Functions of putative fiber-specific genes. a, Venn diagrams showing overlap of genes that were most significantly (top 10%) correlated with *MYH*

expression. **b-e,** Functional enrichment for PCGs **(b)**, TUCPs **(c)**, lncRNAs **(d)** and circRNAs **(e)** that were specifically correlated with *MYH*s expression. The Y-axis denotes the significantly enriched GO terms. The X-axis indicates the ratio of enriched genes. The sizes of dots represent the numbers of enriched genes, and the color represents the $\log_{10}$(*P*-value) (unadjusted). *P* values are calculated based on a one-sided accumulative hypergeometric test. The functions of TUCPs and lncRNAs specifically correlated with *MYH*s are reflected by the functional enrichment of PCGs in their up- and downstream 100 kb. The functions of circRNAs are reflected by the enrichment for PCGs that harbor circRNAs specifically correlated with *MYH* transcription. The results lacking significantly enriched terms are not shown. Functional annotations of miRNAs specifically correlated with *MYH*s are shown in **Supplementary Data 5**.

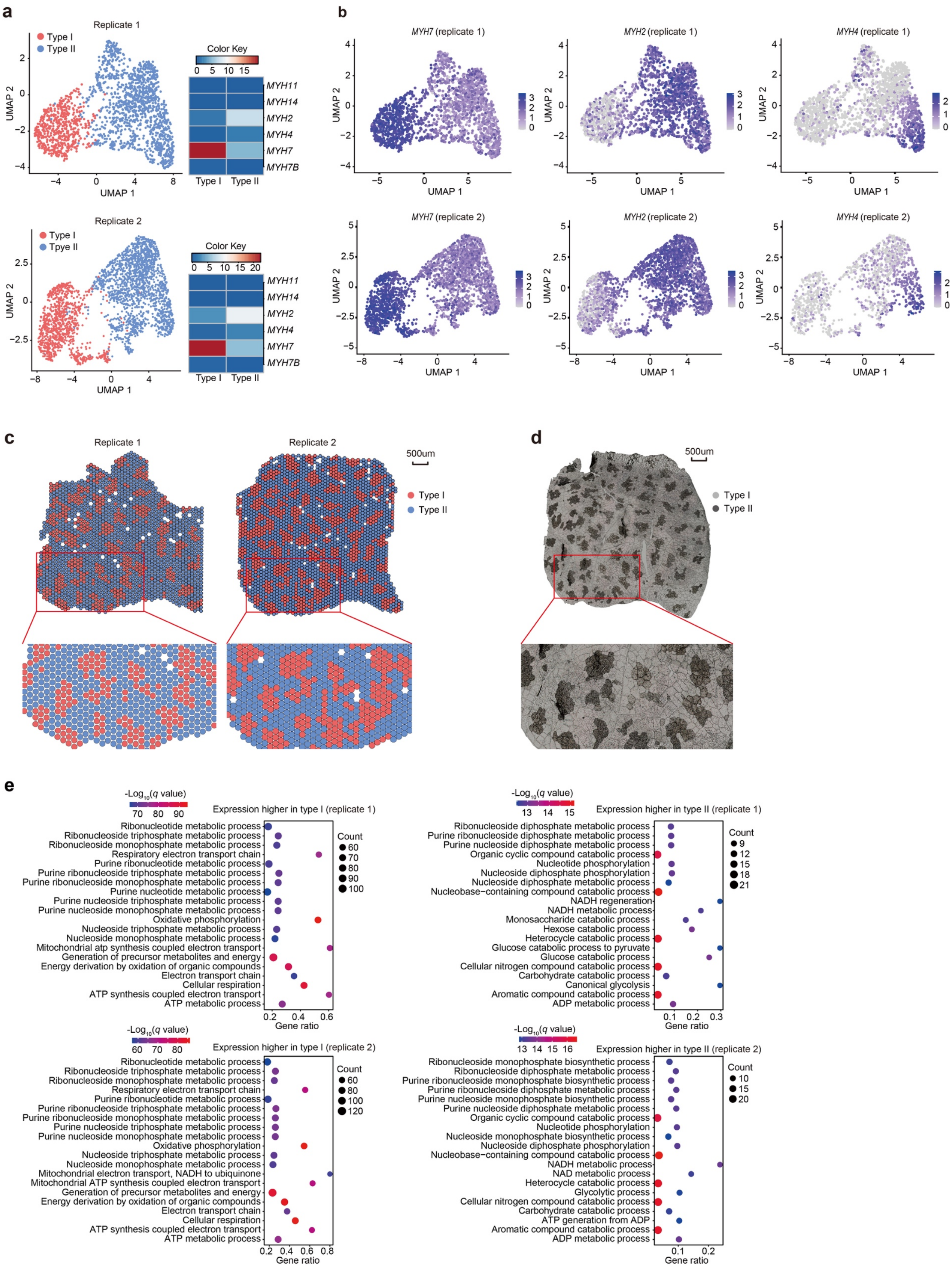

Supplementary Fig. 21. Dimensionality reduction and clustering of spots from psoas major (PM) muscle to classify type I and II myofibers. a, Dimensionality reduction and clustering of 1,822 and 2,607 spots from replicate 1 (upper) and replicate 2 (lower) colored by cluster affiliation. The identified putative myofiber/spot clusters are annotated based on *MYH* transcriptional profiles (right side of heatmap), as well as the marker gene transcription and distribution from plots in **(b)**. **b,** Spatial features of gene transcription. Spatially resolved heatmaps across tissue sections from replicate 1 and replicate 2,

showing spatial patterns of myofiber marker gene transcription, *i.e., MYH7* (type I), *MYH4,* and *MYH2* (type II). The color bar indicates the transcription level. **c,** Mapping of data points from two putative myofiber clusters to their spatial positions. Scale bars, 500 μm. **d,** ATPase staining of PM tissue sections (adjacent to those in **c**). Scale bars, 500 μm. A representative image of two independent experiments is shown. The locations of putative type I myofibers in **(c)** are matched according to myofiber types identified from the ATPase-stained histological regions in **(d)**. Magnified images are shown at the bottom. **e,** Functional enrichment for differentially expressed genes between type I and II myofiber clusters. The sizes of dots represent the numbers of enriched genes, and the color represents the $\log_{10}$(*q*-value) (adjusted). *P* values are calculated based on a one-sided accumulative hypergeometric test. *q*-values are calculated using the Banjamini-Hochberg procedure to account for multiple testings.

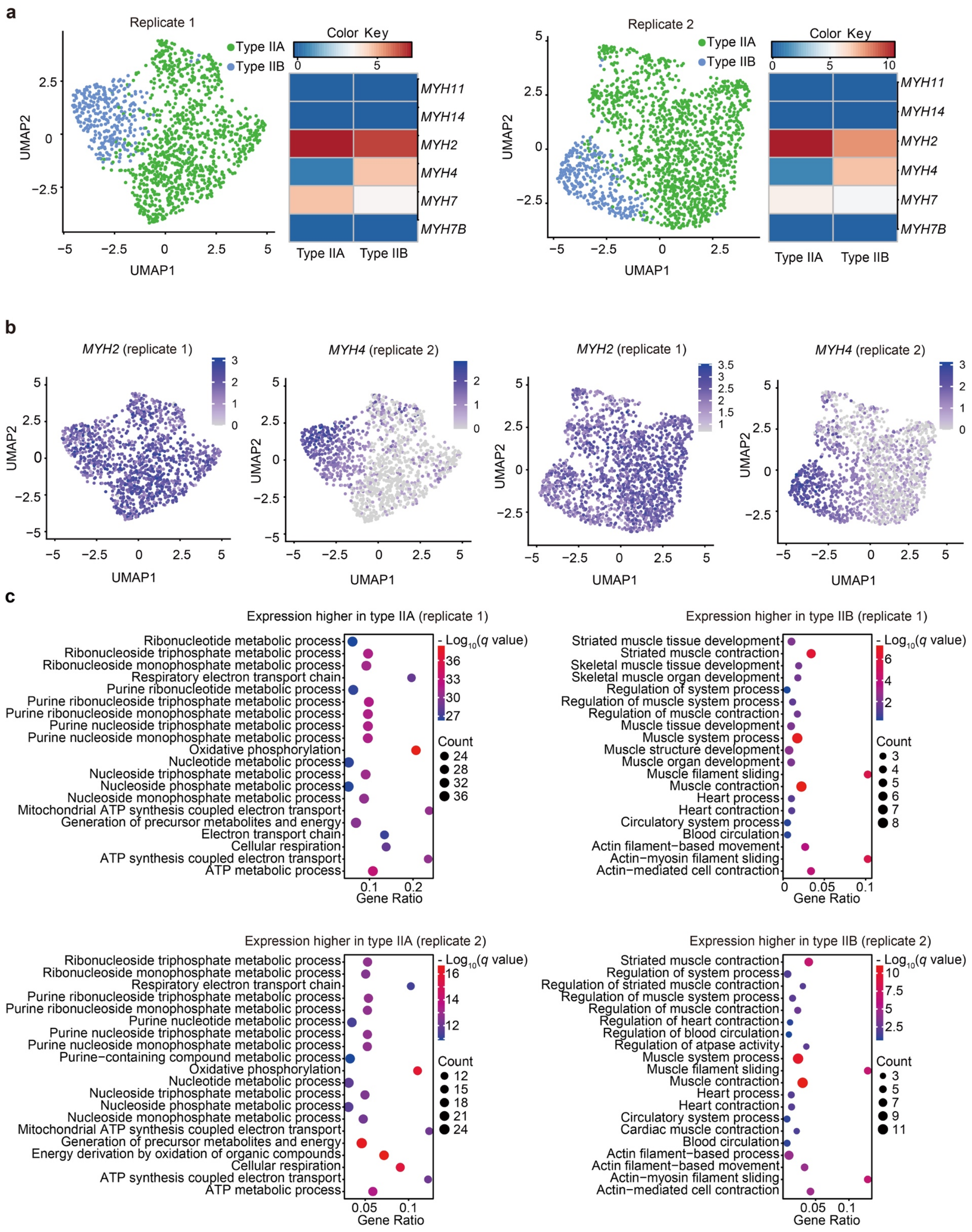

Supplementary Fig. 22. Dimensionality reduction and clustering of spots to classify the type IIA and IIB myofibers. a, Dimensionality reduction and clustering of 1,277 (replicate 1, left) and 1,677 (replicate 2, right) spots that were identified as type II myofiber clusters, as shown in **Supplementary Fig. 21**. The putative myofiber/spot clusters are annotated based on *MYH* transcriptional profiles (right side of heatmap), as well as marker gene transcription and distribution in the plots in **(b)**. **b,** Spatial features of gene transcription. Spatially resolved heatmaps across tissue sections from replicate 1 and replicate 2, showing spatial patterns of myofiber marker gene transcription, *i.e.*, *MYH2* (type IIA) and *MYH4* (type IIB). The color bar indicates the transcription level. **c,** Functional enrichment for differentially expressed genes between type IIA and IIB myofiber clusters.

The sizes of dots represent the numbers of enriched genes, and the color represents the $\log_{10}$(q-value) (adjusted). P values are calculated based on a one-sided accumulative hypergeometric test. q-values are calculated using the Banjamini-Hochberg procedure to account for multiple testings.

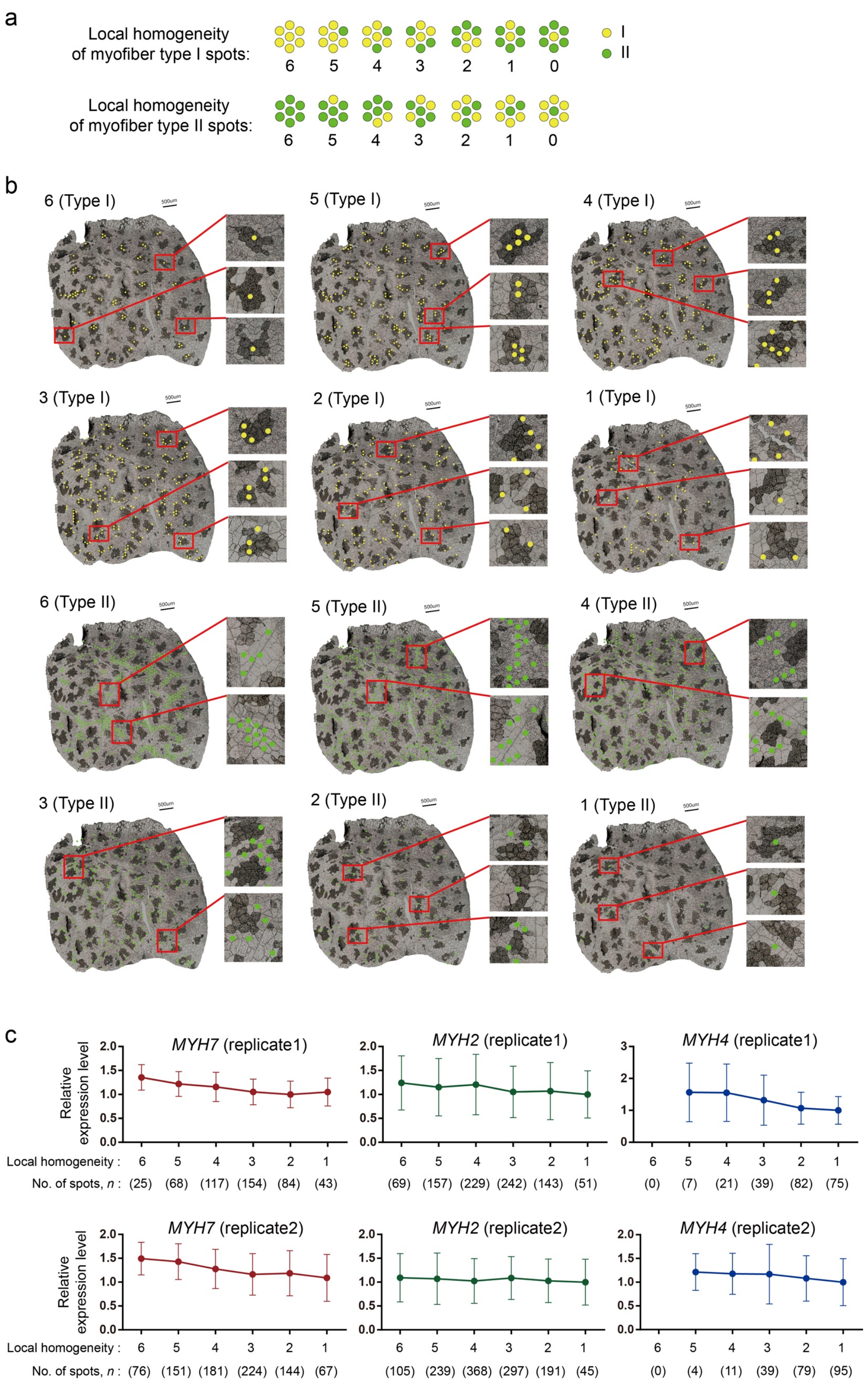

Supplementary Fig. 23. Assessment of homogeneity of myofibers/spots based on local diversity of myofiber types. a, Schematic diagram for scoring local homogeneity

(LH) of type I and type II myofibers, respectively. The LH value of the center spot is calculated based on the homogeneity relationship of the 6 spots surrounding it. For example, for type I myofibers, if all 6 adjacent spots homogeneously belong to type I, the LH value of the center type I spot is calculated as 6. Therefore, all the spots were classified into six groups from 6 to 1. Spots with LH 0, indicating that none of the surrounding spots belong to the same type, were excluded. **b,** Comparison of the spatial positions of different LH-grouped spots. Spots of each LH group (yellow or green dots) are overlaid to the ATPase-stained image (background), where dark and light areas indicate type I and type II myofibers, respectively. Two or three representative fields of view are shown for each group. The results were generated using two replicates. The results of type I muscle fiber image analysis show that the spot groups with higher LH values (6, 5) exactly overlap with dark stained areas where the type I muscle fibers were located. As the LH score decreases, the spots gradually shifted outward to the light-colored area (type II spot areas). **c,** Distribution of relative expression levels of myofiber marker genes (*i.e., MYH7* for type I, *MYH2* for type IIA, and *MYH4* for type IIB) across different LH groups classified using the LH scoring index shown in (**a**), for each of three types of myofiber. The expression levels (UMI counts) were normalized to the minimum expression of six LH groups. Data are presented as mean values ± SD (number of spots is listed in the parenthesis).

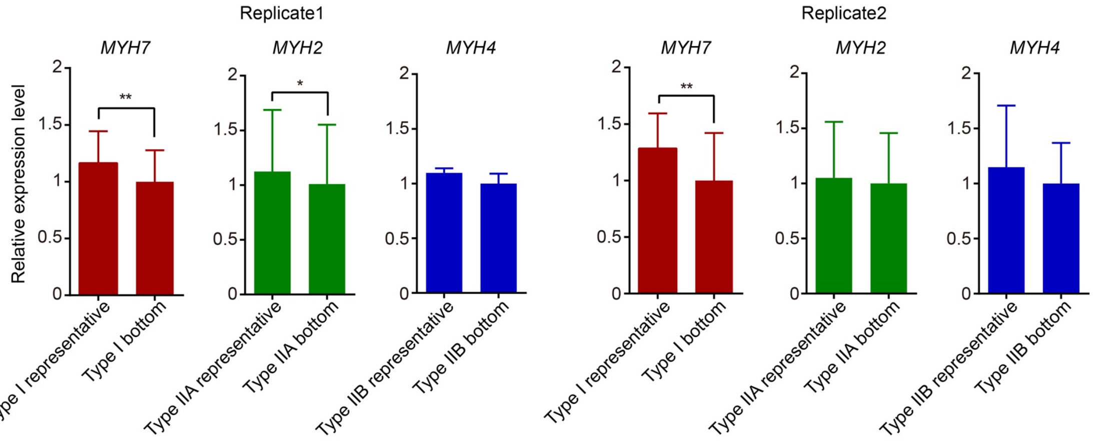

Supplementary Fig. 24. Marker gene expression in representative spots for three types of myofiber clusters. Bar plots show higher expression level of marker genes in representative spots (top 200 spots with highest LH value, n = 200) compared to 200 spots (n = 200) with lowest LH values. Error bars represent standard deviation (SD). Mann Whitney test was used to determine significance. *, $P < 0.05$; **, $P < 0.01$.

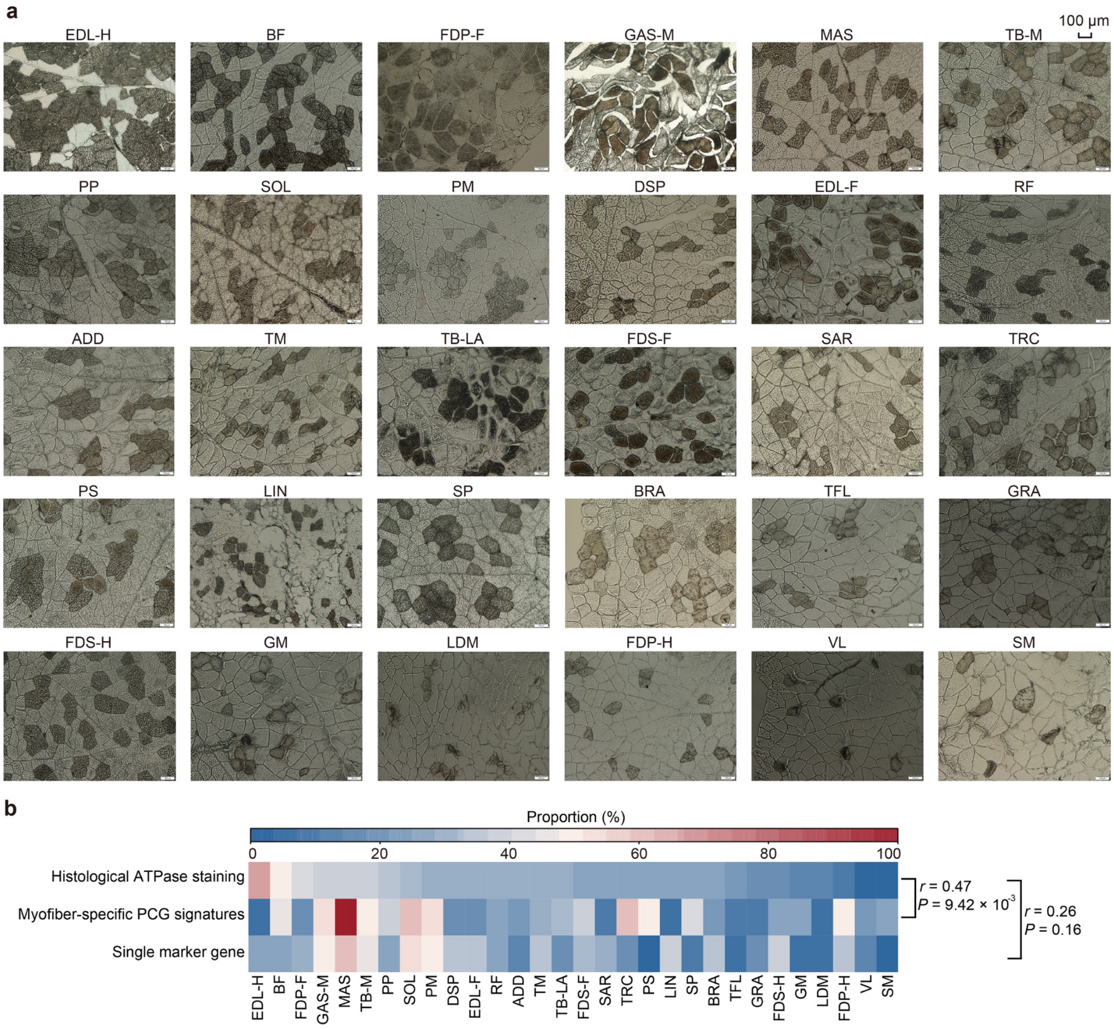

Supplementary Fig. 25. Verification of the estimated myofiber proportions using ATPase staining of SMTs. a, ATPase staining of histological sections of 30 SMTs. Type I myofibers are indicated by a dark stain. Scale bars, 100 μm. A representative image of two independent experiments is shown for each SMT. **b,** Proportions of type I myofiber estimated using the assembly of myofiber-specific PCG signatures (middle) and a single marker gene (*e.g.*, *MYH7*) (lower) are compared to the type I myofiber proportions determined by histological ATPase staining (calculated as the number of type I myofibers versus all the myofibers in the section) (upper), with Spearman's correlation coefficients of 0.62 and 0.41, respectively. The statistical significance of the two-sided *P* value was calculated using hypothesis testing. The SMTs are ranked according to the histological ATPase staining-based type I myofiber proportions from high (left) to low (right).

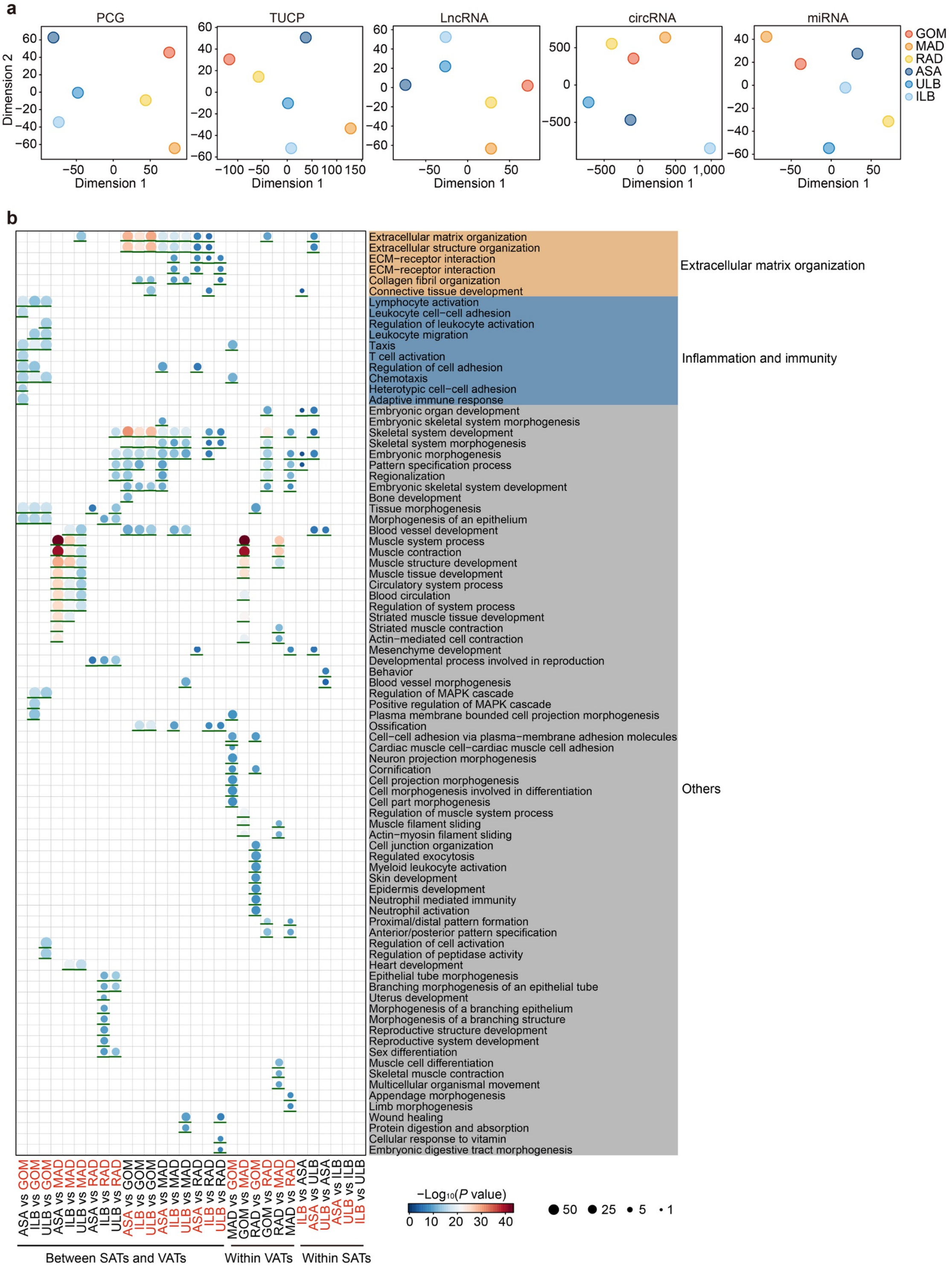

Supplementary Fig. 26. Transcriptional divergence of distinct transcript types across adipose tissues (ATs). a, Multidimensional scaling through t-SNE distances based on log_2-transformed transcription levels of different transcript types. **b,** Functional enrichment for differentially expressed (DEs) PCGs between ATs. Plot showing the top 10 enriched GO terms of upregulated DE PCGs in pairwise AT comparisons. The pairwise comparisons listed at the bottom are sorted as follows: between SATs and VATs, within SATs, and within VATs. The names highlighted in red for each pairwise comparison

represent the tissue in which the genes were highly expressed. The color shades on the Y-axes highlight the categories of GO terms, *i.e.,* extracellular matrix organization related terms (orange), inflammation and immunity related terms (blue), and others (grey). The sizes of dots represent numbers of enriched genes, and dot color represents the -$\log_{10}$(*P*-value) (unadjusted). *P* values are calculated based on a one-sided accumulative hypergeometric test. Heights of lines in each square indicate the ratio of enriched genes.

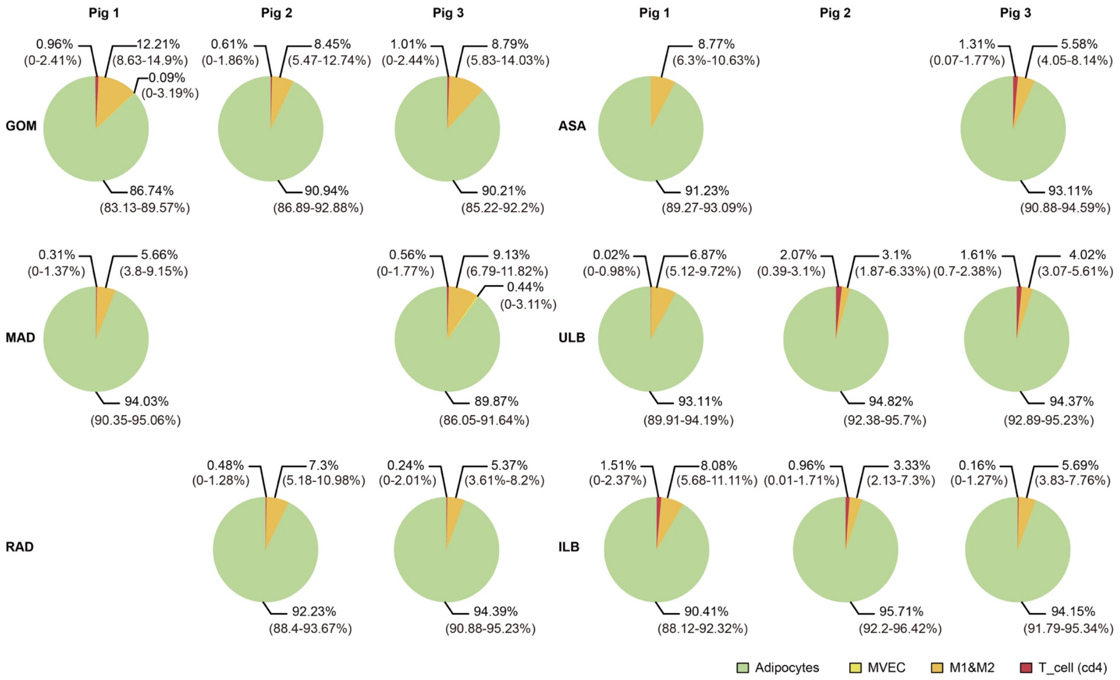

Supplementary Fig. 27. Estimates of the proportions of different cell types in ATs. Percentages next to pie plots show the proportion of each cell type, including adipocytes, microvascular endothelial cells (MVECs), macrophages (M1&M2 combined), and CD4+ T-cells. We also calculated the confidence range for cell type proportions using a bootstrap (1000 times) method that selects a random subset of 80% of the markers. The percentages in parentheses denote the confidence range for each estimated percentage.

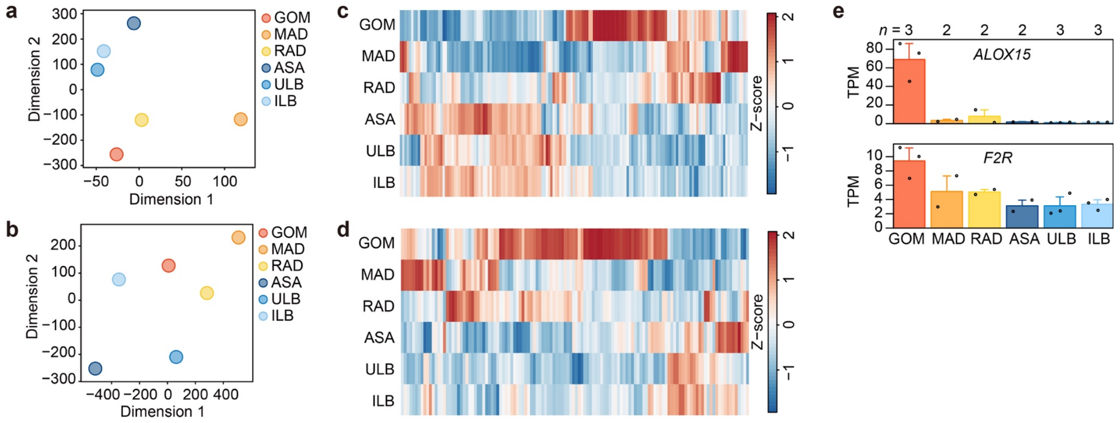

Supplementary Fig. 28. Transcriptional divergence of inflammation-related characteristics across ATs. a-b, t-SNE plots based on log_2-transformed transcription levels of **(a)** 528 functional PCGs related to 'inflammatory response'; and **(b)** 400 ECM-related PCGs. **c-d,** Heatmap showing the patterns of differentially expressed (DE) PCGs related to **(c)** 'inflammatory response', and **(d)** ECM. The expression levels were standardized by Z-score (mean of zero and s.d. of one) for each gene. The color bar indicates Z-score range of expression level, calculated as standard deviation from average expression level divided by standard deviation. **e,** Histogram showing the transcription of representative inflammatory PCGs (*i.e.*, *ALOX15* and *F2R*). Data are presented as mean values ± SD (*n* number is listed above each bar)

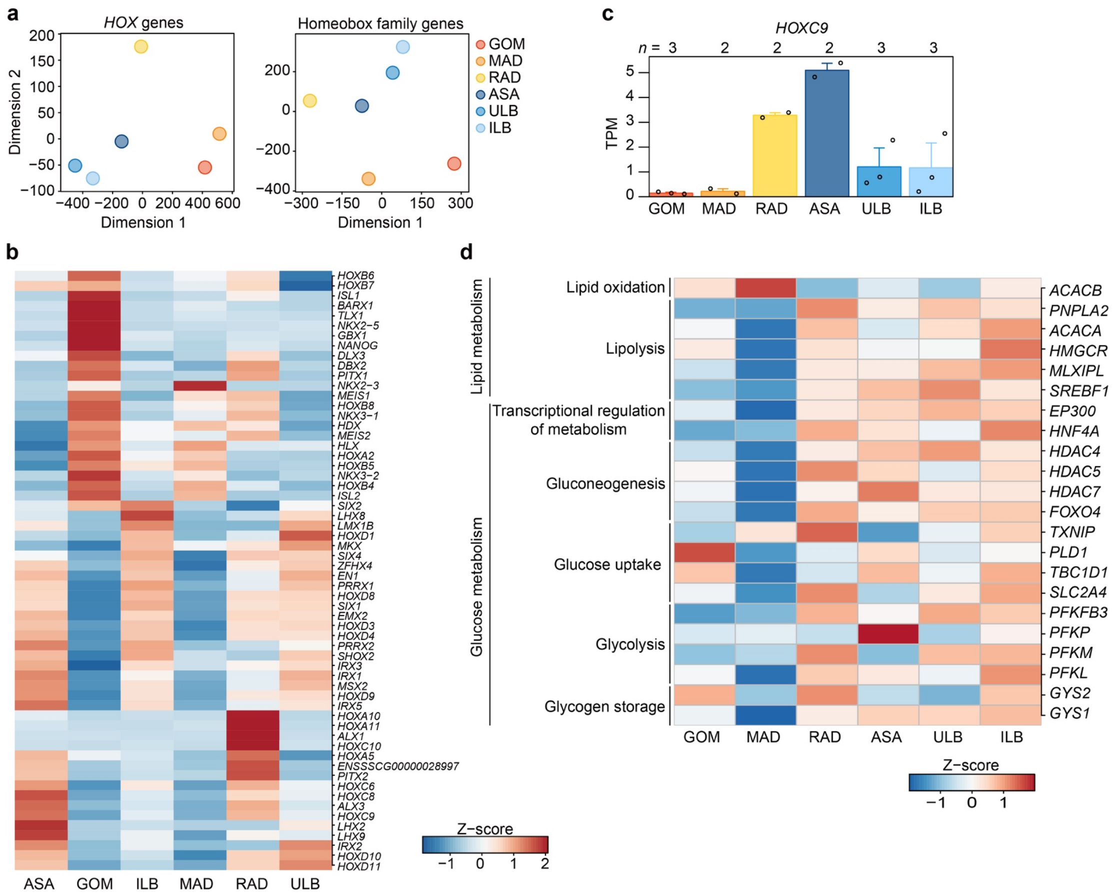

Supplementary Fig. 29. Transcriptional patterns of *HOX* genes and homeobox family genes suggesting the developmental origins of ATs. a, t-SNE plots based on log2-transformed transcription levels of 29 HOX genes (left) and 84 homeobox family genes (right). **b,** Heatmap showing the patterns of differentially expressed (DE) *HOX* genes and homeobox family genes. **c,** The histogram indicates the high transcription level of the *HOXC9* gene in three SATs, as well as in the original SAT-like RAD. Data are presented as mean values ± SD (*n* number is listed above each bar). **d,** The transcriptional patterns of 22 key genes involved in glucose and lipid metabolism regulated by AMPK indicate that the metabolic characteristics of RAD are more similar to those of SATs. Z-score standardization was the same as described in **Supplementary Fig. 28.**

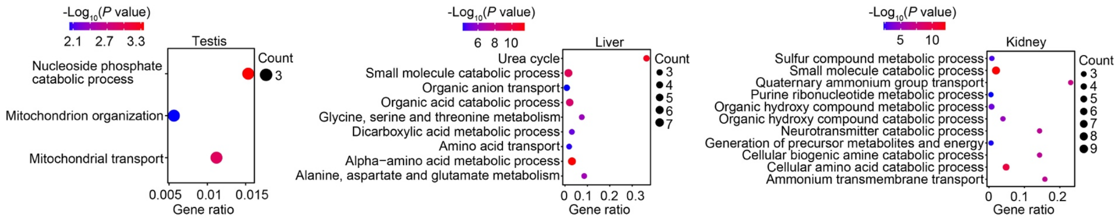

Supplementary Fig. 30. Functional enrichment of tissue-specific mt-localized PCGs. Here, only the enriched terms for tissues with >10 tissue-specific mt-localized PCGs (*i.e.*, testis, liver, kidney) are shown. The sizes of dots represent the numbers of enriched genes, and the color represents the $\log_{10}$(*P*-value) (unadjusted). *P* values are calculated based on a one-sided accumulative hypergeometric test

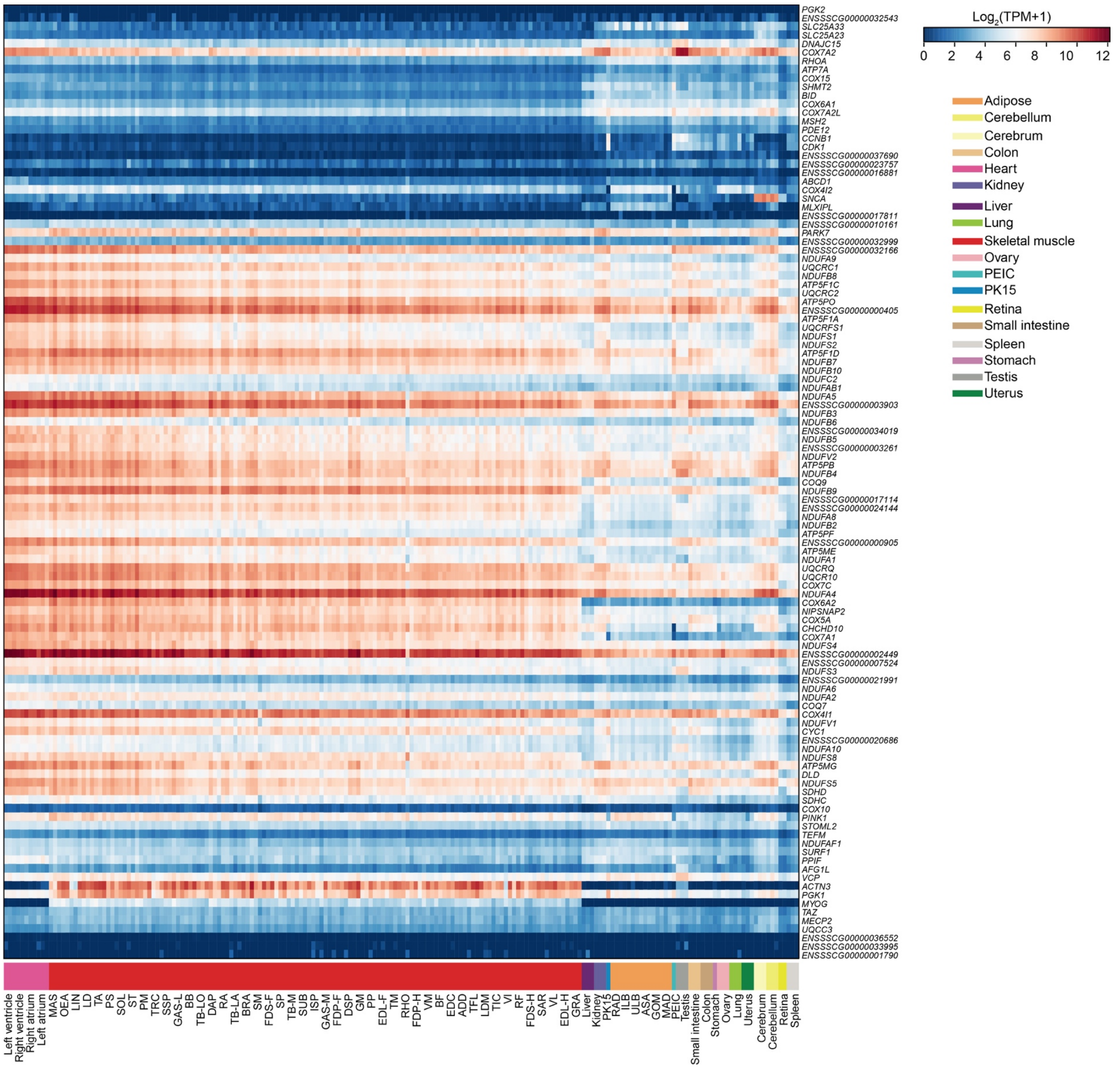

Supplementary Fig. 31. Heatmap showing the transcriptional patterns of OXPHOS-related mt-localized nu-PCGs across all tissues.

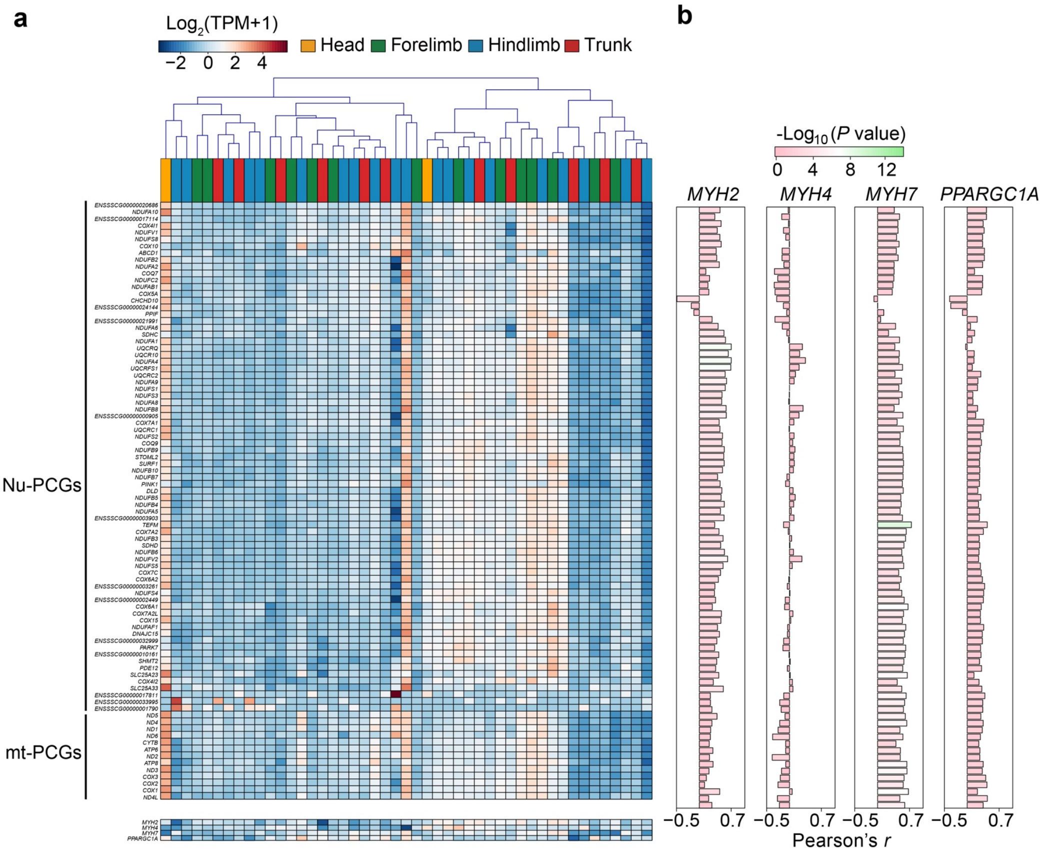

Supplementary Fig. 32. Transcriptional pattern of OXPHOS-related PCGs across SMTs from different anatomical regions. a, Heatmap showing the transcription levels of mt-localized nu-PCGs (upper), mt-PCGs (middle) related to OXPHOS, *MYH*s (bottom), and *PPARGC1A* (bottom) (*i.e., PGC-1α,* a marker gene reported to increase mitochondrial oxidative capacity). Grid color indicates z-score-normalized relative transcription levels. **b,** The bar plot indicates correlation between the transcription of each OXPHOS PCG (each row to the left of heatmap) and the abundance of each *MYH* or the abundance of *PPARGC1A.* The histogram indicates the Pearson correlation coefficient, while the color indicates the two-sided statistical significance of the *P* value.

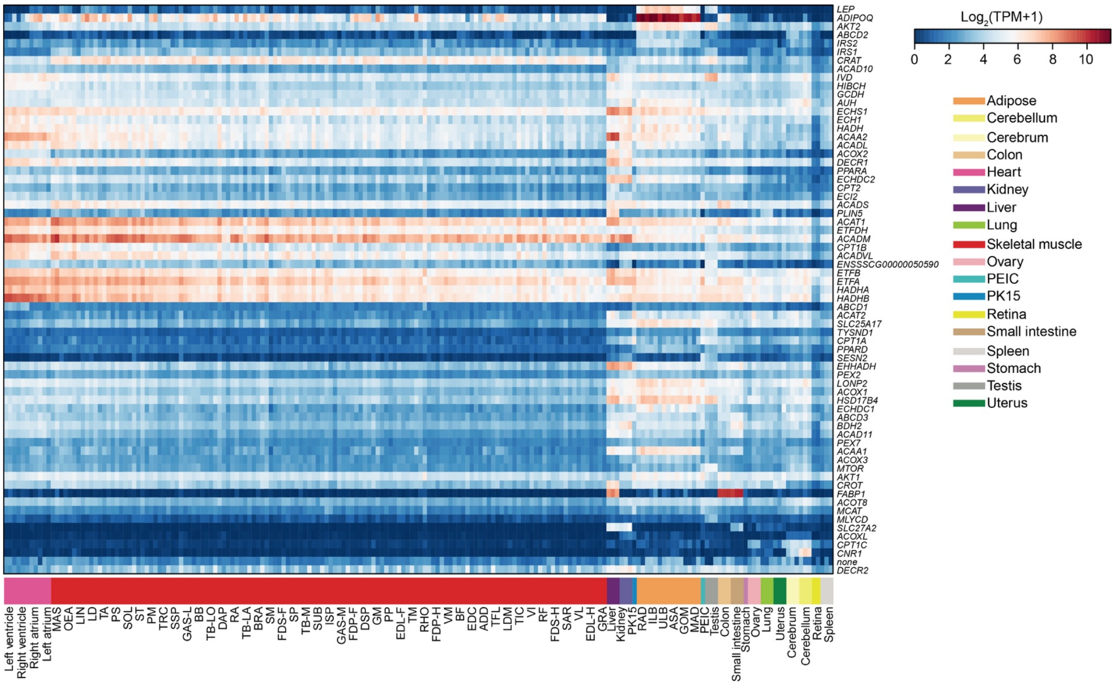

Supplementary Fig. 33. Heatmap of transcriptional patterns of FAO-related mt-localized nu-PCGs across all tissues.

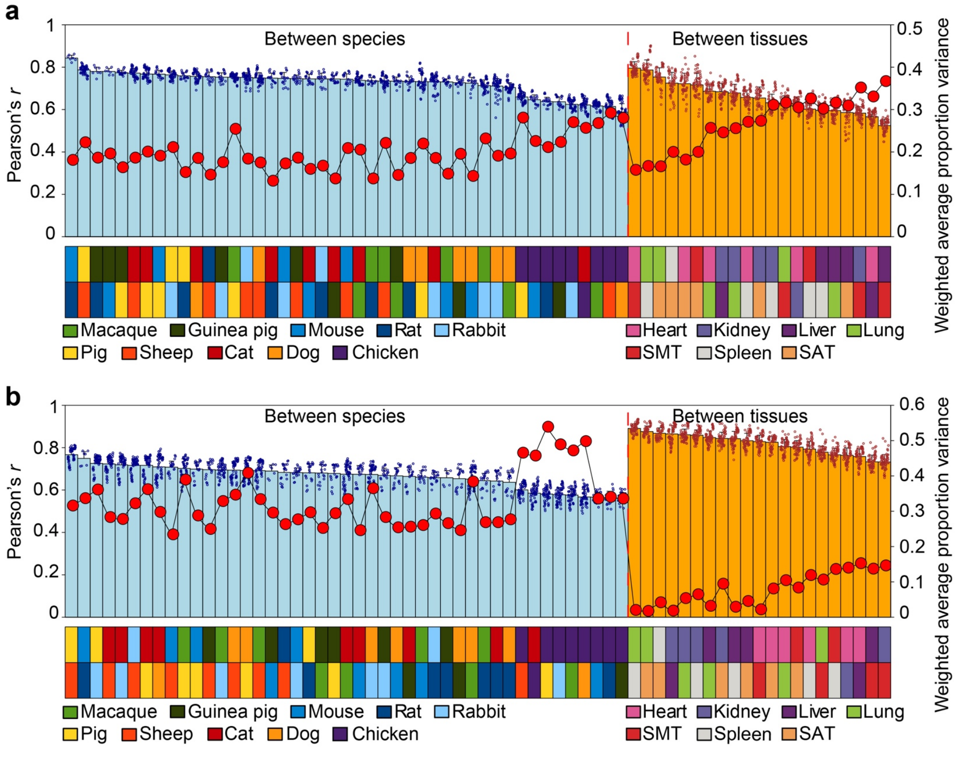

Supplementary Fig. 34. Comparison of variation between species (nine mammals and chicken) and tissues revealed by (a) gene transcription and (b) alternative splicing patterns. Correlations were calculated based on the transcription levels of 6,433 1-1 orthologs and 12,662 orthologous exons across 10 species, including 9 mammals and chicken. Bar plots represent pairwise Pearson's correlations between species (light blue

bar) and between tissues (orange bar). Data are presented as mean values ± SD. Principal variance component analysis (PVCA) was used to determine the weighted average of proportional variances for levels of gene transcription and alternative splicing (reflected by PSI values), which are depicted as red dots connected by black lines. Boxes (bottom) indicate pairwise comparisons presented in each column according to the color assigned to each species or tissue.

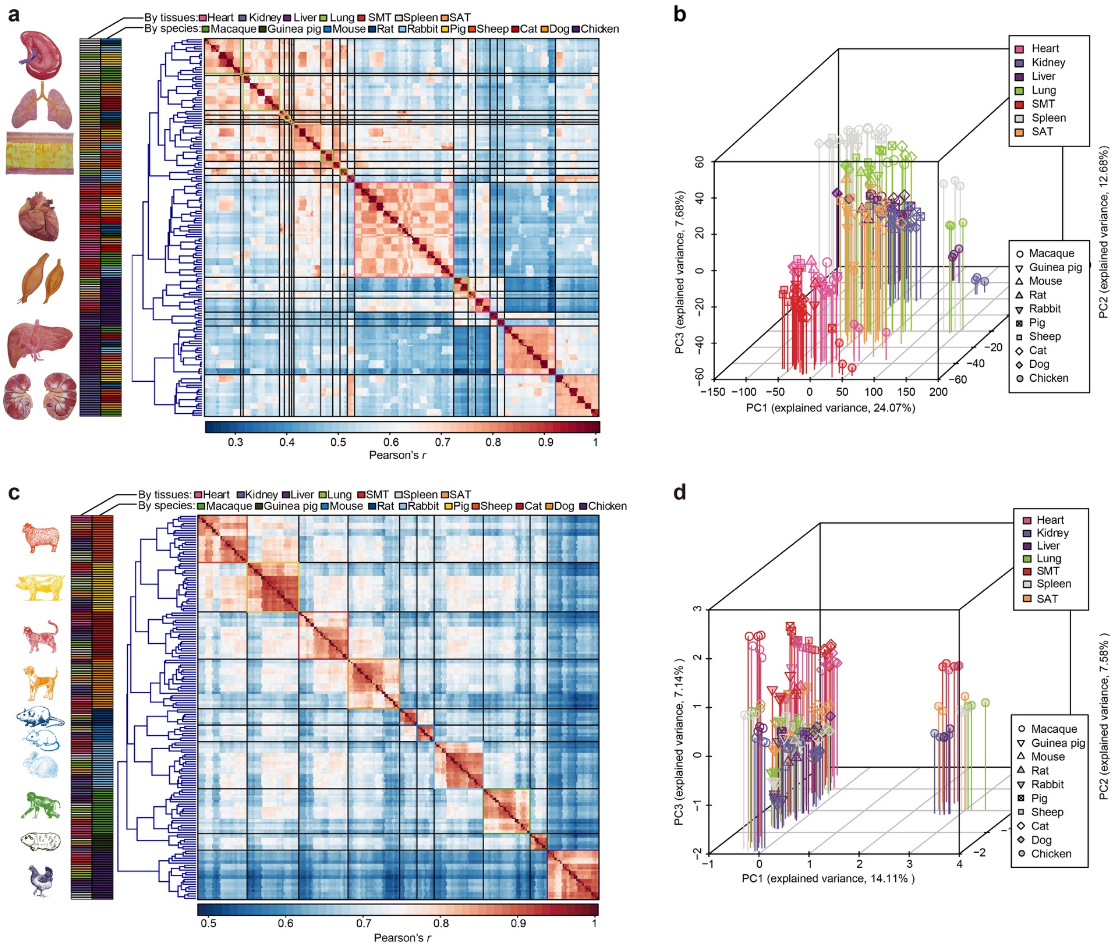

Supplementary Fig. 35. Global patterns of gene transcription and alternative splicing. **a,c,** Hierarchical clustering analysis of samples using **(a)** gene transcription and **(c)** alternative splicing (reflected by PSI values). Average linkage hierarchical clustering was based on distances between transcription levels of samples measured by Pearson's correlation. **b,d,** Factorial map of the principal component analysis (PCA) of **(b)** gene transcription levels and **(d)** alternative splicing. The proportion of variance explained by the principal components is indicated in parentheses. The vertical lines of different colors dropping from the plotted points to the x/y plane show the separation of points based on the first and second principal components.

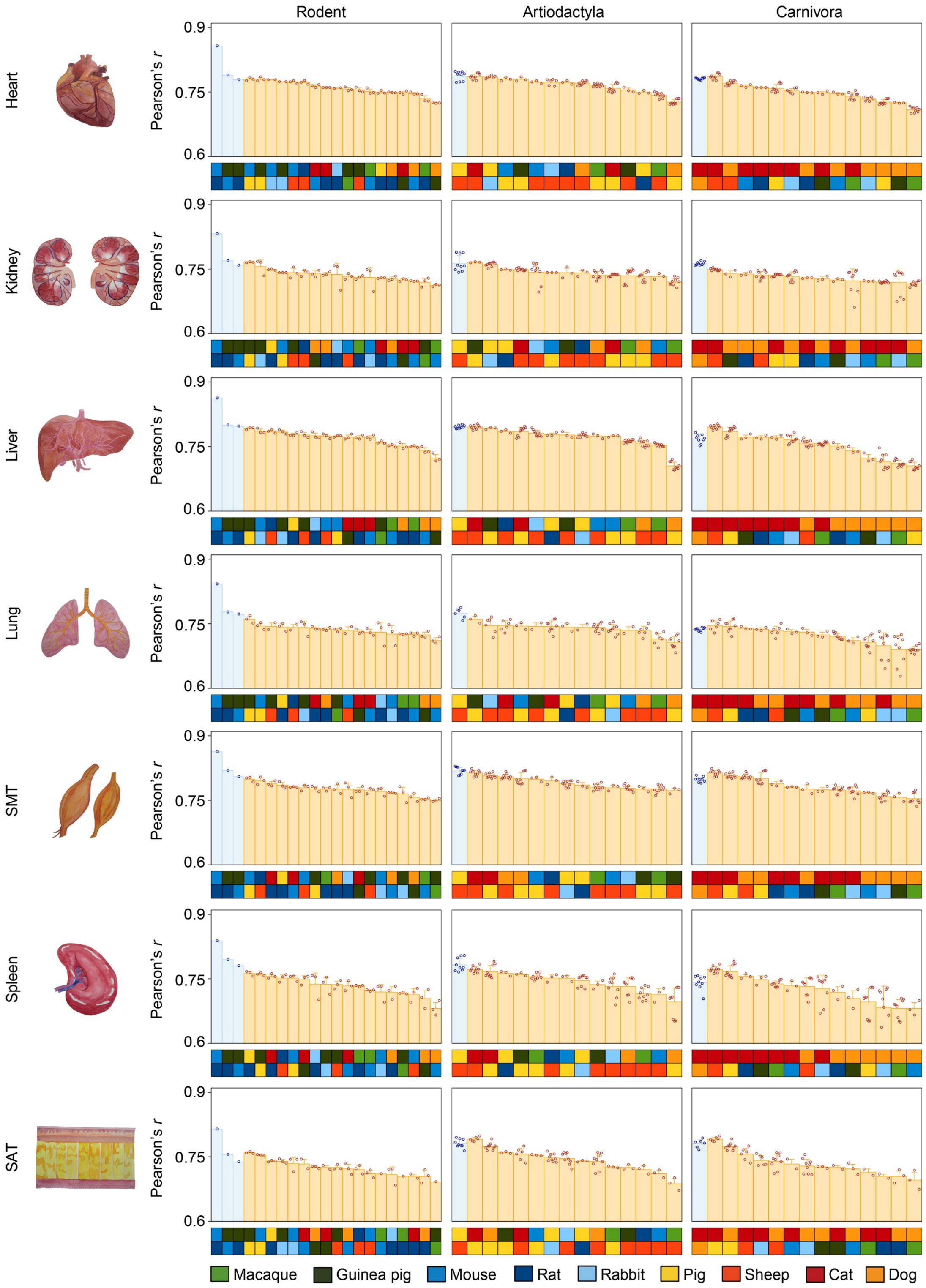

Supplementary Fig. 36. Correlation-based transcription levels potentially reflecting divergence within mammals. Bar plots represent pairwise Pearson's correlations between species, with light blue bars indicating correlations within genera and yellow bars indicating correlations between species in the indicated genus and other species, *i.e.*, rodents (left), artiodactylids (middle), and carnivores (right). Boxes (bottom) indicate pairwise comparisons presented in each column according to the color assigned to each species (bottom of the plot). Data are presented as mean values ± SD.

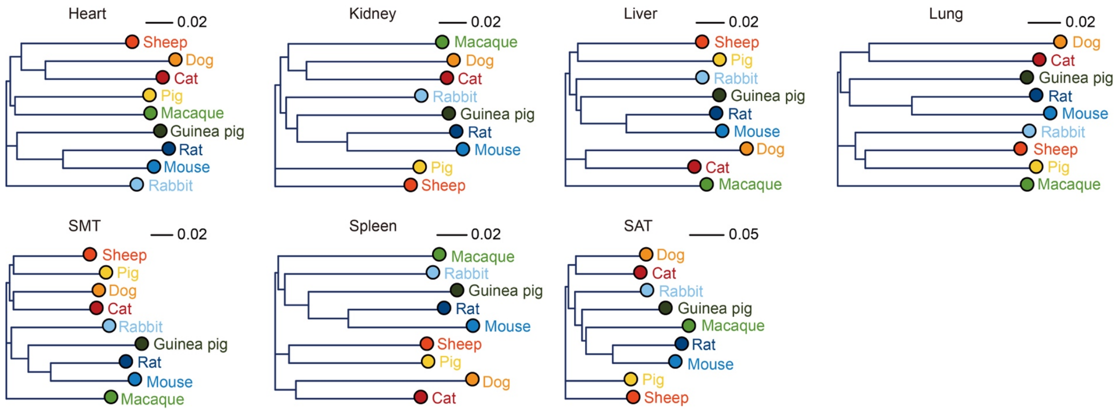

Supplementary Fig. 37. Gene transcription phylogenies for 7 tissues across 9 mammals. We reconstructed neighbor-joining tree based on distances (1-*r,* here, *r* is Spearman’s correlation coefficient) between transcription levels of single-copy orthologs. The total branch lengths of each tree were extracted to estimate the evolutionary divergence of transcription profiles between tissues, as presented in **Fig. 6g**. The scale bar represents distances.

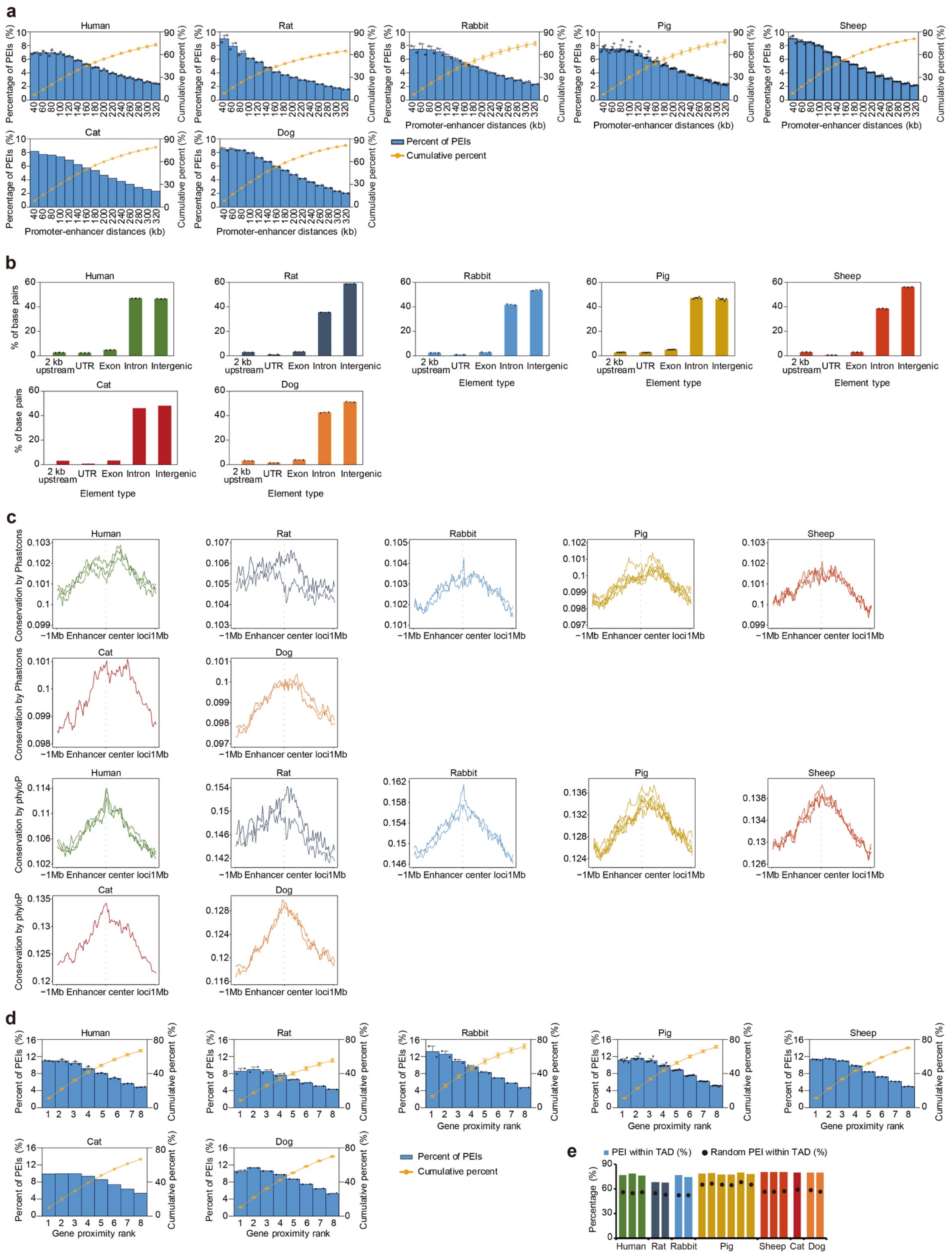

Supplementary Fig. 38. Characteristics of promoter–enhancer interactions (PEIs). a, Distribution (blue bars) and cumulative distribution (orange dots) of PEIs for each species, as predicted using PSYCHIC. Overall, ~37.27% (33.92% in human to 40.91% in dog) of the enhancers were located within 120 kb of their target promoters. Data are presented as mean values ± SD (human *n* = 3; rat *n* = 2; rabbit *n* = 2; sheep *n* = 3; cat *n* = 1; dog *n* = 2; pig *n* = 6). **b**, Distribution of enhancer sequences across genomic features. The y-axis shows the percentage of total enhancers overlapping with different genomic features.

These enhancers tend to overlap with intron and intergenic regions. Data are presented as mean values ± SD (human *n* = 3; rat *n* = 2; rabbit *n* = 2; sheep *n* = 3; cat *n* = 1; dog *n* = 2; pig *n* = 6). **c**, Conservation of enhancers in vertebrates. The x-axis depicts the start and end of enhancers flanked by 1 Mb of adjacent sequence. The y-axis represents sequence conservation calculated by 100-vertebrate phastCons or phyloP values. Enhancers showed higher sequence conservation than their immediate flanking regions. **d,** Distribution (blue bars) and cumulative distribution (orange dots) of the proximity rank of genes associated with enhancer bins. Only about 10.77% (~8.53% in rat to 13.14% in rabbit) of enhancers regulate the nearest gene. Data are presented as mean values ± SD (human *n* = 3; rat *n* = 2; rabbit *n* = 2; sheep *n* = 3; cat *n* = 1; dog *n* = 2; pig *n* = 6). **e,** Percentage of PEIs within TADs. Black dots show percent of "random" enhancers residing within the same TAD. As expected, most (~68.02% in rat to 80.45% in sheep) of the detected interactions fall within the topologically associating domains (TADs), compared to 57.70% in random shuffles.

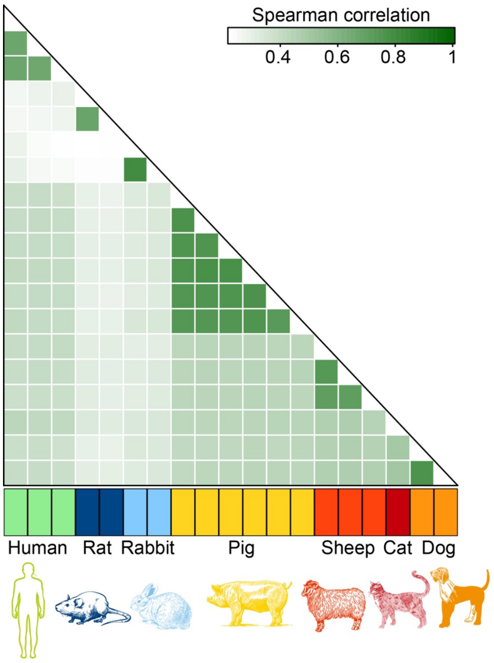

Supplementary Fig. 39. Landscape of enhancer correlations across species. The numbers of associated/interacting enhancers for a gene (9,540 1-1 orthologs) were correlated between all pairs of species. The shading of individual tiles corresponds to pairwise Spearman's correlation coefficients for numbers of enhancers of orthologous genes across nine mammalian species.

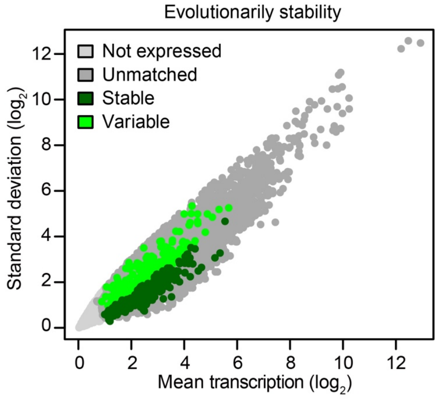

Supplementary Fig. 40. Transcribed genes with evolutionarily stable and variable transcription levels were identified based on their coefficient of variation across species (CV; standard deviation normalized by mean expression). Variable genes (top 50% of the CV distribution) are highlighted in light green. Stable genes (bottom 50% of the CV distribution) are highlighted in dark green. Genes in either category with no matched counterpart at the same transcription level in the other category were not considered for analysis (in dark gray, “unmatched”). Non-transcribed genes are shown in light gray (mean abundance < 1 TPM across species).

Heart

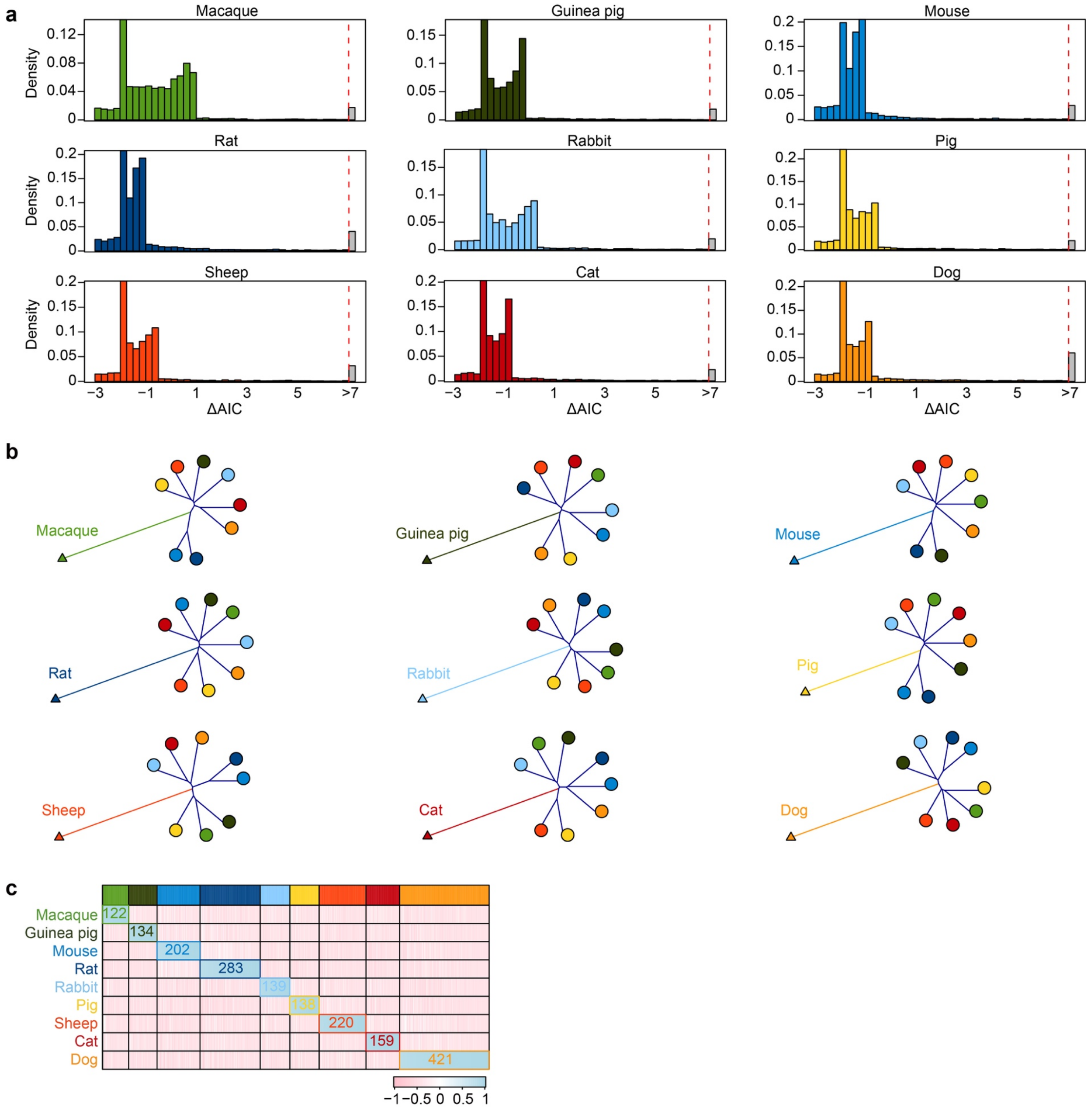
a
Macaque
Guinea pig
Mouse
Rat
Rabbit
Pig
Sheep
Cat
Dog
Density
ΔAIC
b
c
122
134
202
283
139
138
220
159
421

Kidney

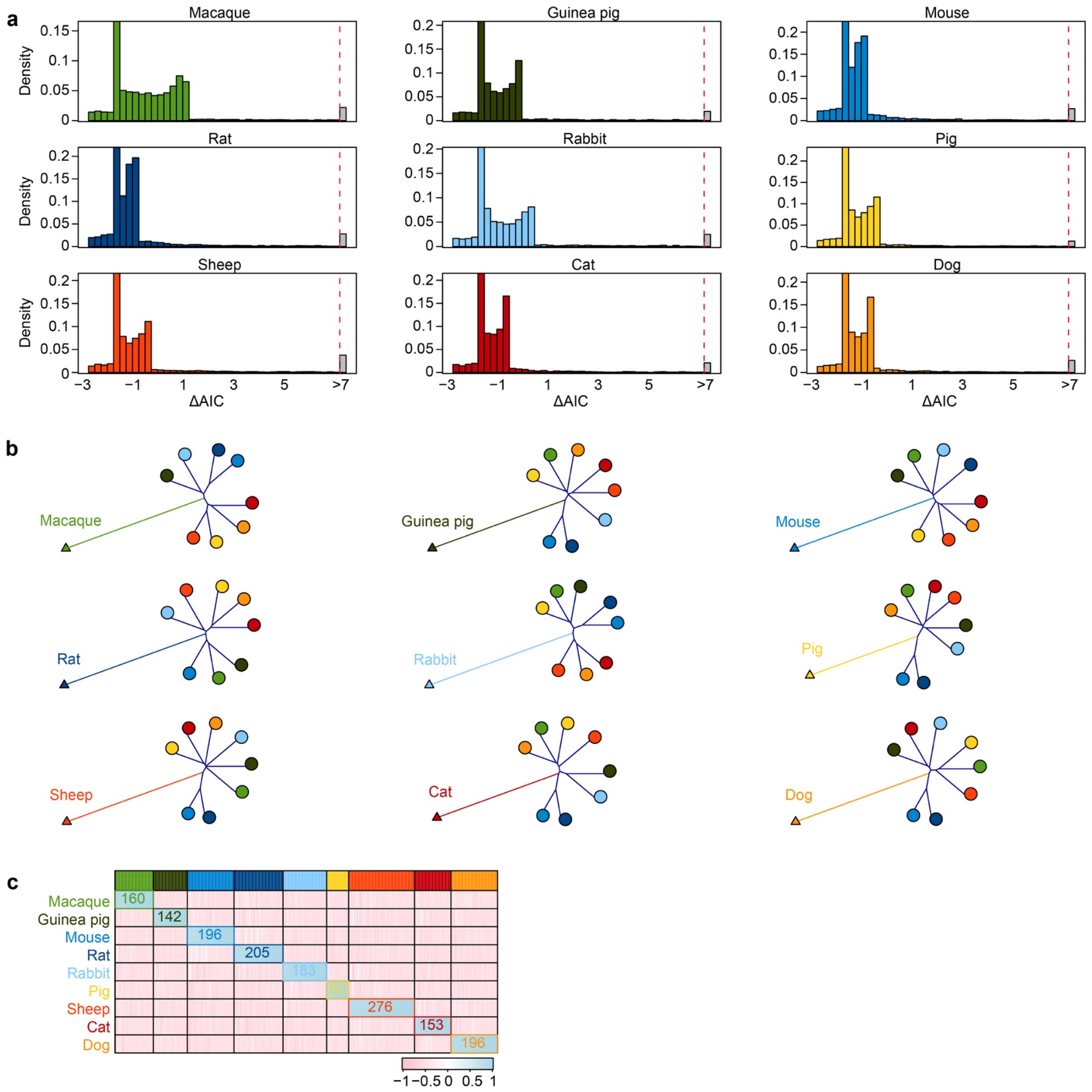

a
Macaque
Guinea pig
Mouse
Rat
Rabbit
Pig
Sheep
Cat
Dog
Density
ΔAIC
b
c
160
142
196
205
183
276
153
196

Liver

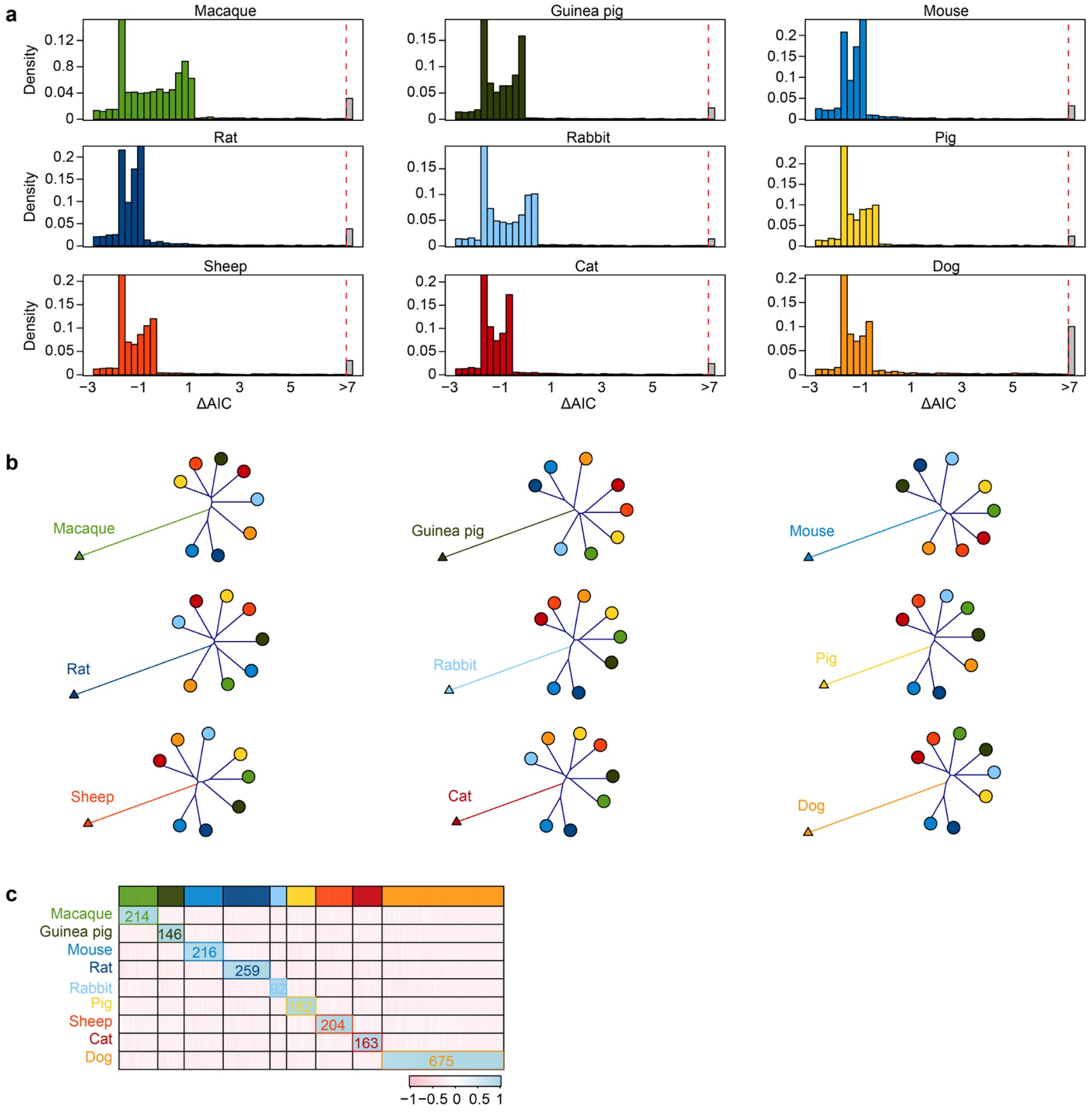

a
Macaque
Guinea pig
Mouse
Rat
Rabbit
Pig
Sheep
Cat
Dog
Density
0.12
0.8
0.04
0
0.15
0.1
0.05
0.2
−3
−1
1
3
5
>7
ΔAIC
b
Macaque
Guinea pig
Mouse
Rat
Rabbit
Pig
Sheep
Cat
Dog
c
Macaque
Guinea pig
Mouse
Rat
Rabbit
Pig
Sheep
Cat
Dog
214
146
216
259
204
163
675
−1 −0.5 0 0.5 1

Lung

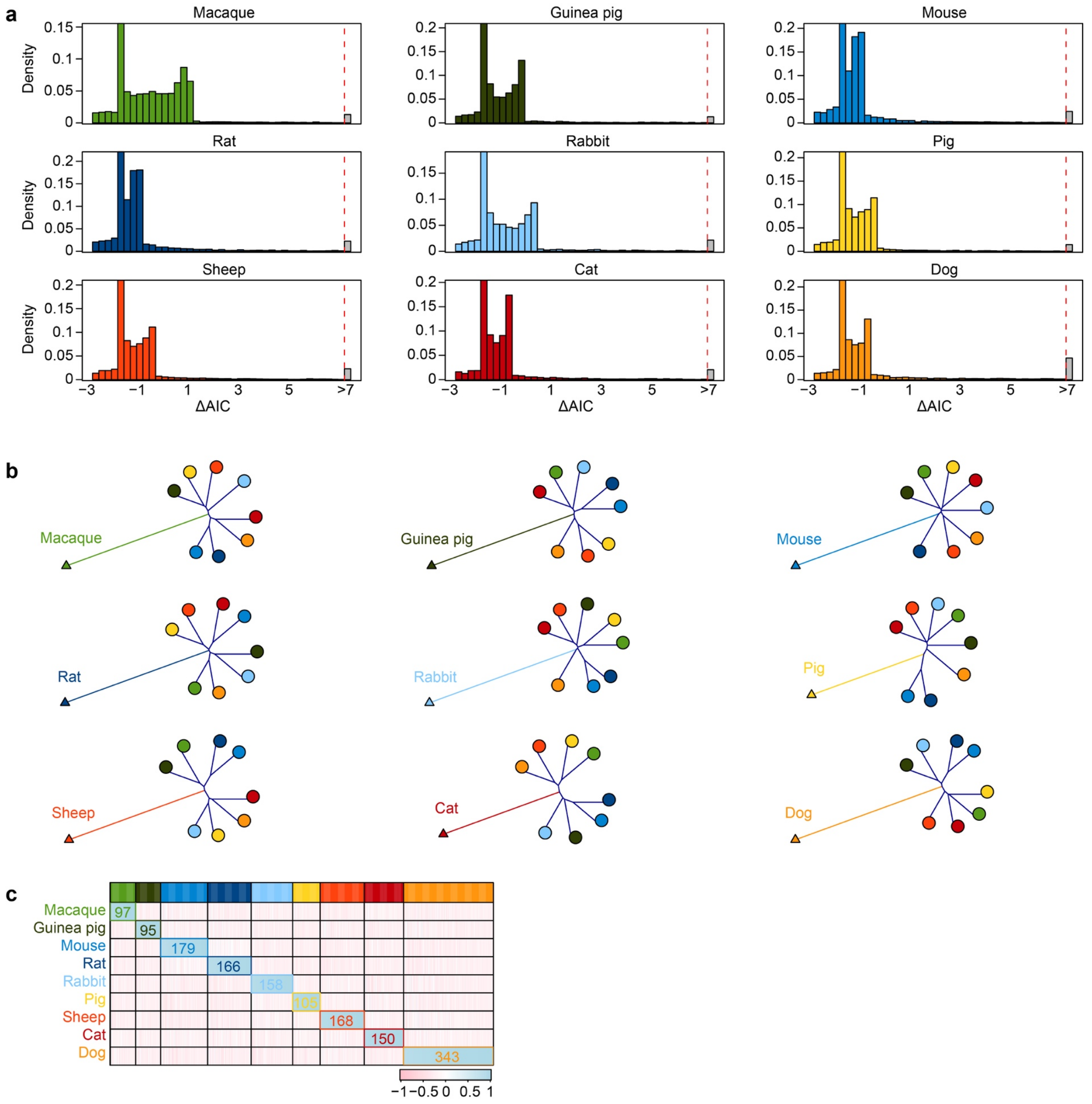

a
Macaque
Guinea pig
Mouse
Rat
Rabbit
Pig
Sheep
Cat
Dog
Density
ΔAIC
−3
−1
1
3
5
>7
b
Macaque
Guinea pig
Mouse
Rat
Rabbit
Pig
Sheep
Cat
Dog
c
Macaque
Guinea pig
Mouse
Rat
Rabbit
Pig
Sheep
Cat
Dog
97
95
179
166
168
150
343
−1 −0.5 0 0.5 1

SMT

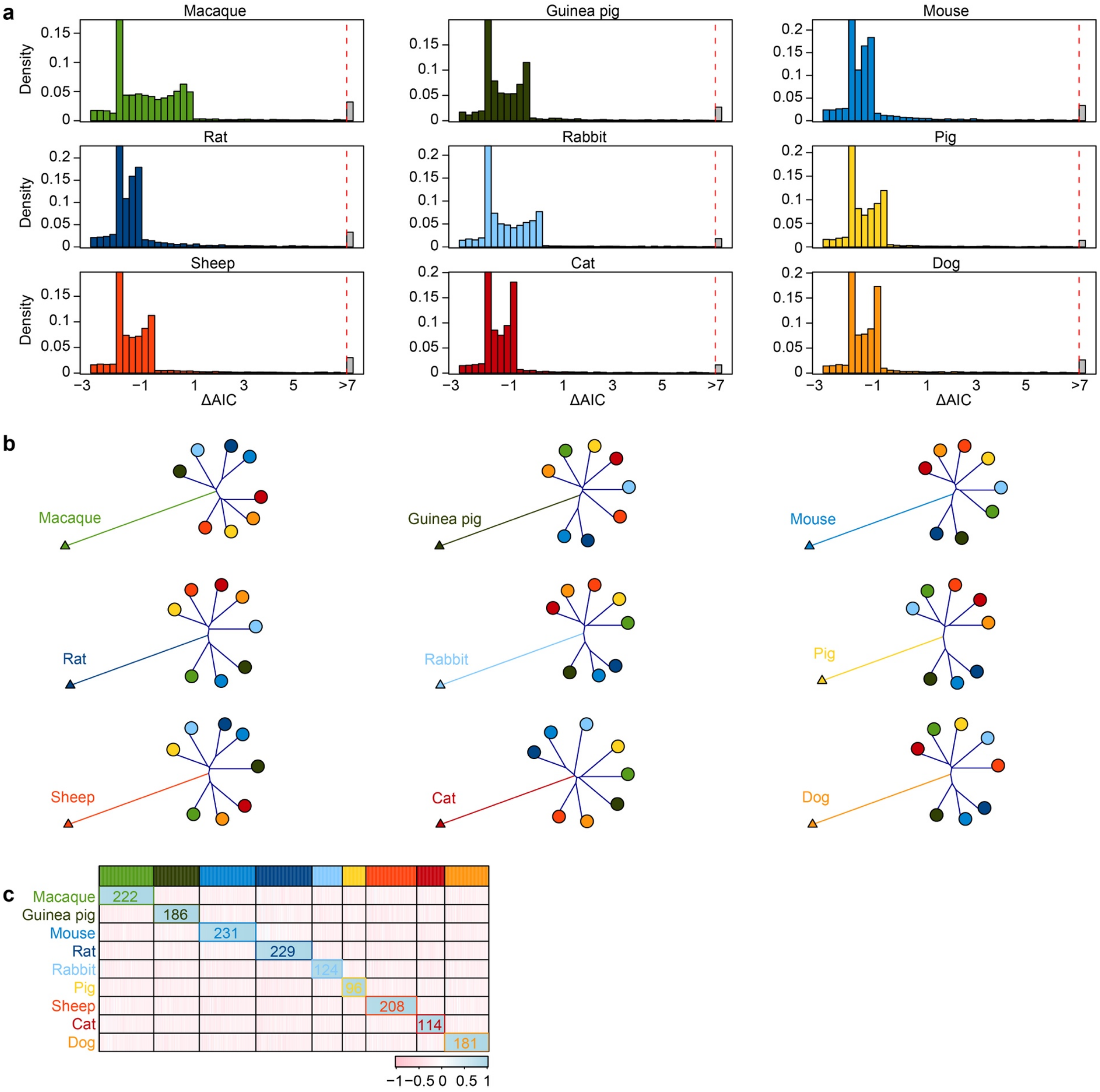
a
Macaque
Guinea pig
Mouse
Rat
Rabbit
Pig
Sheep
Cat
Dog
Density
ΔAIC
b
Macaque
Guinea pig
Mouse
Rat
Rabbit
Pig
Sheep
Cat
Dog
c
Macaque
Guinea pig
Mouse
Rat
Rabbit
Pig
Sheep
Cat
Dog
222
186
231
229
124
208
114
181
−1 −0.5 0 0.5 1

Spleen

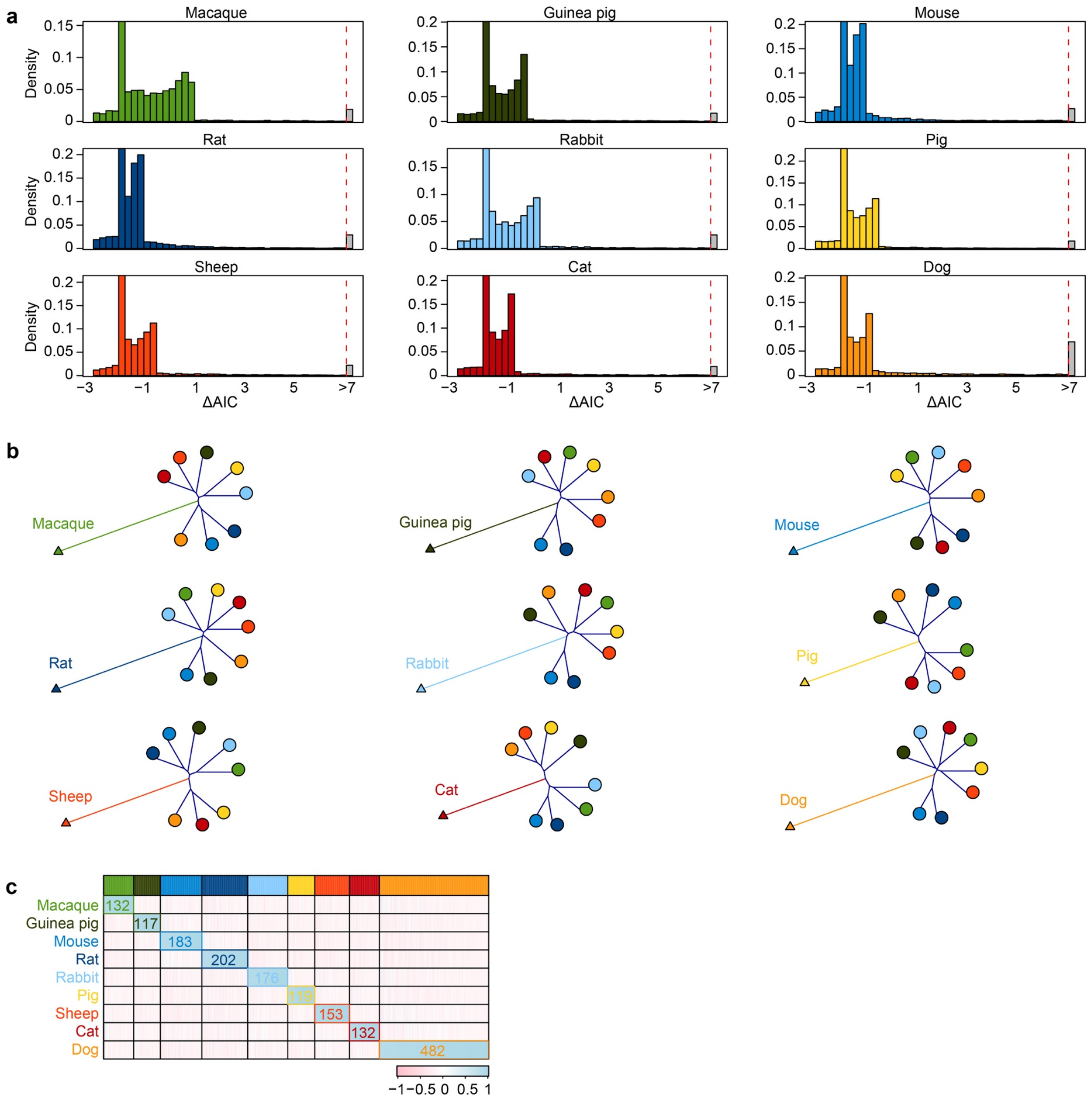

a
Macaque
Guinea pig
Mouse
Rat
Rabbit
Pig
Sheep
Cat
Dog
Density
ΔAIC
b
Macaque
Guinea pig
Mouse
Rat
Rabbit
Pig
Sheep
Cat
Dog
c
Macaque
Guinea pig
Mouse
Rat
Rabbit
Pig
Sheep
Cat
Dog
132
117
183
202
176
153
132
482
−1 −0.5 0 0.5 1

SAT

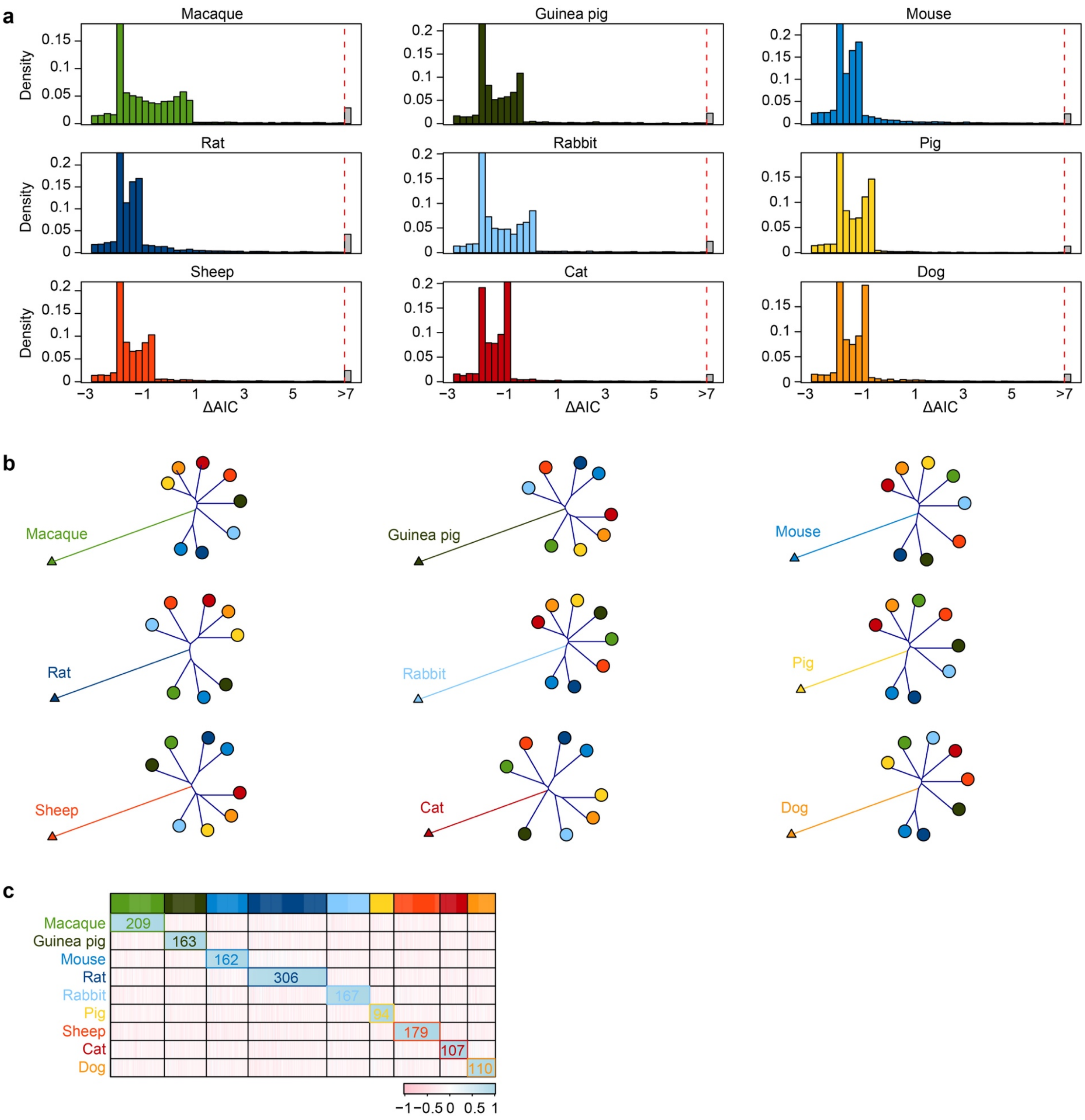

Supplementary Fig. 41. Genes with distinct transcriptional changes in each species and tissue. a, Histograms represent the distribution of change in the Akaike information criterion (ΔAIC) values. The red vertical dashed lines indicate the cut-off (ΔAIC≥7) for significant transcriptional changes. **b,** Phylogenetic tree for each tissue based on the transcription levels of genes with significant transcriptional changes in the indicated species compared with that in other species. **c,** Heatmap showing the transcription pattern of genes with significant transcriptional changes in the indicated species. Numbers inside the cells indicate the number of genes with significant transcriptional changes in the indicated species. The color bar indicates the normalized transcription level.

Supplementary Tables

Supplementary Table 1. Sample and data information for pig adipose Hi-C.

				Hi-C data								RNA-seq data				
Tissue	Age	Sex	Replicates	Sequenced read pairs	Alignable (Normal+ Chimeric Paired)	Unique reads (after removing PCR duplicates, intrafragment reads and low-mapping-quality reads)	Hi-C contacts	Inter chromosomal	Intra chromosomal	Intra-short range (<20 kb)	Intra-long range (>20 kb)	Raw reads	Clean reads	Raw bases (G)	Clean bases (G)	Q30 (%)
Upper layer of backfat (ULB)	Adult, 2-year-old	Female	1	614,903,276	516,803,458	401,923,486	306,864,940	115,616,325	191,248,615	66,085,604	125,162,753	85,495,520	82,571,504	12.82	12.39	91.32
			2	596,335,021	495,318,363	402,383,278	317,077,181	98,314,950	218,762,231	68,827,637	149,934,347	92,354,640	75,671,516	13.85	11.35	87.80
			3	694,831,448	600,122,305	457,549,234	357,156,308	122,682,483	234,473,825	72,412,847	162,060,724	30,494,709	29,911,922	9.15	8.97	94.57
			4	662,927,596	566,413,049	455,054,351	361,153,994	120,628,932	240,525,062	77,108,393	163,416,607	83,438,432	79,200,356	12.52	11.88	93.73
			5	722,765,614	594,678,318	504,444,190	394,981,567	120,690,729	274,290,838	118,203,357	156,086,831	36,882,538	36,269,048	11.06	10.88	93.55
			6	569,145,633	492,866,754	413,535,298	328,510,232	111,149,767	217,360,465	62,395,949	154,964,318	89,071,684	85,676,646	13.36	12.85	92.16

Supplementary Table 2. Overview of ST in this study, as well as information for data quantification.

Tissue	Replicates	Raw reads	Reads mapped confidently	Q30 bases	Number of spots	High-quality spots after filtering spots with low counts and few genes	Raw UMI counts per spot	Unique genes per spot	Unique genes per spot after removing genes expressed in fewer than 15 spots	Unique genes per spot after removing genes contaminated by blood contents (highly expressed and related to hemoglobin)	Unique genes per spot after sctransform (SCT)
Psoas major muscle	1	389,591,002	88%	93%	1,854	1,822	1,207	382	370	369	369
	2	603,330,383	88%	92%	2,658	2,607	1,497	449	447	446	446

Supplementary Table 3. Sample information for Hi-C and RNA-seq of adipose tissue in the other 6 species.

Species	Tissue	Replicates	Age	Sex
Human	Abdominal subcutaneous adipose tissue (ASA)	1	Adult, 36-year-old	Male
		2	Adult, 47-year-old	Male
		3	Adult, 20-year-old	Male
Rabbit	Backfat (BF)	1	Adult, 8-month-old	Female
		2		
Rat	Inguinal white adipose tissue (iWAT)	pool 1	Adult, 32-week-old	Female
		pool 2		
Sheep	Abdominal subcutaneous adipose tissue (ASA)	1	Adult, 2-year-old	Female
		2		
		3		
Cat	Subcutaneous adipose tissue on the back (bSAT)	1	Adult, 2-year-old	Female
Dog	Subcutaneous adipose tissue on the back (bSAT)	1	Adult, 2-year-old	Female
		2		

Note: Samples used for Hi-C for rabbit, sheep, cat and dog were the same as those used for comparative transcriptomic analysis as shown in **Supplementary Data 3** (also provided here).

Supplementary Table 4. Hi-C data for adipose tissue in 6 other species.

Species	Tissue	Replicates	Sequenced read pairs	Alignable (normal+ chimeric paired)	Unique reads (after removing PCR duplicates, intrafragment reads and low-mapping-quality reads)	Hi-C contacts	Inter chromosomal	Intra chromosomal	Intra-short range (<20 kb)	Intra-long range (>20 kb)
Human	Abdominal subcutaneous adipose tissue (ASA)	1	503,146,230	429,865,411	380,522,393	307,934,216	97,826,290	210,107,926	69,102,420	141,005,255
		2	586,111,035	501,209,149	439,279,863	357,429,943	102,410,927	255,019,016	81,720,562	173,298,192
		3	498,527,361	433,331,895	332,516,071	265,391,209	82,754,588	182,636,621	56,828,903	125,807,542
Rat	Inguinal white adipose tissue (iWAT)	pool 1	578,421,157	441,490,896	399,870,839	289,113,106	87,439,504	201,673,602	69,160,331	132,513,242
		pool 2	515,328,002	386,451,584	347,304,416	252,275,643	67,287,135	184,988,508	64,303,231	120,685,263
Rabbit	Backfat (BF)	1	561,750,529	476,375,291	345,285,133	259,854,111	59,306,955	200,547,156	101,448,746	99,098,061
		2	502,628,780	404,872,290	328,906,950	243,450,202	69,732,916	173,717,286	66,491,535	107,225,660
Sheep	Abdominal subcutaneous adipose tissue (ASA)	1	532,095,229	421,446,889	343,521,467	245,951,501	61,568,342	184,383,159	70,006,509	114,376,504
		2	597,754,065	469,917,055	386,976,111	279,011,143	68,897,349	210,113,794	82,403,842	127,709,808
		3	506,782,784	407,586,303	313,216,425	220,790,201	58,030,960	162,759,241	57,751,297	105,007,800
Cat	Subcutaneous adipose tissue on the back (bSAT)	1	596,180,999	506,064,080	407,385,391	344,191,217	80,272,687	263,918,530	91,092,885	172,825,506
Dog	Subcutaneous adipose tissue on the back (bSAT)	1	557,314,138	486,814,350	388,087,569	331,129,243	88,576,769	242,552,474	93,666,110	148,886,116
		2	706,446,290	615,893,395	489,792,490	416,561,677	123,668,219	292,893,458	110,553,739	182,339,510

Note: All the Hi-C libraries were sequenced on the Illumina NovaSeq 6000 platform to generate 150 bp paired-end reads.

Supplementary Table 5. RNA-seq data for adipose tissue in 6 other species.

Species	Tissue	Replicates	Raw reads	Clean reads	Raw bases (G)	Clean bases (G)	Q30 (%)
Human	Abdominal subcutaneous adipose tissue (ASA)	1	39,169,633	38,829,488	11.75	11.65	94.94
		2	36,194,367	35,972,847	10.86	10.79	92.20
		3	28,153,925	27,650,038	8.45	8.29	94.77
Rat	Inguinal white adipose tissue (iWAT)	pool 1	34,936,395	34,331,795	10.48	10.30	92.56
		pool 2	33,899,421	33,393,477	10.17	10.02	91.48

Note: RNA-seq data for humans and rats are provided, with RNA-seq data for other species shown in **Supplementary Data 3**. All rRNA-depleted strand-specific RNA-seq was performed on the Illumina HiSeq X Ten platform to generate 150 bp paired-end reads.

Supplementary Table 6. Number of PEIs in each species.

Species	Replicates	Number of PEIs	Enhancers per gene/promoter
Human	1	54,455	3.78
	2	55,133	3.93
	3	56,343	3.81
Rat	1	54,856	3.91
	2	56,746	3.93
Rabbit	1	54,898	4.73
	2	55,649	4.71
Sheep	1	57,817	3.69
	2	56,841	3.70
	3	56,104	3.58
Cat	1	57,507	3.97
Dog	1	55,981	3.86
	2	53,714	3.81
Pig	1	53,148	3.77
	2	55,960	3.89
	3	55,429	3.83
	4	55,710	3.87
	5	42,652	3.39
	6	55,733	3.87

Supplementary Methods

1. Pig transcriptome reconstruction

1.1 Animals

All research involving animals was conducted according to Regulations for the Administration of Affairs Concerning Experimental Animals (Ministry of Science and Technology, China, revised in March 2017), and approved by the animal ethical and welfare committee (AEWC) of Sichuan Agricultural University under permit No. DKY-B20171902. The animals were allowed access to feed and water *ad libitum* and were humanely killed as necessary to ameliorate suffering and were not fed the night before they were slaughtered.

1.2 Sample collection

A total of 194 samples from 70 tissue types (1-3 biological replicates for each of 17 solid organs, as well as 47 skeletal muscles and 6 adipose depots from different body sites) and two immortalized cell lines (kidney epithelial cells [PK15] and iliac endothelial cells [PIECs]) were used in this study. More specifically, most of these samples were collected from three adult, 2-year-old, female Rongchang pigs (a fatty, indigenous Chinese breed, white in colour, with black spots on the head, and drooped ears), while the testes were from three 2-year-old, male Large White pigs (a lean, commercial European breed, white in colour with erect ears), and retinal tissues were derived from two 2-day-old, female, cross-bred Meishan (father; a fatty, indigenous Chinese breed, black in colour with drooped ears) × Tibetan (mother; an indigenous Chinese breed, black in colour with erect ears) pigs. None of the pigs had direct or collateral blood relationships within the last 3 generations.

All the tissue samples were immediately frozen in liquid nitrogen and stored at −80°C until RNA extraction. Two cell lines, PK15 and PIEC, were obtained from the China Infrastructure of Cell Line Resources and Stem Cell Bank of the Chinese Academy of Sciences, respectively. All sample information is provided in detail in **Supplementary Data 1**.

1.3 Construction of rRNA-depleted RNA-seq libraries and data processing

Total RNA was extracted using the RNeasy Mini Kit (Qiagen). We used an rRNA depletion protocol (Ribo-Zero kit, Epicentre) coupled with the Illumina TruSeq stranded RNA-seq library protocol to construct the RNA-seq libraries. All libraries were quantified using the Qubit dsDNA High Sensitivity Assay Kit (Invitrogen) and sequenced on the HiSeq X Ten (Illumina) platform to produce an average of ~49 million 150 bp paired-end raw reads and ~48 million high-quality reads for each library.

Sequence reads were aligned to the pig reference genome (Sscrofa 11.1, GCA_000003025.6) by the STAR alignment tool (version 2.5.3a)[9]. On average, ~96% of reads of individual libraries could be aligned to the pig reference genome, generating an average of ~85.76 million aligned reads for each sample.

1.4 Pig transcriptome *de novo* assembly

For unbiased representative construction of the pig transcriptome, 33 tissues/cell lines (87 samples), *i.e.,* the core atlas dataset, were chosen for *de novo* assembly. The aligned reads of these samples were assembled using Cufflinks (version 2.1.1)[10]. We developed and employed previously reported computational methods AssemblyLine (version 0.2.0) to filter out library-specific background noise and to predict the most likely isoforms from the transcript fragment assemblies (transfrags)[11]. First, transcripts with very short first or last exons (<15 bp) were clipped. Short transfrags with lengths ≤250 bp were discarded as poorly assembled transcripts. Second, the remaining transfrags were classified as 'annotated' or 'unannotated' based on their overlap with annotation in the reference transcriptome. Annotated transfrags and unannotated multi-exonic transfrags were regarded as reliable transcripts and directly retained. Unannotated mono-exonic sense intronic transfrags were discarded as incompletely processed RNA artifacts. Other unannotated mono-exonic transfrags were subjected to a bivariate kernel density classifier to discriminate recurrent, reliable transcripts from genomic DNA contamination artifacts. After filtering, we obtained high-quality assemblies containing reliable transcripts. These high-quality transcript assemblies were then subjected to TACO[12] (a meta-assembly method for transcript structure prediction, version 0.7.3) prior to the construction of transcriptome maps.

1.5 Coding potential assessment and lncRNA and TUCP identification

To facilitate further study of the pig transcriptome, we assessed the coding potential of putative non-coding transcripts that were not annotated as PCGs in the pig reference genome using a stringent filtering pipeline and subsequent classification into long noncoding RNAs (lncRNAs) and transcripts of unknown coding potential (TUCPs).

We predicted coding potential by integrating two sources of evidence: (i) predictions from the alignment-free Coding Potential Calculator (CPC2) (version 0.1)[13], which determines the coding probability of transcript sequences using a support vector machine (SVM) model built from Fickett TESTCODE score, open reading frame (ORF) length, ORF integrity, and isoelectric point data, and (ii) all transcripts with Pfam A (database version 28) domain matches were detected across the three possible translated reading frames using PfamScan (version 1.5). Open reading frames were obtained utilizing EMBOSS (version 6.5.7). Bedtools (version 2.27.1) was used to obtain valid coding Pfam domains.

Transcripts without coding potential (CPC score <0 and without Pfam domain hits) were defined as lncRNAs. LncRNAs were classified into locus biotypes based on both the transcription localization and transcription direction of proximal PCG loci (5-kb distance cut-off) with FEElnc software (version 0.1.1)[14].

Transcripts with *in silico* evidence of coding potential with either a positive CPC2 score or a Pfam domain were designated TUCPs, as originally described by Cabili *et al*[15]. Furthermore, to control for false positives of Pfam domains in TUCP identification, we also scanned coding regions and number- and length-matched non-transcribed intergenic regions in the same manner[11]. We observed 95,345 hits to 5,950 unique Pfam domains in coding regions compared with 6,715 hits to 216 unique domains in non-transcribed intergenic spaces. We compared the occurrences of each Pfam domain in coding versus intergenic regions using Fisher's exact test and flagged 151 domains with an odds ratio of less than 10.0 or P value greater than 0.05 as likely artifacts. The remaining 5,799 Pfam domains were considered valid. This procedure filtered 7,867 artifact hits, retaining 87,478 valid hits, and putative non-transcribed intergenic spaces harbored only 24 (0.34%) of the valid Pfam domains.

1.6 circRNA identification

CIRCExplorer2 (version 2.3.2)[16] was used to retrieve RNA-seq reads that were mapped to back-spliced junction sites for circRNA prediction. Briefly, when sequence reads were aligned to the pig reference genome by the STAR alignment tool[9] (in '*Construction of rRNA-depleted RNA-seq libraries and data processing*'), we used the parameter '--chimSegmentMin 10' to acquire files for further circRNA prediction. Alternative back-splicing was then analyzed using CIRCExplorer2 (version 2.3.2) (http://circexplorer2.readthedocs.io/) with default parameters. Putative circRNAs were required to have more than two independent junction-spanning reads.

1.7 Small RNA library sequencing and miRNA annotation

All libraries were conducted following the TruSeq Small RNA Library Prep Kit protocol and sequenced on the HiSeq 2500 (Illumina) or BGISEQ-500 (BGI) platform. Sequencing yielded an average of ~13.4 million 50 bp single-end raw reads and ~11.91 million high-quality reads (after removing low-quality reads, repeated sequences, and adaptor sequences) for each library. High-quality reads were then mapped to the corresponding reference genome with stringent criteria (0 mismatches over the whole length) using Bowtie (version 1.3.0)[17]. Mappable reads were submitted to miRDeep (version 2.0.0.7)[18] to detect miRNAs with default parameters. Annotated, mature miRNA sequences from pig and all other mammalian and avian species in miRbase[19] (release 22) were used as references. Read counts were normalized by the total count of mappable reads of each sample, also known as reads per million (RPM) or transcripts per million (TPM), for unbiased comparisons among samples.

1.8 Gene expression quantification

We combined the assembled lncRNA and TUCP transcripts with all the annotated PCGs in the reference genome to form comprehensive gene inventories, which were used for subsequent quantification.

Gene expression levels were estimated as TPM using the high-speed transcript quantification tool Kallisto (version 0.43.0)[20], which is a transcriptome-based quantification tool that avoids the considerable bias introduced by the genome alignment step. Detected/transcribed genes (long

transcripts including PCGs, TUCPs, and lncRNAs) were indicated by expression values greater than 0.1 TPM (TPM >0.1) in at least one sample. For miRNAs and circRNAs, we used expression cut-offs of 1 TPM and 0.05 TPM in at least one sample, respectively.

1.9 Transcriptome complexity analysis

Transcriptome complexity was reflected by the fraction of total RNAs accounting for the most highly expressed genes, as determined by calculating the average contribution of each gene to the total transcriptional output of a tissue. For each tissue, the average expression values were sorted in decreasing order. Average expression values for each gene were divided by total gene expression. This value indicated the contribution of each gene to the overall transcription in this tissue.

Lower transcriptome complexity indicated that fewer genes contributed to a large fraction of the total transcriptional output, whereas high transcriptome complexity represented many genes equally contributing to the total transcriptional output.

To account for the unequal numbers of total expressed genes among distinct transcript types, complexity was calculated separately for the five types of transcripts, with the top 0.5% most highly expressed genes shown for comparison.

2. 3D genome structure and chromatin information annotation for each transcript

2.1 Samples

The representative upper layer of backfat (ULB) was collected from six adult 2-year-old female Bama pigs. Sample information is provided in detail in **Supplementary Table 1**.

2.2 *In situ* Hi-C protocol

Hi-C libraries from tissues were generated according to the previously published Hi-C protocol with some minor modifications[21].

Briefly, 1.0 g of adipose tissue was pulverized, and 37.0% formaldehyde was

added to obtain a final concentration of 4.0% for chromatin cross-linking. Mixtures were incubated at room temperature (20–25°C) for 30 min, and glycine was added to obtain a final concentration of 0.25 mol/L to quench the formaldehyde. The mixture was then centrifuged at 1,500×g for 10 min at room temperature. The sediment was added to the lysis buffer and homogenized. The homogenate was centrifuged at 5,000×g for adipocyte sedimentation. Nuclei of formaldehyde-fixed adipose tissue were permeabilized, and DNA was digested with 200 units of MboI (a 4-cutter restriction enzyme) for one hour at 37°C. The restriction fragment overhangs were filled and labeled by biotinylated nucleotides and then ligated in a small volume. After cross-link reversal, the ligated DNA was purified and sheared to a length of 300–500 bp, at which point ligation junctions were pulled down with streptavidin beads and prepped for Illumina NovaSeq 6000 sequencing.

2.3 Hi-C data processing and analysis

Hi-C datasets were processed using a custom pipeline in Juicer software (version 1.8.9, https://github.com/aidenlab/juicer/wiki)[22], which is an efficient open-source tool for analyzing Hi-C datasets. Briefly, high-quality Hi-C reads were aligned to the pig (Sscrofa11.1) reference genome using BWA software (version 0.7.15, http://bio-bwa.sourceforge.net/)[23] with default parameters. Then, the unalignable read pairs (*i.e.,* with at least one end that could not be successfully aligned) were filtered. The duplicated read removal step was accomplished by a parallelized processing awk script packaged in Juicer. Low-quality alignments defined as those in which one or both reads failed to meet the threshold (MAPQ ≥ 30) were removed. Detailed information on the number of Hi-C sequencing reads kept in each processing step in Juicer is provided in **Supplementary Table 4**. Finally, normalized contract matrices were separately generated at three resolutions (500 kb, 100 kb, and 20 kb) using the KR algorithm[22].

2.4 Features of the pig genome

We binned the genome into 100 kb windows and estimated the GC content of each bin by dividing the number of G or C bases in the forward strand by the non-gap base length of the bin. We further calculated CpG content in a similar

way, by dividing the number of CG dinucleotides in the forward strand by the non-gap base length.

2.5 Compartment A/B identification

First, principal component analysis (PCA) was performed as previously described[24] to generate PC1 vectors at a 100 kb resolution. The first two principal components were obtained by using the ‘prcomp’ function in R on the o/e contact matrix. The transcriptional start site (TSS) of each gene was defined as the initial position of the gene model, and gene density was then calculated as the number of TSSs in each bin. Bins with positive Spearman’s correlations between PC1 values and gene density were defined as compartment A; otherwise, they were defined as compartment B.

Then, the A-B index, which represented the likelihood of a sequence interacting with A or B, was created at a 20 kb resolution, as described previously[25]. Bins at 20 kb that associated more with compartment A at 100 kb (higher o/e normalized interaction frequency for compartment A than for compartment B) were considered to be in compartment A, and vice versa for compartment B.

2.6 Identification of topologically associated domains

Based on 20 kb normalized contract matrices, TADs were identified following a previously described procedure that uses a directionality index (DI) metric, which quantifies the degree of upstream or downstream bias for a given bin[26]. A hidden Markov model (HMM) was then used to predict DI states. Domains and boundaries were inferred from the results of the HMM state calls throughout the genome. The topological boundaries were less than 400 kb, and unorganized chromatin was larger than 400 kb.

2.7 Chromatin 3D modeling

MiniMDS (https://github.com/seqcode/miniMDS), a fast, accurate, and memory-efficient method for inferring the genome at high resolution, was used to infer 3D genome structures from the normalized contact matrix at a 100 kb resolution[27]. PyMOL (version 2.3.2) was used to visualize 3D coordinates.

2.8 Gene co-expression associated with TADs

To determine whether genes in the same TAD had a higher probability of being co-expressed, we calculated Spearman's correlations for all neighboring genes on a chromosome. We selected gene pairs with a correlation coefficient above a certain threshold ($r > 0.5$) and compared them to the background. We estimated the fraction of gene pairs residing in the same TAD against all the neighboring gene pairs. We stratified the neighboring gene pairs into groups of different distances, where d=0 represents directly neighboring genes, d=1 represents gene pairs separated by one gene, and so on. These analyses were performed separately using six biological replicates.

3. Gene transcriptional profiling across tissues

3.1 Tissue clustering

We explored the similarity in gene transcription for different transcripts between tissues and across samples by performing hierarchical clustering based on all the transcribed genes. TPM values were used on a $\log_2$-transformed ($\log_2(1+\text{tpm})$) scale. Distance between samples was defined as distance = 1 – correlation. Pearson's correlation was used as the correlation measure.

3.2 Differential gene expression analysis across tissues

Differential gene expression analysis was performed using edgeR (version 3.22.5)[28]. We used a false discovery rate (FDR) ≤ 0.05 and $\log_2$(fold change) ≥ 1 as cut-offs for statistical significance. All pairwise combinations between all tissues were tested. Dispersion was set to 0.1 for comparison of tissues without biological replicates.

3.3 Tissue-specific expression analysis

We calculated the tissue specificity of gene expression reflected by the tau score (τ) (ranging from 0 to 1, with 1 for highly tissue-specific genes and 0 for ubiquitously transcribed genes) for each gene according to Yanai Itai *et al.*[29] with scaled TPM values. For each tissue, we averaged all replicates and then calculated τ as the inequality among replicates for different tissues. We used $\tau \geq 0.75$ as the cut-off for tissue-specific genes.

3.4 Functional enrichment analysis

Functional enrichment analysis of Gene Ontology (GO) terms and pathways was performed using Metascape (http://metascape.org)[30]. Genes were mapped to their respective human orthologs, and the lists were submitted to Metascape for enrichment analysis in order to identify significant over-representation of GO biological processes (GO-BP) and Kyoto Encyclopedia of Genes and Genomes (KEGG) pathway categories. In all tests, all genes in the genome were used as the enrichment background. Only GO-BP or KEGG pathway terms with resulting FDR-corrected *P* values less than 0.05 were considered significant and are depicted using $-\log_{10}(q)$ bar plots.

4 Spatiotemporal transcriptomics for SMTs

4.1 Spatial transcriptomic (ST) experiments

Collection and preparation of samples

The representative oxidative slow psoas major (PM) muscle was collected from two pigs for ST experiments. Fresh porcine PM tissue was cut into approximately 6.5 mm × 6.5 mm × 1 cm pieces and snap-frozen in liquid nitrogen to avoid crystal formation. Then, tissue samples were embedded at the optimal cutting temperature (OCT) and stored at -80°C. For ST analysis, the samples were sectioned on a cryostat at a thickness of 15 μm, and each section was placed within a capture area on a Visium Spatial slide.

Permeabilization and fluorescent cDNA synthesis

The tissue sections on Visium Spatial slides were permeabilized according to the protocol provided by 10X Genomics. Briefly, the slides were placed in a slide cassette. Permeabilization enzyme was not added to two of the eight wells; 2 μg of RNA was added to the center of one of the wells as the positive control, and the other, empty well served as the negative control. The remaining 6 wells were assigned different permeabilization times of 3, 6, 12, 18, 24, and 30 min. Then, 70 μl of permeabilization enzyme was added to the side of each 30 min well, and the slide was sealed and placed in a thermocycler adaptor at 37°C. Six minutes later, the slide cassette was removed and sealed, 70 μl of

permeabilization enzyme was added to the 24 min well, the seal was reapplied, and the slide was placed in the thermocycler again. This process was repeated until after 27 min, working backwards to the shortest incubation time (3 min). After completion of the time course, the permeabilization enzyme was removed from each well without touching the tissue sections, and 100 μl of 0.1× SSC was added to all wells except that serving as the positive control.

Next, SSC was removed from each well, and 50 μl of Fluorescent RT Master Mix was added to initiate cDNA synthesis. Once the synthesis finished, the Fluorescent RT Master Mix was replaced by 100 μl of 0.1× SSC. Then, the SSC was removed, 70 μl of tissue removal mix was added, and the cells were incubated on a thermocycler adaptor to initiate tissue removal. At the end of incubation, the slide seal and tissue removal mix were removed. The slide was taken out of the slide cassette, immersed in 2× SSC-0.1% SDS at 50°C 15 times, 0.2× SSC at room temperature (RT) 15 times, and 0.1× SSC at RT 15 times, and spin-dried. Finally, the samples were centrifuged at 250 x g for 30 seconds in a slide spinner to ensure that no tissue remained on the slides. Fluorescence images were scanned by a digital slice scanner, and the optimal permeabilization time was selected by combining fluorescence intensity and RNA diffusion degree.

ST library preparation and sequencing

Fragmented and barcoded porcine RNA was used as the carrier material. The spike-in constituted approximately 25% of the libraries. ST cDNA libraries were diluted to 4 nM and sequenced on the Illumina NovaSeq 6000 platform using paired-end sequencing. Samples were sequenced at a mean depth of 50 million paired-end reads, which resulted in an average library saturation above 90%.

4.2 ST sequencing data processing and analysis

Space Ranger analysis

Sample demultiplexing, image alignment, barcode processing, and gene counting were performed by using Space Ranger (version 1.1) according to the 10X Genomics™ spatial gene expression analysis pipelines

(https://support.10xgenomics.com/spatial-gene-expression/software/overview/welcome).

Before running Space Ranger, a customized reference database was made that was suitable for the mapping and gene annotation of pig muscular tissue. We used the reference genome sequence and gene annotation file (GTF) from Ensembl Sscrofa 11.1 release 102 (GCA_000003025.6) (http://asia.ensembl.org/Sus_scrofa/Info/Index?db=core). Y-chromosome-related information was removed (because our samples were from sows). Annotation information for the *MYH2* and *MYH4* marker genes for muscle fiber types IIA and IIB, respectively (from the old Sscrofa11.1 release 90) was added to the GTF file because annotation information for these genes was absent from the Ensembl release 102. We used the "spaceranger mkref" command to complete spatial reference database construction.

Reads were aligned to the Ensemble pig genome and transcriptome annotation references. We used the "spaceranger counts" command for read mapping and gene counting. The H&E-stained photos of tissue sections, slide serials, and capture area information of each sequencing sample were provided in this counting step for automatic fiducial alignment and tissue detection.

After obtaining the first round counting results, Loupe Browser software (10X Genomics^{TM}) was used to quickly visualize the spatial results. We evaluated the biological reliability of all spots in each sample, and unreliable spots were filtered out with the Manual Alignment function (this step will produce the json file of the spot coordinate information after filtering). Finally, only the reliable spots were used to rerun the former counting step, and the count matrix was exported for downstream analysis.

Seurat analysis

We used the Seurat (version 3.2) package (https://satijalab.org/seurat/)[31] in the R environment to conduct a follow-up assessment of the count matrix data of each sample. We loaded spatial data into Seurat using the “Load10X_Spatial” function. These input data were taken directly from the output of the former Space Ranger pipeline.

We performed the data quantification analysis as follows. First, we evaluated the raw data of UMI counts, gene numbers, and mitochondrial gene proportions for each spot. Second, quantification of spots and genes was carried out by using the “subset” function. We filtered out ~2% of low-quality spots. Spots with over 25% mitochondrial gene expression were also discarded (the percentage of total counts belonging to 13 mitochondrial genes for each cell was calculated with the “PercentageFeatureSet” function). Third, genes expressed in fewer than 15 spots were excluded. Genes related to hemoglobin (considerable variation from blood contents) were also removed. The above filtering steps yielded ~1,822 (replicate 1) and 2,607 (replicate 2) spots with ~1,204 (replicate 1) and 1,509 (replicate 2) median UMI counts and ~369 (replicate 1) and 446 (replicate 2) median genes per spot for subsequent analysis. Spot information before and after quality control is summarized in **Supplementary Table 2**.

STC normalization

The cleaned expression matrix data were normalized using regularized negative binomial regression (“SCTransform” function)[32].

PCA

For dimensionality reduction, PCA was performed, and the 10 most significant components were determined with the “DimHeatmap” and “ElbowPlot” functions (a heuristic method).

Spot clustering, differential gene expression analysis, and myofiber identification

In the spot clustering process, the shared nearest neighbor (SNN) graph construction method (“FindNeighbors” function) was used, and clusters were identified with the “FindClusters” function. For resolution setting, based on the known biological characteristics of muscle tissue as a logical guide, we specifically adjusted the resolution parameters of each sample to control the number of clusters within a reasonable range. We used UMAP to visualize spot clusters in a reduced 2D space (“RunUMAP” and “DimPlot” functions). To evaluate the reliability of clustering, we generated an expression abundance heat map (“FeaturePlot” function) for the *MYH7*, *MYH4,* and *MYH2* marker

genes for muscle fiber types I, 2B, and 2A, respectively, with UMAP. Preliminary characterization of the muscle fiber type of clusters was performed by studying the expression levels of these three genes in each cluster. We identified differentially expressed genes between clusters using the “FindAllMarkers” function (settings: test.use= wilcox, min.pct = 0.1, logfc.threshold: = 0.2).

According to the above steps for clustering annotation, we first identified the type I and II muscle fiber clusters **(Supplementary Fig. 21)** and then distinguished between type IIA and IIB muscle fiber clusters **(Supplementary Fig. 22)**. In addition, we used the “SpatialPlot” function to visualize the spatial distribution of each cluster (**Fig. 4b and 4c**). Then, spatial distribution maps of each sample were compared with the previous ATPase staining photos for the same sample to further confirm the classification of the clusters.

Selection of representative spots for three types of myofiber clusters

Myofiber distribution exhibited a non-random pattern, that is, the same type of myofibers generally gathered together spatially. In light of this phenomenon, we developed a local homogeneity (LH) score for each spot (reflected by the number of each type of six spots surrounding a given central spot) to assess the homogeneity of spots (*i.e.*, to ensure that the gene expression profile for a given spot was obtained from a homogeneous set of myofibers) **(Supplementary Fig. 23a).**

Using type I muscle fibers as an example, if the 6 adjacent spots all belong to type I, then the LH value of the center spot is calculated as 6, indicating a high probability that the central spot is also a type I myofiber due to the non-random pattern of homogeneous myofiber distribution.

According to this principle, we divided the three types of myofiber spots into 6 groups from LH 6 to 1. To verify the accuracy of the LH scoring, we compared the spatial position results of different LH groups with the results of ATPase-stained images **(Supplementary Fig. 23b)**. Spots with different LH scores were then mapped to their corresponding positions on the spatial slide using the SpatialDimPlot() function in Seurat. Then, the spots of each LH group were projected over an ATPase-stained image of the same region of the sample using an image mask. Moreover, we also investigated changes in the

expression of the myofiber marker genes (*MYH7*, *MYH2,* and *MYH4*) among the different LH groups **(Supplementary Fig. 23c)**.

To accurately obtain the specific expression signatures of the three muscle fiber types, we extracted the top 200 spots with the highest LH scores for each type of myofiber, and used these as the most representative spots (*i.e.,* spots with highest possibility of accurately representing each type of myofiber) **(Supplementary Fig. 24)**.

4.3 Estimation of the myofiber proportions in bulk RNA-seq data

We sought to estimate the myofiber proportions using bulk RNA-seq data for different types of muscles derived from distinct body parts, in combination with spatial transcriptomic data for PM muscle with CIBERSORTx[33]. In brief, a signature matrix was generated based on spatial transcriptome TPM values of representative spots for the three myofiber cluster types (I, IIA, and IIB). Beyond the well-known myofiber-specific markers *MYH7*, *MYH2,* and *MYH4*, this matrix also included several genes, such as *MYOM3* of type I, *AKT3* of type IIA, and *MED1* (involved in glucose metabolism; knockout of this gene leads to a fast-to-slow myofiber switch) of type IIB.

The default parameters were applied, except that the analysis mode, q-value, replicate number, and minimum expression level were set to single cell, 0.05, 20, and 0, respectively. The myofiber proportions were further measured by deconvoluting the gene expression levels (also in TPM) in the bulk RNA-seq data based on the signature matrix obtained above using S-mode batch effect correction.

4.4 Verification of the myofiber proportions using ATPase staining

ATPase staining distinguished type I (dark staining) from type II (light staining) fibers. The staining process was as follows: The tissue sections were first treated with acid pre-incubation solution (pH 4.2~4.6) for 5 min, which contained 5 ml of barbiturate sodium acetate, 10 ml of 0.1 mol/L HCl and 8 ml of ddH_2O. The treated tissue sections were then transferred to alkaline pre-incubation solution (2 ml of 0.1 mol/L barbiturate sodium, 2 ml of 0.18 mol/L $CaCl_2$, and 6 ml of ddH_2O, adjusted to pH 10.4 with 0.1 mol/L NaOH) for 5 min.

Then, the slides were immersed in 4 ml of 0.1 mol/L barbiturate sodium, 2 ml of 0.18 mol/L $CaCl_2$, 50 mg of $ATPaseNa_2$, and 14 ml of ddH_2O (pH 9.4~9.7) for 30 min. The slides were washed with 1% $CaCl_2$ 3 times, 2% CoCl2 for 3 min, and 0.01 mol/L barbiturate sodium 8 times and then rinsed in ddH_2O thoroughly. Thereafter, the cells were incubated with 1% $(NH_4)_2S$ for 1 min, washed with tap water, dehydrated with ethanol and finally sealed.

5 Comparative transcriptomic analysis

5.1 Single-copy orthologous PCG identification

Single-copy orthologous protein-coding gene (PCG) families were identified following a protocol similar to the one recommended by Ensembl:

http://asia.ensembl.org/info/genome/compara/homology_method.html. In brief, first, the longest protein-coding translation was extracted for each gene of each species, then all-against-all blast of these translations was performed between self and nonself-species; second, based on the blast results, a sparse graph was built, and clusters were extracted using hclust_sg; third, large clusters with over 400 genes were split into smaller ones recursively until no cluster with 400 genes was retained; fourth, for each cluster, multiple alignments of protein-coding sequences were constructed and further back-translated to coding sequence (CDS) alignments; and fifth, a phylogenetic tree was built, and single-copy orthologous gene families were identified.

5.2 Analyses of alternative splicing

Each internal exon in every annotated transcript was taken as a “cassette” exon. Each alternative splicing (AS) “cassette” was composed of 3 exons, C1, A, and C2, where A is the alternative exon, C1 is the 5’ alternative exon, and C2 is the 3’ alternative exon. Using the read alignment files, we extracted and counted reads that were split and mapped to multiple exons; in particular, we counted reads mapped to both C1 and A (#C1A), both C1 and C2 (#C1C2), and both A and C2 (#AC2). The “percent-spliced in” (PSI) values for each internal exon were defined as PSI = 100 × average (#C1A, #AC2)/(#C1C2 + average (#C1A, #AC2)); here, #C1A, #AC2, and #C1C2 are the normalized read counts for the associated junctions. Orthologous exons for cross-species comparative

analyses of AS were determined by converting the genomic coordinates between genomes using the liftOver tool and chain files from the UCSC genome browser and choosing 1:1 overlaps. For cross-species analyses, we included exons with single-copy orthologs in all species and PSI values in all samples.

5.3 Characterizing evolutionary gene transcription patterns

Species trees along with their topologies and branch lengths were retrieved from TimeTree[34]. We performed selection analysis of gene transcriptional changes, as previously reported[35]. In brief, we fitted the cross-species expression data for each tissue and the species tree to a single optimum Ornstein-Uhlenbeck (OU) model (using the R package Geiger) and to BM1 (single-rate Brownian)/BM2 (multirate Brownian) models (using the brownie.lite() function in the R package phytools)[36]. Genes that underwent selection of expression were defined as those with a change in the Akaike information criterion (ΔAIC) (the AIC value of the BM1 or OU model minus that of the BM2 model, whichever was smaller) greater than 7, a relatively stringent cut-off suggested by Daniel *et al.*[35].

6. Gene transcription divergence and PEIs across species

6.1 Samples

We chose adipose tissue to investigate the divergence in gene expression driven by PEIs across seven species, including human, rat, rabbit, cat, dog, pig and sheep. Briefly, we obtained Hi-C data for the above species (see **Supplementary Table 3 and 4** for more details) according to the experimental and analytical procedures described in '***Hi-C experiment and data analysis***'. High-quality Hi-C reads were separately aligned to the pig (Sscrofa11.1), human (GRCh38), rat (Rnor_6.0), rabbit (OryCun2.0), sheep (Oar_v3.1), dog (CanFam3.1), and cat (Felis_catus_6.2) reference genomes using BWA software (V0.7.15, http://bio-bwa.sourceforge.net/) with default parameters.

Collection and sequencing of human clinical samples were approved by the Ethics Committee of Sichuan Provincial People's Hospital, and informed consent was obtained before the study.

6.2 Identification of promoter-enhancer interactions (PEIs)

We identified PEIs by applying PSYCHIC[37] at 20 kb resolution. In brief, the genome was segmented into topological domains, and similar neighboring domains were further merged into hierarchical structures. Then, a bilinear power-law model was fitted for each TAD or merged to build a domain-specific background model. Over-represented promoter-enhancer pairs were identified using interaction intensity normalized by the background model.

6.3 Evolutionary divergence in gene transcription driven by PEIs

Evolutionary divergence of gene transcription was measured by Spearman's correlation coefficients for the transcription levels of orthologous genes between pairs of species.

When estimating the relative divergence of genes with different numbers of interacting enhancers, confounding effects due to differences in the distribution of transcription level were controlled for by matching genes one-to-one to control genes with similar transcription levels. Each gene set of interest was matched to a distinct set of controls in R with the MatchIt library[38] using the caliper option to prune genes that could not be matched with an appropriate control (caliper of 0.001).

Transcribed genes (mean expression > 1 transcript per million across species; 6,693 genes) were also classified into evolutionarily stable or variable genes based on their CV (standard deviation across species normalized by mean transcription; bottom 50%: stable; top 50%: variable). As these two categories had different mean transcription levels (15.61 versus 24.65 TPM, respectively; Wilcoxon rank sum test: $P < 10^{-16}$), we additionally matched stable and variable genes into pairs with similar mean transcription levels across species with MatchIt, as described above (2,207 genes in each group) and removed 2,279 unmatched genes from the subsets.

References

1 Derrien, T. *et al.* The GENCODE v7 catalog of human long noncoding RNAs: analysis of their gene structure, evolution, and expression. *Genome Res.* **22**, 1775-1789 (2012).

2 Warr, A. *et al.* An improved pig reference genome sequence to enable pig genetics and genomics research. *Gigascience* **9**, giaa051 (2020).

3 Tang, Z. L. *et al.* Comprehensive analysis of long non-coding RNAs highlights their spatio-temporal expression patterns and evolutional conservation in *Sus scrofa*. *Sci. Rep.* **7**, 43166 (2017).

4 Yang, Y. L. *et al.* Systematic identification and molecular characteristics of long noncoding RNAs in pig tissues. *Biomed. Res. Int.* **2017**, 6152582 (2017).

5 Kern, C. *et al.* Genome-wide identification of tissue-specific long non-coding RNA in three farm animal species. *BMC Genomics* **19**, 684 (2018).

6 Li, A. *et al.* ALDB: a domestic-animal long noncoding RNA database. *PloS One* **10**, e0124003 (2015).

7 Zhao, Y. *et al.* NONCODE 2016: an informative and valuable data source of long non-coding RNAs. *Nucleic Acids Res.* **44**, D203-208 (2016).

8 Hon, G. C. *et al.* Epigenetic memory at embryonic enhancers identified in DNA methylation maps from adult mouse tissues. *Nat. Genet.* **45**, 1198-1206, (2013).

9 Dobin, A. *et al.* STAR: ultrafast universal RNA-seq aligner. *Bioinformatics* **29**, 15-21, (2013).

10 Trapnell, C. *et al.* Transcript assembly and quantification by RNA-Seq reveals unannotated transcripts and isoform switching during cell differentiation. *Nat. Biotechnol.* **28**, 511-515 (2010).

11 Iyer, M. K. *et al.* The landscape of long noncoding RNAs in the human transcriptome. *Nat. Genet.* **47**, 199-208 (2015).

12 Niknafs, Y. S., Pandian, B., Iyer, H. K., Chinnaiyan, A. M. & Iyer, M. K. TACO produces robust multisample transcriptome assemblies from RNA-seq. *Nat. Methods* **14**, 68-70, (2017).

13 Kang, Y.-J. *et al.* CPC2: a fast and accurate coding potential calculator based on sequence intrinsic features. *Nucleic Acids Res.* **45**, W12-W16 (2017).

14 Wucher, V. *et al.* FEELnc: a tool for long non-coding RNA annotation and its application to the dog transcriptome. *Nucleic Acids Res.* **45**, e57 (2017).

15 Cabili, M. N. *et al.* Integrative annotation of human large intergenic noncoding RNAs reveals global properties and specific subclasses. *Genes Dev.* **25**, 1915-1927 (2011).

16 Zhang, X. O. *et al.* Diverse alternative back-splicing and alternative splicing landscape of circular RNAs. *Genome Res.* **26**, 1277-1287 (2016).

17 Langmead, B. & Salzberg, S. L. Fast gapped-read alignment with Bowtie 2. *Nat. Methods* **9**, 357 (2012).

18 Friedländer, M. R. *et al.* Discovering microRNAs from deep sequencing data using miRDeep. *Nat. Biotechnol.* **26**, 407-415 (2008).

19 Kozomara, A. & Griffiths-Jones, S. miRBase: annotating high confidence microRNAs using deep sequencing data. *Nucleic Acids Res.* **42**, D68-D73 (2014).

20 Bray, N. L., Pimentel, H., Melsted, P. & Pachter, L. Near-optimal probabilistic RNA-seq

quantification. *Nat. Biotechnol.* **34**, 525-527 (2016).
21 Rao, S. S. *et al.* A 3D map of the human genome at kilobase resolution reveals principles of chromatin looping. *Cell* **159**, 1665-1680 (2014).
22 Durand, N. C. *et al.* Juicer provides a one-click system for analyzing loop-resolution Hi-C experiments. *Cell Syst.* **3**, 95-98 (2016).
23 Li, H. & Durbin, R. Fast and accurate long-read alignment with Burrows–Wheeler transform. *Bioinformatics* **26**, 589-595 (2010).
24 Lieberman-Aiden, E. *et al.* Comprehensive mapping of long-range interactions reveals folding principles of the human genome. *Science* **326**, 289-293 (2009).
25 Jordan, R. M., Nichols Michael, H., Xiaowen, L. & Masami, A.-K. Rivera I. Sarahi M., Hermetz Karen, Wang Ping, Ruan Yijun, Corces Victor G. Evolutionarily conserved principles predict 3D chromatin organization. *Mol. Cell* **67**, 837-852 (2017).
26 Dixon, J. R. *et al.* Topological domains in mammalian genomes identified by analysis of chromatin interactions. *Nature* **485**, 376-380 (2012).
27 Rieber, L. & Mahony, S. miniMDS: 3D structural inference from high-resolution Hi-C data. *Bioinformatics* **33**, i261-i266 (2017).
28 Robinson, M. D., McCarthy, D. J. & Smyth, G. K. edgeR: a Bioconductor package for differential expression analysis of digital gene expression data. *Bioinformatics* **26**, 139-140 (2010).
29 Yanai, I. *et al.* Genome-wide midrange transcription profiles reveal expression level relationships in human tissue specification. *Bioinformatics* **21**, 650-659 (2005).
30 Zhou, Y. *et al.* Metascape provides a biologist-oriented resource for the analysis of systems-level datasets. *Nat. Commun.* **10**, 1-10 (2019).
31 Stuart, T. *et al.* Comprehensive integration of single-cell data. *Cell* **177**, 1888-1902. e21 (2019).
32 Hafemeister, C. & Satija, R. Normalization and variance stabilization of single-cell RNA-seq data using regularized negative binomial regression. *Genome Biol.* **20**, 1-15 (2019).
33 Newman, A. M. *et al.* Determining cell type abundance and expression from bulk tissues with digital cytometry. *Nat. Biotechnol.* **37**, 773-782 (2019).
34 Hedges, S. B., Marin, J., Suleski, M., Paymer, M. & Kumar, S. Tree of life reveals clock-like speciation and diversification. *Mol. Biol. Evol.* **32**, 835-845 (2015).
35 Koenig, D. *et al.* Comparative transcriptomics reveals patterns of selection in domesticated and wild tomato. *Proc. Natl Acad. Sci. USA* **110**, E2655-E2662 (2013).
36 O'Meara, B. C., Ané, C., Sanderson, M. J. & Wainwright, P. C. Testing for different rates of continuous trait evolution using likelihood. *Evolution* **60**, 922-933 (2006).
37 Ron, G., Globerson, Y., Moran, D. & Kaplan, T. Promoter-enhancer interactions identified from Hi-C data using probabilistic models and hierarchical topological domains. *Nat. Commun.* **8**, 2237 (2017).
38 Stuart, E. A., King, G., Imai, K. & Ho, D. MatchIt: nonparametric preprocessing for parametric causal inference. *J. Stat. Softw.* (2011).
