# Supplementary material for: A pig BodyMap transcriptome reveals diverse tissue physiologies and evolutionary dynamics of transcription
Source: Nat Commun. 2021 Jun 17;12:3715. doi: 10.1038/s41467-021-23560-8 (PMC8211698; doi:10.1038/s41467-021-23560-8)
Supplement: Supplementary file 3 — Description of Additional Supplementary Files [file 41467_2021_23560_MOESM3_ESM.docx]

File name: Supplementary Data 1.

Description: **Pig samples used in this study.**

File name: Supplementary Data 2.

Description: **Summary of all RNA-seq data in pig.**

File name: Supplementary Data 3.

Description: **Summary of RNA-seq data in other species.**

File name: Supplementary Data 4.

Description: **Chromatin information annotated for transcripts (PCGs, TUCPs and lncRNAs).**

File name: Supplementary Data 5.

Description: **Transcripts (PCGs, TUCPs, lncRNAs, circRNAs and miRNAs) specifically correlated with *MYH*s.** Pearson’s correlation coefficient between each transcript and *MYH* was calculated with two-sided statistical significance of the *P* value.

File name: Supplementary Data 6.

Description: **Signature matrix for different myofibers.**

File name: Supplementary Data 7.

Description: **Functional enrichment of genes with significant transcriptional switching for each species and tissue.** *P* values are calculated based on a one-sided cumulative hypergeometric test without adjustment, and are shown as log10(*P*-value).
