# Supplementary material for: A pig BodyMap transcriptome reveals diverse tissue physiologies and evolutionary dynamics of transcription
Source: Nat Commun. 2021 Jun 17;12:3715. doi: 10.1038/s41467-021-23560-8 (PMC8211698; doi:10.1038/s41467-021-23560-8)
Supplement: Supplementary file 11 — Reporting Summary [file 41467_2021_23560_MOESM11_ESM.pdf]

Corresponding author(s): Mingzhou Li; Qianzi Tang
Last updated by author(s): Apr 19, 2021

Reporting Summary

Nature Research wishes to improve the reproducibility of the work that we publish. This form provides structure for consistency and transparency in reporting. For further information on Nature Research policies, see our Editorial Policies and the Editorial Policy Checklist.

Statistics

For all statistical analyses, confirm that the following items are present in the figure legend, table legend, main text, or Methods section.

n/a	Confirmed	
☐	☒	The exact sample size (*n*) for each experimental group/condition, given as a discrete number and unit of measurement
☐	☒	A statement on whether measurements were taken from distinct samples or whether the same sample was measured repeatedly
☐	☒	The statistical test(s) used AND whether they are one- or two-sided<br>*Only common tests should be described solely by name; describe more complex techniques in the Methods section.*
☐	☒	A description of all covariates tested
☐	☒	A description of any assumptions or corrections, such as tests of normality and adjustment for multiple comparisons
☐	☒	A full description of the statistical parameters including central tendency (e.g. means) or other basic estimates (e.g. regression coefficient) AND variation (e.g. standard deviation) or associated estimates of uncertainty (e.g. confidence intervals)
☐	☒	For null hypothesis testing, the test statistic (e.g. F, t, r) with confidence intervals, effect sizes, degrees of freedom and P value noted<br>*Give P values as exact values whenever suitable.*
☒	☐	For Bayesian analysis, information on the choice of priors and Markov chain Monte Carlo settings
☒	☐	For hierarchical and complex designs, identification of the appropriate level for tests and full reporting of outcomes
☒	☐	Estimates of effect sizes (e.g. Cohen's *d*, Pearson's *r*), indicating how they were calculated

Our web collection on statistics for biologists contains articles on many of the points above.

Software and code

Policy information about availability of computer code

Data collection	The nuclear PCG entries with mitochondrial localization were downloaded from MitoCarta2.0 database (http://www.broadinstitute.org/pubs/MitoCarta).
Data analysis	Bulk RNA-Seq reads were aligned to the respective reference genome using STAR (v2.5.3a). Transcriptome de novo assemblies were generated using cufflinks (v2.2.1). AssemblyLine (v 0.2.0) was applied to filter background transcripts. TACO (v0.7.3) was used to merge transcripts and compare them to known transcripts. Open reading frames were obtained utilizing EMBOSS (v6.5.7). Coding potential of the transcripts were calculated using CPC2 (v0.1). PfamScan (v1.5) was applied to validate coding region pfam domains. Bedtools (v2.27.1) and custom codes (valid_coding_pfam_domains.py deposited under https://github.com/QianZiTang) were combined to obtain valid coding pfam domains. Final lncRNA sets were obtained by custom codes (get_gtf_by_id.py deposited under https://github.com/QianZiTang). FEElnc software (v 0.1.1) was used to classify lncRNAs. circRNAs were identified using CIRCexplorer2 (v2.3.2). Gene-level expression was estimated as TPM using the high-speed transcript quantification tool Kallisto (v0.43.0). Differential gene expression analysis was performed using edgeR (v3.22.5). Small RNA library sequencing data was processed and miRNA annotation was performed using mirdeep (version 2.0.0.7) and bowtie (v1.3.0). High-quality Hi-C reads were aligned to the reference genome using BWA software (v0.7.15). Juicer (v1.8.9) was applied to filter aligned reads and generate normalized contact matrices. Compartment A/B was identified using principal components analysis (using R v3.4) followed by the AB index method (using custom codes deposited under https://github.com/QianZiTang). Promoter-enhancer interactions were identified using PSYCHIC (2018-01-05). 3D structure of the genome was inferred using MiniMDS (2018-09-27) and PyMOL (v2.3.2) was used to visualize 3D coordinates. Spatial transcriptomic data was processed using Seurat (v3.2). All codes and scripts are available on https://github.com/QianZiTang/.

For manuscripts utilizing custom algorithms or software that are central to the research but not yet described in published literature, software must be made available to editors and reviewers. We strongly encourage code deposition in a community repository (e.g. GitHub). See the Nature Research guidelines for submitting code & software for further information.

Data

Policy information about availability of data

All manuscripts must include a data availability statement. This statement should provide the following information, where applicable:

- Accession codes, unique identifiers, or web links for publicly available datasets
- A list of figures that have associated raw data
- A description of any restrictions on data availability

The authors declare that all data supporting the findings of this study are available within the article and its Supplementary Information files or from the corresponding author upon reasonable request.
Raw and processed RNA, and miRNA sequencing data of pigs have been deposited in the NCBI Gene Expression Omnibus (GEO) under accession codes GSE162145 (https://www.ncbi.nlm.nih.gov/geo/query/acc.cgi?acc=GSE162145) and GSE162147 (https://www.ncbi.nlm.nih.gov/geo/query/acc.cgi?acc=GSE162147), respectively. Spatial transcriptomic data have been deposited in GEO under accession code GSE161882 (https://www.ncbi.nlm.nih.gov/geo/query/acc.cgi?acc=GSE161882). The raw and processed RNA sequencing data of nine other species for comparative transcriptomic analysis have been deposited in GEO under accession code GSE162142 (https://www.ncbi.nlm.nih.gov/geo/query/acc.cgi?acc=GSE162142). RNA-sequencing and Hi-C data of non-human species for analysis of gene transcription divergence and PEIs across species have been deposited in GEO under accession codes GSE162146 (https://www.ncbi.nlm.nih.gov/geo/query/acc.cgi?acc=GSE162146) and GSE162140 (https://www.ncbi.nlm.nih.gov/geo/query/acc.cgi?acc= GSE162140), respectively. Human RNA sequencing and Hi-C data have been deposited in GEO under accession codes GSE162143 (https://www.ncbi.nlm.nih.gov/geo/query/acc.cgi?acc=GSE162143) and GSE162139 (https://www.ncbi.nlm.nih.gov/geo/query/acc.cgi?acc=GSE162139), respectively. The sequencing data of human have been also deposited in Genome Sequence Archive (GSA) database for human in the National Genomics Data Center of China under accession codes PRJCA003737 (https://bigd.big.ac.cn/gsa-human/s/mSWmEjKN).
Source data are provided with this paper.
The nuclear PCG entries with mitochondrial localization were downloaded from MitoCarta2.0 database (http://www.broadinstitute.org/pubs/MitoCarta).
Source data are provided with this paper.

Field-specific reporting

Please select the one below that is the best fit for your research. If you are not sure, read the appropriate sections before making your selection.

☒ Life sciences ☐ Behavioural & social sciences ☐ Ecological, evolutionary & environmental sciences

For a reference copy of the document with all sections, see nature.com/documents/nr-reporting-summary-flat.pdf

Life sciences study design

All studies must disclose on these points even when the disclosure is negative.

Sample size	No prior sample size determination was conducted. To comprehensively survey the pig transcriptome, a total of 194 samples from 70 tissues (1-3 biological replicates for each of 17 solid tissues, as well as 47 skeletal muscles and 6 adipose depots from different body sites) and two immortalized cell lines (kidney epithelial cells [PK15] and iliac endothelial cells [PIECs]) were used in this study. For reconstruction of 3D genome structures, we performed an in situ Hi-C experiment for a pig subcutaneous adipose tissue with six replicates. We performed spatial transcriptomics using the representative psoas major muscle in two replicates. For comparative transcriptomic analysis across species, we performed three replicates for each species, with exception for mouse, rat and guinea pig with one replicate because of admitted high repeatability. For the integrative analysis of transcription divergence and promoter-enhancer interactions across species, 1-3 replicates were conducted.<br>Statistical testing ensured significant findings.
Data exclusions	No data were excluded from the study.
Replication	Reproducibility between biological replicates was assessed by correlation/PCA/tSNE analysis.<br>All attempts at replication were successful.
Randomization	Not relevant to our study since we did not use settings of experiment/control groups.
Blinding	Not relevant to our study since we did not use settings of experiment/control groups.

Reporting for specific materials, systems and methods

We require information from authors about some types of materials, experimental systems and methods used in many studies. Here, indicate whether each material, system or method listed is relevant to your study. If you are not sure if a list item applies to your research, read the appropriate section before selecting a response.

Materials & experimental systems

n/a	Involved in the study
☒	☐ Antibodies
☐	☒ Eukaryotic cell lines
☒	☐ Palaeontology and archaeology
☐	☒ Animals and other organisms
☐	☒ Human research participants
☒	☐ Clinical data
☒	☐ Dual use research of concern

Methods

n/a	Involved in the study
☒	☐ ChIP-seq
☒	☐ Flow cytometry
☒	☐ MRI-based neuroimaging

Eukaryotic cell lines

Policy information about cell lines

Cell line source(s)	Iliac endothelial cells [PIECs] was purchased from Shanghai Cell Bank of Type Culture Collection of Chinese Academy of Sciences (Shanghai, China);<br>Kidney epithelial cells [PK15] was purchased from Kunming Cell Bank of Type Culture Collection of Chinese Academy of Sciences (Kunming, China).
Authentication	All the cell lines were authenticated by the respective Cell Bank, based on the monitoring of morphological features and isoenzyme electrophoretic analysis.
Mycoplasma contamination	No mycoplasma contamination was confirmed.
Commonly misidentified lines (See ICLAC register)	No misidentified cell lines were used.

Animals and other organisms

Policy information about studies involving animals; ARRIVE guidelines recommended for reporting animal research

Laboratory animals	Pig (Rongchang pigs, female, 2-year-old)<br>Pig (Large white pigs, male, 2-year-old)<br>Pig (Bama pigs, female, 2-year-old)<br>Pig (crossbred Meishan (father) × Tibetan (mother) pigs, female, 2-day-old)<br>Monkey (Rhesus, female, 5-year-old)<br>Mouse (C57BL/6J, female, 12-week-old)<br>Rat (SD rat, female, 6-month-old)<br>Guinea pig (Guinea pig, female, 8-month-old)<br>Rabbit (Japanese big-ear white rabbit, female, 8-month-old)<br>Cat (A native breed of cat from Chengdu city, female, 2-year-old)<br>Dog (Beagle dog, female, 2-year-old)<br>Sheep (Small-tail Han sheep, female, 2-year-old)<br>Chicken (Tibetan chicken, female, 10-month-old)
Wild animals	No wild animals used in the study.
Field-collected samples	No field-collected samples used in the study.
Ethics oversight	All the animals and samples used in this study were collected according to the guidelines for the care and use of experimental animals established by the Ministry of Agriculture of China.

Note that full information on the approval of the study protocol must also be provided in the manuscript.

Human research participants

Policy information about studies involving human research participants

Population characteristics	Three male patients were recruited. All of them are of Chinese descent with 20, 36, and 47-year-old age, respectively.
Recruitment	Patients were recruited from Sichuan Provincial People's Hospital with written informed consent. The possible biases of the ages are not likely to impact results, as this is for comparative analysis across species.
Ethics oversight	Collection and sequencing of human clinical samples were approved by the Ethics Committee of Sichuan Provincial People's Hospital, and informed consent was obtained before the study.

Note that full information on the approval of the study protocol must also be provided in the manuscript.
